# The Electrochemical *cis*‐Chlorination of Alkenes

**DOI:** 10.1002/chem.202103316

**Published:** 2021-10-20

**Authors:** Julia Strehl, Cornelius Fastie, Gerhard Hilt

**Affiliations:** ^1^ Institut für Chemie Universität Oldenburg Carl-von-Ossietzky-Straße 9–11 26111 Oldenburg Germany

**Keywords:** alkenes, *cis*-addition, chlorination, electrolysis, selenium

## Abstract

The first example for the electrochemical *cis*‐dichlorination of alkenes is presented. The reaction can be performed with little experimental effort by using phenylselenyl chloride as catalyst and tetrabutylammoniumchloride as supporting electrolyte, which also acts as nucleophilic reagent for the S_N_2‐type replacement of selenium versus chloride. Cyclic voltammetric measurements and control experiments revealed a dual role of phenylselenyl chloride in the reaction. Based on these results a reaction mechanism was postulated, where the key step of the process is the activation of a phenylselenyl chloride‐alkene adduct by electrochemically generated phenylselenyl trichloride. Like this, different aliphatic and aromatic cyclic and acyclic alkenes were converted to the dichlorinated products. Thereby, throughout high diastereoselectivities were achieved for the *cis*‐chlorinated compounds of >95 : 5 or higher.

## Graphical abstract

**Double role**: The *cis*‐chlorination of alkenes is realised in an electrochemical setup with PhSeCl as catalyst. The supporting electrolyte (TBACl) acts as nucleophilic reagent for the S_N_2‐type replacement of selenium versus chloride in the key step of the process while the PhSeCl‐alkene adduct is activated by electrochemically generated PhSeCl_3_.

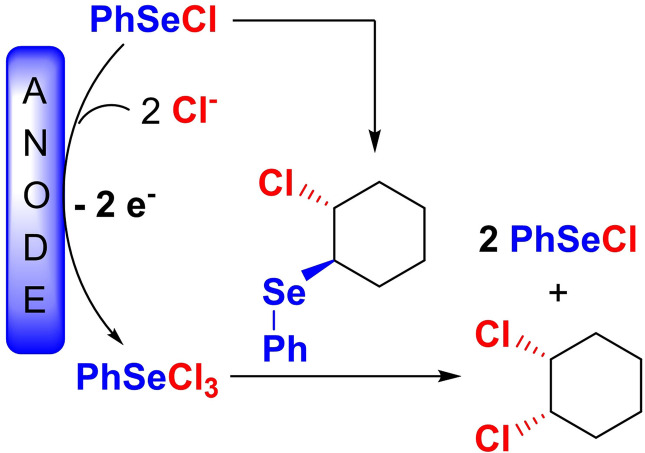

The electrophilic addition of dihalides to alkenes is a well‐understood process which leads, in the case of cyclic alkenes, in excellent yields and very often in high to exclusive diastereoselectivities to the *trans*‐dihalogenated products.^[1]^ In this respect, most likely, >90 % of the chemists world‐wide have performed the bromination of an alkene during their undergraduate laboratory education and a steadily increasing number of methods have been published to control the absolute stereochemistry of the *trans*‐addition.^[2]^ The academic spirit is a strong driving force which will sooner or later lead researchers to the question, how to realise a *cis*‐addition of dihalides to a carbon‐carbon double bond, regardless, if the addition can be realised on a cyclic or an acyclic alkene. In nature, a large number of acyclic 1,2‐dihalogenated natural products can be found which could be synthesised by a conventional *trans*‐addition of the dihalide either to the acyclic *E*‐ or the *Z*‐alkene.^[3]^ However, inspired by nature the enantioselective *trans*‐addition of dihalides to alkenes has attracted some attention.^[4]^ In a highly instructive review, *Denmark* analysed the scope and limitations of enantioselective *trans*‐addition of dihalides to alkenes and outlined the advances concerning the attempts to achieve a *cis*‐addition.^[5]^ In an exceptional investigation, the *Denmark* group demonstrated that the *cis*‐chlorination of alkenes can be realised (Scheme 1) utilising an oxidising agent (**3**), a simple selenium‐based catalyst, such as diphenyldiselenide, and a quenching agent (Me_3_SiCl) to remove potentially nucleophilic fluoride anions from the reaction mixture.^[6]^ The role of the pyridine‐*N*‐oxide derivative **4** was reported to enhance the rate of the reaction but was omitted in following reports. Later, chiral selenium species led only to moderate enantiomeric excess of the desired *cis*‐chlorinated products.^[7]^

**Scheme 1:**
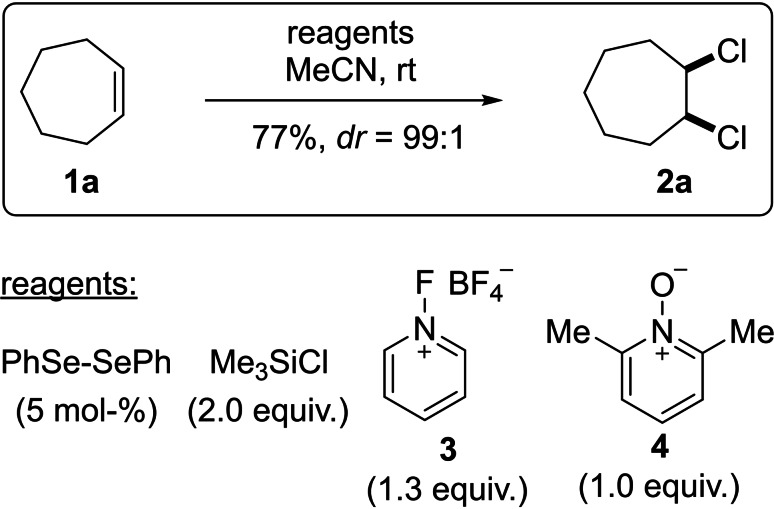
The *cis*‐chlorination of alkenes reported by Denmark.^[6]^

The analysis of the reaction mechanism based on investigations performed by *Denmark* and *Li* (Scheme 2)[^6^, ^8^] was as follows: a) the catalyst diphenyldiselenide needs to be oxidised to PhSeCl_3_ prior to the addition of the alkene (not shown). The PhSeCl_3_ is in equilibrium with PhSeCl_2_^+^ (+Cl^−^) (step **A**) and its interaction with the alkene will generate a selenonium ion **I** (step **B**).

**Scheme 2:**
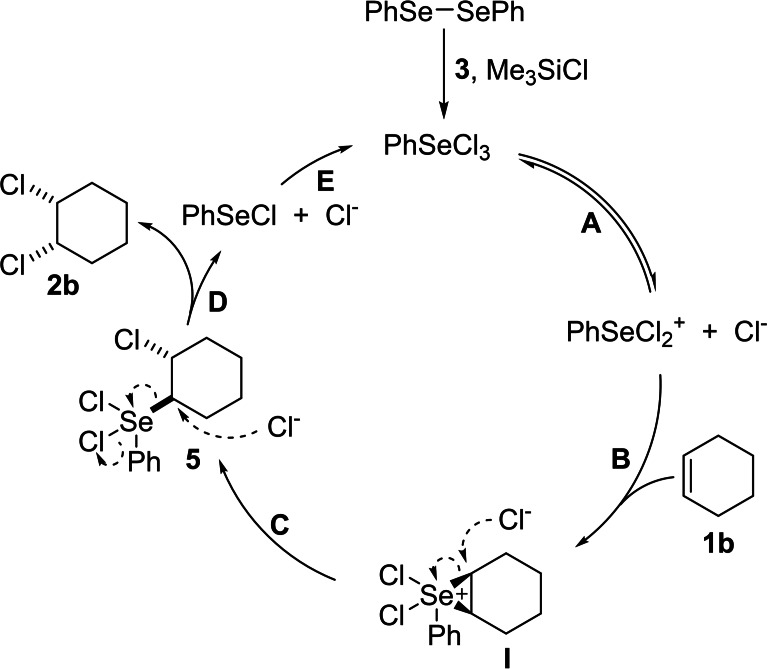
Proposed mechanism of the *cis*‐chlorination of cyclohexene.

This species will undergo a *trans*‐selective ring‐opening S_N_2‐type reaction (step **C**) with the chloride ion, just liberated from PhSeCl_3_, towards intermediate **5**.^[9]^ An additional chloride anion will undergo another S_N_2‐type reaction with the PhSeCl_2_‐moiety of intermediate **5** (step **D**) to produce the *cis*‐dichlorinated product,^[10]^ and liberates PhSeCl and a chloride anion. At last, the oxidation of PhSeCl regenerates the catalyst (step **E**). The alternative mechanism described by *Denmark*, considered the *trans*‐addition of PhSeCl to the alkene forming adduct **6**. This step is followed by the oxidation to the Se(IV) species **5** which then undergoes the substitution reaction (**6**→**5**→**2 b**) as shown in Scheme 3. The analysis of the reaction conditions prompted us to investigate an electrochemical version of this reaction and hopefully simplifying the needed reagents to a cost‐efficient and atom‐economic minimum.^[11]^ At that point, an alternative starting point under electrochemical conditions came into the perspective for the following reasons. Firstly, the reaction conditions can be simplified when the commercially available PhSeCl is applied as selenium source. Secondly, the rates of the two S_N_2‐type reactions with chloride anions outlined in Scheme 2 are likely to depend on the chloride concentration and the chloride concentration in the Denmark process is relatively low. Thirdly, the use of tetrabutylammonium chloride (TBACl) as supporting electrolyte would provide the needed conductivity for an electrochemical reaction as well as the relatively high concentration of chloride anions in solution.

**Scheme 3:**
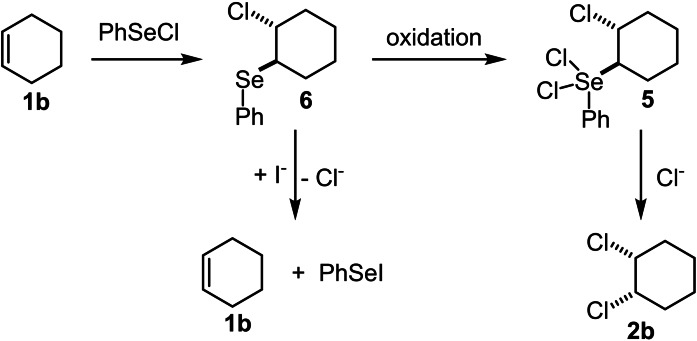
Alternative reaction pathway of adduct **6**.

In fact, the envisaged *cis*‐dichlorination could rely on the fast addition of PhSeCl^[6]^ to alkenes and the oxidation of the adduct towards intermediates of type **5** under mild electrochemical conditions. The electroanalytical behaviour of Ph_2_Se_2_ was reported by *Sasaki* and showed two irreversible oxidation peaks in acetonitrile at *E*_p_(ox)=+1.35 V and +1.65 V (vs. SCE) and a reduction peak at −1.25 V by cyclic voltammetry.^[12]^ Accordingly, the formation of a stable Ph_2_Se_2_‐radical cation by oxidative electron transfer was excluded and a Se−Se bond cleavage was proposed, especially when nucleophiles, such as water, were present. Therefore, when chloride anions from the supporting electrolyte are present, the formation of PhSeCl by an overall two‐electron oxidation of Ph_2_Se_2_ can be envisaged. In the literature, the efficient *trans*‐addition of PhSeCl to alkenes, such as cyclohexene, is reported.^[13]^ Astonishingly, this addition process to the alkene seems to be “reversible” in the presence of other halide anions, such as iodide, so that PhSeI and Cl^−^ are formed while the alkene is regenerated, as was reported by Goto (Scheme 3).^[14]^ Therefore, iodide anions will lead to the reverse process and cannot be applied as low‐redox potential mediator to oxidise the envisaged Se(II) intermediate **6** (Scheme 3) to a Se(IV) species **5**. This is believed to be needed for the second nucleophilic substitution reaction (compare Scheme 2, transformation of **5** in step **D**).

The use of bromide anions as redox mediator proved to be insufficient as well. The electrolysis of cyclohexene in the presence of PhSeBr, tetrabutylammonium chloride (TBACl) as supporting electrolyte and catalytic amounts (10 mol %) of the corresponding bromide salt (TBABr) led only to 18 % of the *cis*‐dichlorinated product as well as many side products including also 1‐bromo‐2‐chlorocyclohexane. Nevertheless, in the absence of the TBABr, the desired *cis*‐1,2‐dichlorocyclohexane product could be detected in moderate amounts (24 %). Therefore, we altered the starting point of the *cis*‐chlorination and used PhSeCl (1.0 equiv.) instead of PhSeBr and TBACl (4.0 equiv.) as supporting electrolyte. In an anodic oxidation process, the yield of the *cis*‐1,2‐dichlorocyclohexane product **2 b** could be increased to 32 %. With this encouraging result, we started the optimisation of the electrochemical reaction conditions leading to the *cis*‐dichlorination of cyclohexene towards product **2 b** which results are summarised in Table 1.

**Table 1:** Results of the electrochemical *cis*‐chlorination of cyclohexene.^[a]^

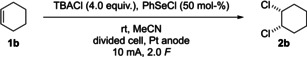		
Entry	Variations from above	Yield [%]^[b]^
1	none	39
2	N_2_ atmosphere	36
3	glassy carbon anode	38
4	graphite anode	33
5	3.0 mmol TBACl	48
6	4.0 mmol TBACl	48
7	5 mA	53
8	3.0 *F*	48
9	4.0 *F*	45
10	combination of previous parameters (5 mA, 3 mmol TBACl, 3.0 *F*)	60
11	DMF^[c]^	81
12	35 mol % PhSeCl	56 (+18 % *trans*)

[a] Unless otherwise stated, the reactions were performed in a divided cell with platinum electrodes (surface area: 1.0 ⋅ 3.0 cm^2^, depth of immersion: 16 mm, electrode distance: 6.5 cm) on a 0.5 mmol scale. [b] The yield was determined by GC‐FID analysis of the crude reaction mixture using mesitylene as internal standard. [c] This change was kept for entry 12.

At the beginning of the optimisation, the amount of PhSeCl was reduced to 50 mol %, which slightly increased the yield of **2 b** to 39 % (Table 1, entry 1). When the reaction was performed under inert conditions the yield decreased to 36 %. Later, neither a glassy carbon nor a graphite anode led to an improvement of the yield (Table 1, entries 3/4). When the amount of TBACl was increased to 3.0 mmol (Table 1, entry 5), product **2 b** was observed in 48 % yield. This result can be explained by the dependence of the rate of the reaction on the Cl^−^ concentration which has already been postulated above (see Scheme 2). But a further increase of the TBACl amount to 4.0 mmol did not have any consequence. When decreasing the current to 5 mA (Table 1, entry 8), the yield was further improved to 53 %. Also, the amount of current was investigated: While an improvement to 48 % yield was caused by the application of 3.0 *F* to the reaction mixture, the yield nearly stagnated for 4.0 *F* of current (Table 1, entries 9/10). Afterwards, the single optimised parameters were combined (Table 1, entry 11), so that cyclohexene was converted with 3.0 mmol TBACl applying 5 mA for 3.0 *F*, which resulted in an acceptable yield of 60 %. Afterwards, different solvents (e. g. DCM, acetone, NMP, see Supporting Information) were tested and DMF resulted in 81 % yield of compound **2 b** (Table 1, entry 12). For economic and ecological reasons, a further decrease of the amount of PhSeCl was investigated afterwards. But already when 35 mol % of PhSeCl were applied the yield of the *cis*‐chlorinated product **2 b** dropped to 56 % and 18 % of the *trans*‐product were observed. This observation should be kept in mind for mechanistic considerations.

At this point, alternative hypotheses were envisaged to explain the formation of **2 b** (Scheme 4).

**Scheme 4:**
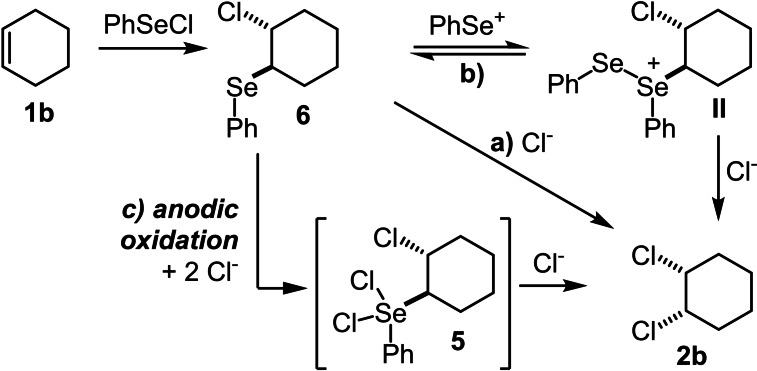
Possible reaction pathways of intermediate **6**.

1. Over‐stoichiometric amounts of PhSeCl alone are sufficient for the formation of the desired *cis*‐dichlorinated product **2 b** via intermediate **II** and the reaction is independent from any electrochemical interaction (Scheme 4a).
2. The side product (Ph_2_Se_2_) is electrochemically oxidised, formally to PhSe^+^, to initiate this process (Scheme 4b).
3. Adduct **6** is oxidised electrochemically to species **5**, which generates the product by substitution reaction (Scheme 4c).

The first two hypotheses were disproven by control experiments. No desired product **2 b** was formed when PhSeCl (50 mol %) and cyclohexene (**1 b**) were dissolved in acetonitrile (with or without TBACl) and stirred for 24 h. Also, when cyclohexene (**1 b**) was reacted with overstoichiometric amounts of PhSeCl (2.0 equiv.) and (20 mol %) of BCl_3_ or AgClO_4_, (for the in situ formation of PhSe^+^) the desired product **2 b** could not be detected. The postulated adduct **6** was reacted with PhSeCl itself, and also in combination with AgBF_4_, but again no product formation took place (Scheme 5). To assure the formation of adduct **6** as intermediate, it was electrolysed under the optimised reaction conditions and the product **2 b** could be observed in 52 %.

**Scheme 5:**
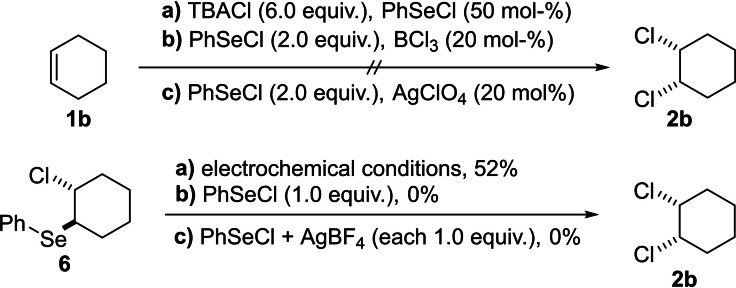
Control experiments for the dichlorination of alkenes.

To gain further insights, some cyclic voltammetric measurements were performed (Figure 1). Surprisingly, the redox potentials of TBACl, PhSeCl and adduct **6** are quite similar. While their on‐set potentials are around +800 mV, also the peak potentials do not differ significantly (TBACl: +1.09 V and +1.31 V, PhSeCl: +1.06 V, adduct **6**: +1.15 V). Consequently, these measurements do not predicate which compound is oxidised in the electrochemical set‐up. But for the mixture of TBACl and PhSeCl a peak current enhancement arises and the broad reduction peak of TBACl disappears. Then, the peak current is slightly further increased by the addition of adduct **6** which indicates a reaction between these species (Scheme 4c).

**Figure 1:**
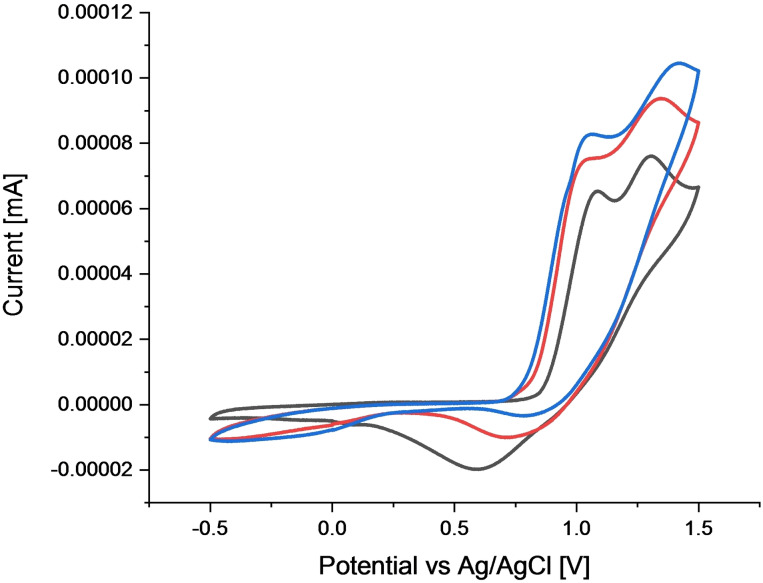
Cyclic voltammograms of a) black curve: TBACl (30 mM), b) red curve: TBACl (30 mM) and PhSeCl (2.5 mM); c) blue curve: TBACl (30 mM), PhSeCl (2.5 mM) and adduct **6** (5 mM) with LiClO_4_ (c=0.1 M) in DMF (10 mA); 50 mV ⋅ s^−1^, platinum, Ag/AgCl reference electrode.

To clarify these observations, two other control experiments were performed (Scheme 6). First, no product formation could be observed when the adduct **6** was reacted with TBACl and hydrogen peroxide as oxidant. But when the experiment was repeated and PhSeCl was added, product **2 b** was formed. Although, in only 27 % due to the *syn‐*elimination to the side‐product 3‐chlorocyclohex‐1‐ene. This indicates that PhSeCl is oxidised in the presence of chloride anions towards PhSeCl_3_ under electrochemical conditions. This also explains why *trans*‐**2 b** is observed at lower concentrations of PhSeCl: PhSeCl reacts with the alkene forming adduct **6** but also has to be present for the oxidation towards PhSeCl_3_ in relevant concentrations. Therefore, if the concentration of PhSeCl is too low, the electrochemically formed Cl^+^/Cl_2_ can directly react with the alkene forming the undesired *trans*‐isomer. For the verification of this hypothesis another control experiment was performed. When adduct **6** was stirred with PhSeCl_3_ in the presence of TBACl overnight, product **2 b** was observed in 69 % yield (Scheme 6).^[16]^

**Scheme 6:**
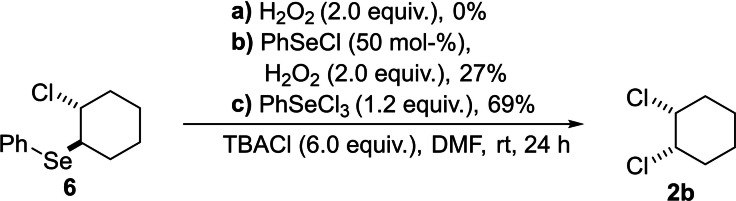
Further control experiments for the conversion of adduct **6**.

According to these results, the reaction mechanism of the electrochemical *cis*‐dichlorination of alkenes was proposed as outlined in Scheme 7.

**Scheme 7:**
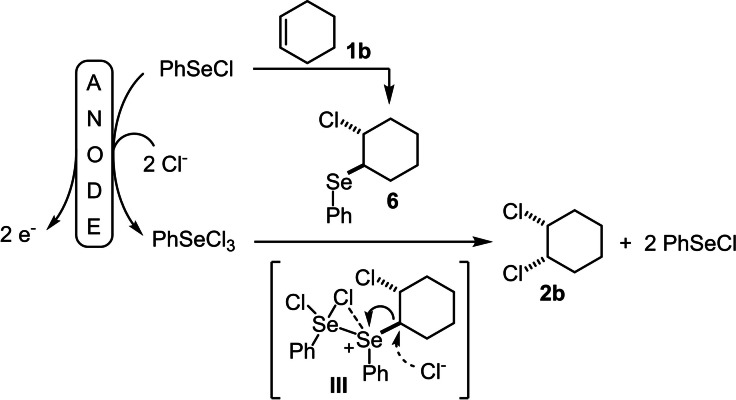
Proposed mechanism of the electrochemical PhSeCl‐mediated *cis*‐chlorination of alkenes.

The scenario that consolidates all the observations of the control experiments is as follows: First, the relatively slow electrochemical oxidation of PhSeCl in combination with two chloride anions takes place to form PhSeCl_3_. Simultaneously, PhSeCl reacts in a concurring reaction with cyclohexene and forms adduct **6**. As postulated by Denmark and Li (see Scheme 2), PhSeCl_3_ is in equilibrium with PhSeCl_2_^+^ and Cl^−^. The PhSeCl_2_^+^ cation is likely to be a stronger Lewis acid compared to PhSe^+^ (as proposed in the control experiments by the addition of Ag^+^) and activates the phenylselenyl‐substituent of adduct **6** for the nucleophilic displacement by the chloride anion via intermediate **III** leading to the *cis*‐chlorinated product **2 b** and two equivalents of PhSeCl.

With the earlier optimised reaction conditions in hand, the substrate scope and the limitations of the reaction were investigated for simple cyclic and acyclic starting materials (Scheme 8). After the electrochemical *cis*‐chlorination of cyclohexene gave good results, also larger cyclic alkenes, like cycloheptene and cyclooctene, were chlorinated in good yields (53 % and 69 % respectively). However, styrene derivatives could only be chlorinated in moderate yields. This observation is not surprising since styrene derivatives are quite redox labile and difficult to be electrochemically halogenated as shown in former investigations for the bromination of alkenes by our group.^[15]^ However, the chlorination of terminal aliphatic alkenes worked well. While **2 f** was isolated in 86 %, the bromo‐substituted product **2 g** was isolated in 68 %. Moreover, excellent yields were accomplished for the conversion of 9‐dodecen‐1‐ol and 5‐hexenyl acetate yielding **2 i** and **2 j** in 96 % and 99 % yield, respectively. In contrast, the *cis*‐chlorinated alkene **2 h** was isolated in only 25 %. Furthermore, alcohol functionalised internal alkenes were used as starting materials as well. While the aliphatic product **2 k** was isolated in only 22 %, the allylic alcohol yielded **2 l** in 47 % and 2‐allyl phenol was chlorinated in a high yield of 82 %. At last, the *cis*‐chlorinated product **2 n** of *E*‐chalcone was isolated in 32 %. Notably, for all substrates high diastereomeric ratios of >95 : 5 or higher were achieved.

**Scheme 8:**
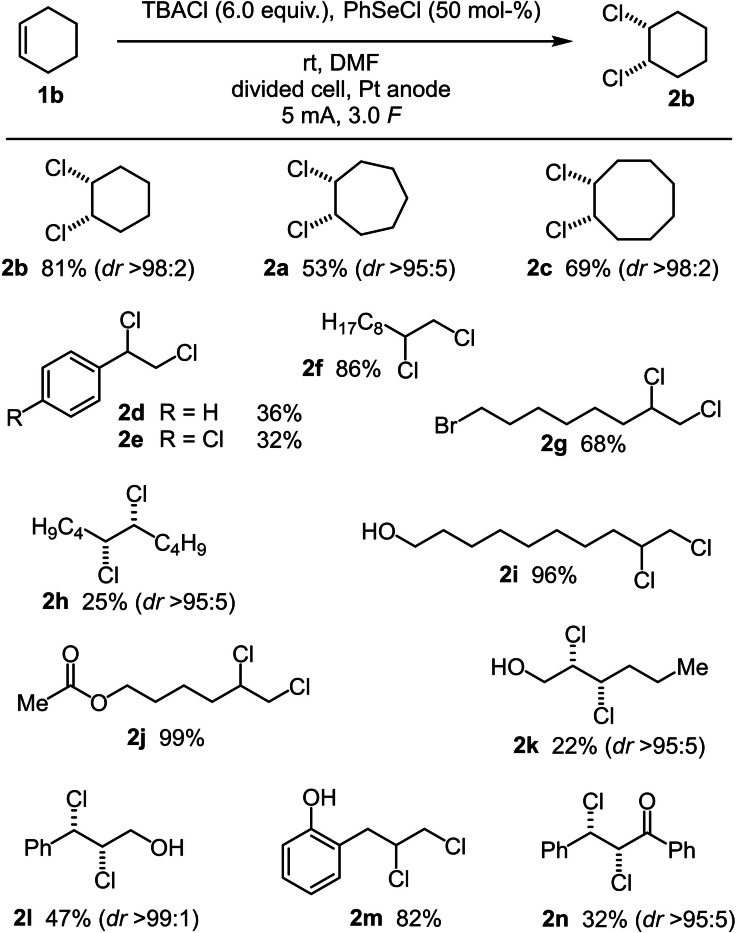
Scope of the electrochemical *cis*‐chlorination of alkenes.

In conclusion, we were able to develop and optimise the first electrochemical protocol for the *cis*‐dichlorination of alkenes. Furthermore, the scope and limitations of the reaction were investigated. At last, extensive mechanistical investigations by control experiments and cyclic voltammograms led to a proposed reaction mechanism, indicating that PhSeCl has a dual role in the electrochemical version of the *cis*‐chlorination of alkenes – the *trans*‐addition to the alkene and the formation of PhSeCl_3_ as activating agent for the S_N_2‐type reaction with the supporting electrolyte anion Cl^−^.

## Conflict of interest

The authors declare no conflict of interest.

## Supporting information

As a service to our authors and readers, this journal provides supporting information supplied by the authors. Such materials are peer reviewed and may be re‐organized for online delivery, but are not copy‐edited or typeset. Technical support issues arising from supporting information (other than missing files) should be addressed to the authors.

Supporting Information
