# Supplementary material for: The Electrochemical cis‐Chlorination of Alkenes
Source: Chemistry. 2021 Oct 20;27(69):17341–5. doi: 10.1002/chem.202103316 (PMC9297875; doi:10.1002/chem.202103316)
Supplement: Supplementary file 1 — Supporting Information [file CHEM-27-17341-s001.pdf]

Chemistry–A European Journal

Supporting Information

The Electrochemical *cis*-Chlorination of Alkenes

[a]J. Strehl, C. Fastie, Prof. Dr. G. Hilt
Institut für Chemie
Universität Oldenburg
Carl-von-Ossietzky-Straße 9–11, 26111 Oldenburg (Germany)
gerhard.hilt@uni-oldenburg.de
Supporting information for this article is available on the WWW under https://doi.org/10.1002/chem.202103316
© 2021 The Authors. Chemistry - A European Journal published by Wiley-VCH GmbH. This is an open access article under the terms of the Creative Commons Attribution License, which permits use, distribution and reproduction in any medium, provided the original work is properly cited.
Julia Strehl, Cornelius Fastie, and Gerhard Hilt*

Table of Content

1. General Information S1
2. Electrosynthesis S3
   2.1. Optimisation of the Reaction Conditions S3
   2.2. General Procedure (GP A) for the Electrochemical Conversions S4
   2.3. Synthesised Chlorinated Products S4
3. Mechanistic investigations S11
   3.1. Synthesis of 2-Chlorocyclohexylphenylselenide (2): S11
   3.2. Synthesis of Phenylselenyl trichloride S11
   3.3. Control Experiments S12
   3.4. Cyclic Voltammograms S14
4. Determination of the Relative Configurations of the Dichlorides S17
   4.1. Synthesis of the *trans*-Dichlorides S17
   4.2. Comparison of the GCMS analysis of the dichlorinated products S19
5. NMR spectra of the synthesised products S33
6. References S48

1. General Information

All solvents were commercially available and have been distilled under reduced pressure prior to use. Solvents were dried over 3 Å molecular sieves.

All chemicals or reagents were purchased from commercial suppliers without further purification, if not otherwise stated, or were prepared according to known literature procedures. If water or air sensitive compounds have been used, the experiments were carried out in heat gun dried glassware using conventional SCHLENK techniques under nitrogen atmosphere. Electrochemical reactions were carried out using an AIM-TTI Instruments MX100T power supply. These reactions were performed in a divided cell (Figure S1), equipped with a stirring bar, platinum electrodes ($1.0 \cdot 3.0\ cm^2$, depth of immersion: 16 mm, electrode distance: 65 mm). All known compounds were characterized by ^1H and ^{13}C NMR. All unknown compounds were identified by ^1H NMR, ^{13}C NMR, IR and HRMS.

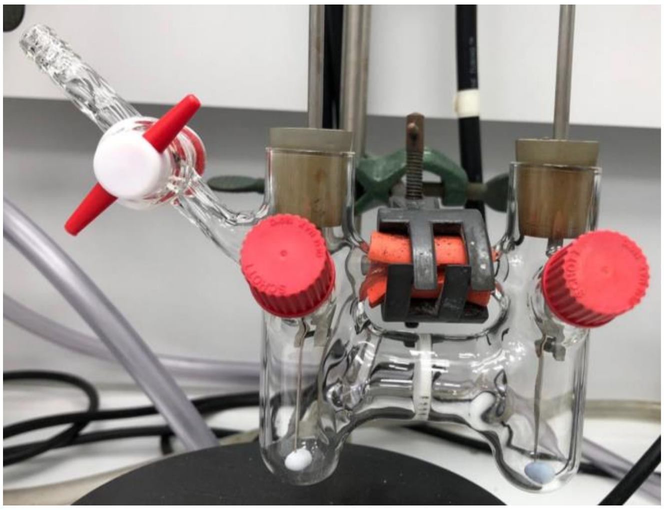

Figure S1: Divided electrolysis cell, equipped with platinum plate electrodes.

NMR spectroscopy: NMR spectra were recorded either on a Bruker Avance 300 (300 MHz), on a Bruker Avance III (500 MHz) or on a Bruker Avance DRX (500 MHz). Chemical shifts are reported in parts per million (ppm). The spectra are referenced to the residual solvent peak of $CDCl_3$. In the ^1H NMR spectra this corresponds with the singlet of the solvent signal of $CDCl_3$ at $\delta = 7.26$ ppm. The ^{13}C NMR spectra were referenced to the central line of the triplet of $CDCl_3$ at $\delta = 77.16$ ppm.[1] The stated form of the signal describes the appearance of the signal and not the theoretically expected form.

IR Spectroscopy: The IR spectra were obtained with a Shimadzu IRSpirit with a QATR-S cell. The wave numbers λ^{-1} are quoted in reciprocal centimetres (cm^{-1}).

Chromatography: Flash chromatography was carried out using Macherey-Nagel silica gel 60 (0.040-0.063 mm). Thin layer chromatography was carried out on Merck TLC plates coated

with silica gel 60 F_{254} with fluorescence indicator. For the detection of the signals ultraviolet light (λ = 254 nm) or GC analysis were used or heating after the plate has been dipped into a $KMnO_4$ solution.

MS/HRMS: MS and HRMS spectra of products were obtained with a Waters Q-TOF Premier (ESI, pos. mode or APCI) or Thermo Scientific DFS (EI) spectrometers.

2. Electrosynthesis

2.1. Optimisation of the Reaction Conditions

Table 1: Optimisation of the electrochemical *cis*-chlorination of alkenes.

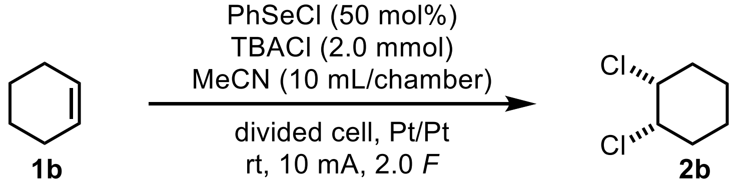

Entry	Variation from above	Yield[a]
1	None	39%
2	N_2 atmosphere	36%
	Variation of the anode material	
3	Glassy carbon	38%
4	graphite	33%
	Amount of TBACl	
5	**3.0 mmol TBACl**	**48%**
6	4.0 mmol TBACl	48%
	Current	
7	15 mA	32%
8	8 mA	45%
9	**5 mA**	**53%**
10	3 mA	50%
	Amount of current	
**11**	**3.0** *F*	**34%**
12	4.0 *F*	36%
	Solvent[b]	
13	MeCN	60%
14	CH_2Cl_2	33%
15	Aceton	0%
16	**DMF[c]**	**81%**
17	NMP	0%
	Reaction temperature[b]	
18	0 °C	31%
19	40 °C	67%
	Amount of PhSeCl[b]	
20	25 mol-%	41% *cis* (43% *trans*)
21	35 mol-%	56% *cis* (18% *trans*)
22	75 mol-%	85% only *cis*

23	25 mol-% PhSeSePh[b]	58% *cis* + 16% *trans*

The reactions were performed in a divided cell on a 0.5 mmol scale using platinum plate electrodes (surface area: 1.0 · 3.0 cm^2, depth of immersion: 16 mm, electrode distance: 65 mm) at room temperature. [a] The yield was determinded by GC-FID analysis of the crude reaction mixture using mesitylene as internal standard. [b] This optimisation experiments were performed applying the beforehand optimised reaction conditions: 5 mA, 3 mmol TBACl, 3.0 *F*. [c] This change was kept for entries 18-23.

2.2. General Procedure (GP A) for the Electrochemical Conversions

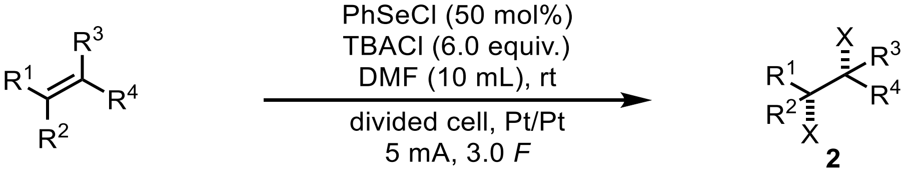

2

First, tetrabutylammonium chloride (TBACl) (3.00 mmol, 6.0 equiv.) was weighed into each chamber of a divided cell. Additionally, phenylselenyl chloride (0.25 mmol, 50 mol%) was weighed into the anodic chamber and dimethylformamide (10 mL/chamber) was added. After the addition of the corresponding alkene (0.50 mmol, 1.0 equiv.) to the anodic chamber, the reaction mixture was electrolysed under constant current (5 mA, 3.0 *F*, Pt electrodes) at room temperature and atmospheric moisture. The reaction mixture was diluted with saturated aqueous NH_4Cl solution (40 mL) and extracted with *n*-pentane (3 × 20 mL). The combined organic layers were dried ($MgSO_4$) and filtered. After removal of the solvent, the residue was submitted to column chromatography (SiO_2).

2.3. Synthesised Chlorinated Products

***cis*-1,2-Dichlorocyclohexane (2b):**

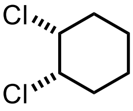

According to GP A, cyclohexene (41 mg, 0.50 mmol, 1.0 equiv.), phenylselenyl chloride (48 mg, 0.25 mmol, 0.5 equiv.) and tetrabutylammonium chloride (834 mg, 3.00 mmol, 6.0 equiv.) were converted to furnish product **2b** (61 mg, 0.34 mmol, 80%, *dr* >98:2) as colourless oil after column chromatography (SiO_2, *n*-pentane).

^{1}H NMR (500 MHz, $CDCl_3$): δ = 4.35-4.22 (m, 2H), 2.20-2.07 (m, 2H), 1.93-1.74 (m, 4H), 1.50-1.37 (m, 2H) ppm. **^{13}C NMR** (125 MHz, $CDCl_3$): δ = 62.9, 32.6 ppm. The analytical data are in accordance with the literature.[2]

cis-1,2-Dichlorocycloheptane (2a):

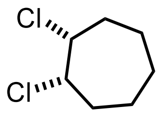

According to GP A, cycloheptene (48 mg, 0.50 mmol, 1.0 equiv.), phenylselenyl chloride (48 mg, 0.25 mmol, 0.5 equiv.) and tetrabutylammonium chloride (834 mg, 3.00 mmol, 6.0 equiv.) were converted to furnish product **2a** (45 mg, 0.27 mmol, 53%, *dr* >95:5) as colourless oil after column chromatography (SiO_2, *n*-pentane).

^{1}H NMR (500 MHz, $CDCl_3$): δ = 4.42-4.38 (m, 2H), 2.24-2.16 (m, 2H), 2.02-1.93 (m, 2H), 1.83-1.67 (m, 3H), 1.61-1.51 (m, 3H) ppm. **^{13}C NMR** (125 MHz, $CDCl_3$): δ = 66.4, 34.4, 25.8, 23.7 ppm. The analytical data are in accordance with the literature.[2]

cis-1,2-Dichlorocyclooctane (2c):

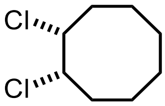

According to GP A, cyclooctene (75 mg, 0.50 mmol, 1.0 equiv.), phenylselenyl chloride (48 mg, 0.25 mmol, 0.5 equiv.) and tetrabutylammonium chloride (834 mg, 3.00 mmol, 6.0 equiv.) were converted to furnish product **2c** (63 mg, 0.35 mmol, 69%, *dr* >98:2) as colourless oil after column chromatography (SiO_2, *n*-pentane).

^{1}H NMR (500 MHz, $CDCl_3$): δ = 4.28 (dt, J = 3.0, 1.1 Hz, 2H), 2.31-2.24 (m, 2H), 2.07-1.99 (m, 2H), 1.90-1.82 (m, 2H), 1.73-1.67 (m, 2H), 1.61-1.52 (m, 2H), 1.47-1.35 (m, 2H) ppm. **^{13}C NMR** (125 MHz, $CDCl_3$): δ = 68.3, 33.4, 25.6, 25.2 ppm. The analytical data are in accordance with the literature.[2]

(1,2-Dichloroethyl)benzene (2d):

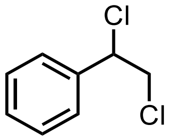

According to GP A, styrene (52 mg, 0.50 mmol, 1.0 equiv.), phenylselenyl chloride (48 mg, 0.25 mmol, 0.5 equiv.) and tetrabutylammonium chloride (834 mg, 3.00 mmol, 6.0 equiv.) were converted to furnish product **2d** (32 mg, 0.18 mmol, 36%) as colourless oil after column chromatography (SiO_2, *n*-pentane, R_f = 0.39).

^{1}H NMR (500 MHz, $CDCl_3$): δ = 7.45-7.34 (m, 5H), 5.01 (dd, J = 7.8, 6.6 Hz, 1H), 4.00 (dd, J = 11.4, 6.6 Hz, 1H), 3.93 (dd, J = 11.4, 7.9 Hz, 1H) ppm. **^{13}C NMR** (125 MHz, $CDCl_3$): δ = 138.2, 129.3, 129.0, 127.5, 61.9, 48.5 ppm. The analytical data are in accordance with the literature.[3]

1-Chloro-4-(1,2-dichloroethyl)benzene (2e):

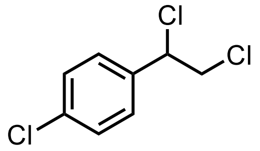

According to GP A, 4-chloro styrene (69 mg, 0.50 mmol, 1.0 equiv.), phenylselenyl chloride (48 mg, 0.25 mmol, 0.5 equiv.) and tetrabutylammonium chloride (834 mg, 3.00 mmol, 6.0 equiv.) were converted to furnish product **2e** (34 mg, 0.16 mmol, 32%.) as colourless oil after column chromatography (SiO_2, *n*-pentane, R_f = 0.36).

^{1}H NMR (500 MHz, $CDCl_3$): δ = 7.44-7.30 (m, 4H), 4.97 (dd, J = 8.4, 6.2 Hz, 1H), 3.99 (dd, J = 11.3, 6.2 Hz, 1H), 3.88 (dd, J = 11.3, 8.4 Hz, 1H) ppm. **^{13}C NMR** (125 MHz, $CDCl_3$): δ = 136.7, 135.2, 129.2, 129.0, 60.8, 48.2 ppm. The analytical data are in accordance with the literature.[3]

1,2-Dichlorodecane (2f):

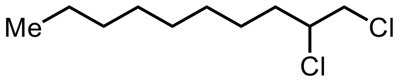

According to GP A, 1-decene (70 mg, 0.50 mmol, 1.0 equiv.), phenylselenyl chloride (48 mg, 0.25 mmol, 0.5 equiv.) and tetrabutylammonium chloride (834 mg, 3.00 mmol, 6.0 equiv.) were converted to furnish product **2f** (91 mg, 0.43 mmol, 86%) as colourless oil after column chromatography (SiO_2, *n*-pentane).

^{1}H NMR (500 MHz, $CDCl_3$): δ = 4.08-3.99 (m, 1H), 3.76 (dd, J = 11.3, 5.2 Hz, 1H), 3.65 (dd, J = 11.3, 7.4 Hz, 1H), 2.04-1.93 (m, 1H), 1.76-1.65 (m, 1H), 1.61-1.50 (m, 1H), 1.48-1.36 (m, 1H), 1.34-1.21 (m, 10H), 0.88 (t, J = 7.0 Hz, 3H) ppm. **^{13}C NMR** (125 MHz, $CDCl_3$): δ = 61.4, 48.4, 35.2, 32.0, 29.5, 29.3, 29.1, 26.0, 22.8, 14.2 ppm. The analytical data are in accordance with the literature.[3]

8-Bromo-1,2-dichlorooctane (2g):

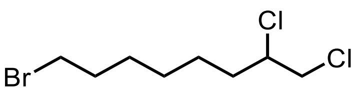

According to GP A, 8-bromo-1-octene (96 mg, 0.50 mmol, 1.0 equiv.), phenylselenyl chloride (48 mg, 0.25 mmol, 0.5 equiv.) and tetrabutylammonium chloride (834 mg, 3.00 mmol, 6.0 equiv.) were converted to furnish product **2g** (89 mg, 0.34 mmol, 68%) as colourless oil after column chromatography (SiO_2, *n*-pentane).

^{1}H NMR (500 MHz, $CDCl_3$): δ = 4.07-3.99 (m, 1H), 3.76 (dd, J = 11.3, 5.13 Hz, 1H), 3.64 (dd, J = 11.3, 7.5 Hz, 1H), 3.53 (t, J = 6.7 Hz, 2H), 2.04-1.95 (m, 1H), 1.83-1.68 (m, 3H), 1.63-1.53 (m, 1H), 1.50-1.32 (m, 5H) ppm. **^{13}C NMR** (125 MHz, $CDCl_3$): δ = 61.2, 48.3, 45.1, 35.0, 32.6,

28.4, 26.8, 25.8 ppm. **IR** (ATR): λ^{-1} = 2936 (m), 2860 (m), 1739 (w), 1463 (m), 1444 (m), 1433 (m), 1284 (m), 1230 (w), 1217 (w), 1018 (w), 918 (w), 813 (w), 726 (s), 654 (s), 623 (m) cm^{-1}. **GC/MS** (EI): m/z (%) = 144 (6), 139 (5), 118 (9), 117 (6), 109 (16), 106 (21), 105 (9) 104 (66), 103 (15), 102 (5), 95 (33), 93 (13), 91 (38), 90 (8), 89 (12), 83 (14), 81 (26), 79 (5), 77 (8), 75 (11), 70 (5), 69 (24), 68 (30), 67 (43), 65 (6), 63 (11), 56 (10), 55 (100), 54 (19), 53 (15), 51 (5). The HRMS analysis failed due to insufficient fragmentation of the compound.

***cis*-5,6-Dichlorodecane (2h):**

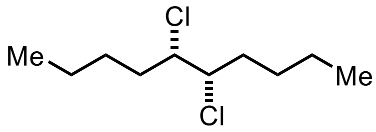

According to GP A, *E*-5-decene (70 mg, 0.50 mmol, 1.0 equiv.), phenylselenyl chloride (48 mg, 0.25 mmol, 0.5 equiv.) and tetrabutylammonium chloride (834 mg, 3.00 mmol, 6.0 equiv.) were converted to furnish product **2h** (26 mg, 0.12 mmol, 25%, *dr* >95:5) as colourless oil after column chromatography (SiO_2, *n*-pentane).

^{1}H NMR (500 MHz, $CDCl_3$): δ = 4.09-4.00 (m, 2H), 2.00-1.87 (m, 2H), 1.87-1.73 (m, 2H), 1.56-1.51 (m, 2H), 1.42-1.32 (m, 6H), 0.93 (t, *J* = 7.1 Hz, 6H) ppm. **^{13}C NMR** (125 MHz, $CDCl_3$): δ = 65.7, 34.3, 29.0, 22.3, 14.0 ppm. **IR** (ATR): λ^{-1} = 2957 (s), 2930 (s), 2873 (m), 2862 (m), 1467 (m), 1434 (m), 1380 (w), 1262 (w), 1240 (w), 1190 (w), 1123 (w), 1099 (w), 997 (w), 929 (w), 851 (w), 801 (w), 733 (m), 647 (s), 544 (w) cm^{-1}. **HRMS** (EI, 70 eV): calcd. 210.0937 ($C_{10}H_{20}Cl_2^+$), found 226.0940 [M$^+$].

9,10-Dichlorodecan-1-ol (2i):

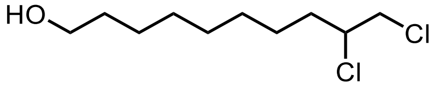

According to GP A, 9-decen-1-ol (78 mg, 0.50 mmol, 1.0 equiv.), phenylselenyl chloride (48 mg, 0.25 mmol, 0.5 equiv.) and tetrabutylammonium chloride (834 mg, 3.00 mmol, 6.0 equiv.) were converted to furnish product **2i** (111 mg, 0.489 mmol, 96%) as colourless oil after column chromatography (SiO_2, *n*-pentane:Et_2O = 2:1, R_f = 0.09).

^{1}H NMR (500 MHz, $CDCl_3$): δ = 4.06-4.00 (m, 1H), 3.76 (dd, *J* = 11.3, 5.2 Hz, 1H), 3.67-3.61 (m, 3H), 2.03-1.93 (m, 1H), 1.76-1.66 (m, 1H), 1.61-1.52 (m, 3H), 1.39-1.25 (m, 9H) ppm. **^{13}C NMR** (125 MHz, $CDCl_3$): δ = 63.1, 61.4, 48.4, 35.1, 32.9, 29.5, 29.4, 29.0, 25.9, 25.8 ppm. **IR** (ATR): λ^{-1} = 3342 (br), 2927 (s), 2854 (s), 1739 (w), 1464 (m), 1433 (m), 1372 (w), 1297 (w), 1260 (w), 1229 (w), 1217 (w), 1054 (s), 724 (s), 661 (s), 623 (m) cm^{-1}. **HRMS** (EI, 70 eV): calcd. 226.0886 ($C_{10}H_{20}OCl_2$), found 226.0885 [M$^+$].

5,6-Dichlorohexyl acetate (2j):

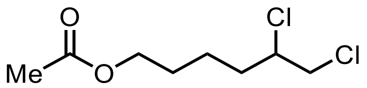

According to GP A, 5-hexenyl acetate (71 mg, 0.50 mmol, 1.0 equiv.), phenylselenyl chloride (48 mg, 0.25 mmol, 0.5 equiv.) and tetrabutylammonium chloride (834 mg, 3.00 mmol, 6.0 equiv.) were converted to furnish product **2j** (107 mg, 0.50 mmol, >99%) as colourless oil after column chromatography (SiO_2, *n*-pentane:Et_2O = 10:1, R_f = 0.23).

^{1}H NMR (500 MHz, $CDCl_3$): δ = 4.06 (t, J = 6.3 Hz, 2H), 4.04-3.98 (m, 1H), 3.75 (dd, J = 11.3, 5.1 Hz, 1H), 3.62 (dd, J = 11.3, 7.7 Hz, 1H), 2.03 (s, 3H), 2.02-1.98 (m, 1H), 1.78-1.59 (m, 4H), 1.54-1.44 (m, 1H) ppm. **^{13}C NMR** (125 MHz, $CDCl_3$): δ = 171.1, 64.1, 60.9, 48.1, 34.7, 28.1, 22.5, 21.0 ppm. The analytical data are in accordance with the literature.[4]

***cis*-2,3-Dichlorohexan-1-ol (2k):**

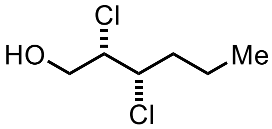

According to GP A, *E-2*-hexen-1-ol (50 mg, 0.50 mmol, 1.0 equiv.), phenylselenyl chloride (48 mg, 0.25 mmol, 0.5 equiv.) and tetrabutylammonium chloride (834 mg, 3.00 mmol, 6.0 equiv.) were converted to furnish product **2k** (19 mg, 0.11 mmol, 22%, *dr* >95:5) as colourless oil after column chromatography (SiO_2, *n*-pentane:Et_2O = 3:1, R_f = 0.34).

^{1}H NMR (500 MHz, $CDCl_3$): δ = 4.28-4.22 (m, 1H), 4.21-4.14 (m, 1H), 3.96 (dd, J = 5.8, 11.8 Hz, 1H), 3.89 (dd, J = 7.0, 11.8 Hz, 1H), 1.90-1.83 (m, 2H), 1.78 (brs, 1H), 1.66-1.54 (m, 1H), 1.50-1.39 (m, 1H), 0.96 (t, J = 7.4 Hz, 3H) ppm. **^{13}C NMR** (125 MHz, $CDCl_3$): δ = 65.6, 64.7, 61.9, 37.3, 20.0, 13.5 ppm.

IR (ATR): λ^{-1} = 3377 (brs), 2962 (s), 2934 (m), 2876 (m), 2363 (w), 1464 (m), 1382 (w), 1273 (w), 1260 (w), 1207 (w), 1080 (s), 1062 (s), 1045 (s), 1026 (s), 973 (m), 916 (w), 881 (w), 770 (m), 756 (m), 731 (m), 649 (m), 606 (w), 536 (w) cm^{-1}. **HRMS** (EI, 70 eV): calcd. 170.0260 ($C_6H_{12}OCl_2^+$), found 170.0264 [M^+].

***cis*-2,3-Dichloro-3-phenylpropan-1-ol (2l):**

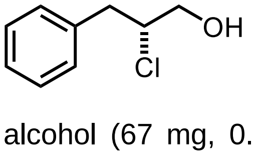

According to GP A, *trans*-cinnamyl alcohol (67 mg, 0.50 mmol, 1.0 equiv.), phenylselenyl chloride (48 mg, 0.25 mmol, 0.5 equiv.) and tetrabutylammonium chloride (834 mg, 3.00 mmol, 6.0 equiv.) were converted to furnish product **2l** (48 mg, 0.23 mmol, 47%, *dr* >99:1) as colourless oil after column chromatography (SiO_2, *n*-pentane:Et_2O = 4:1, R_f = 0.31).

^{1}**H NMR** (500 MHz, $CDCl_3$): *δ* = 7.46-7.33 (m, 5H), 5.10 (d, *J* = 9.4 Hz, 1H), 4.43 (ddd, *J* = 3.2, 4.8, 9.4 Hz, 1H), 4.18 (dd, *J* = 4.9, 12.3 Hz, 1H), 4.09 (dd, *J* = 3.1, 12.3 Hz, 1H) ppm. ^{13}**C NMR** (125 MHz, $CDCl_3$): *δ* = 138.3, 129.1, 128.8, 128.0, 66.2, 64.4, 61.6 ppm. **IR** (ATR): λ^{-1} = 3386 (br), 3033 (w), 2934 (w), 2882 (w), 1494 (w), 1454 (m), 1380 (w), 1186 (w), 1077 (m), 1057 (m), 966 (m), 923 (w), 896 (w), 840 (w), 829 (w), 729 (w), 694 (s), 674 (s), 633 (m), 600 (m), 549 (m), 516 (m) cm^{-1}. **HRMS** (EI, 70 eV): calcd. 204.0103 ($C_9H_{20}OCl_2^+$), found 204.0098 [M^+].

2-(2,3-Dichloropropyl)phenol (2m):

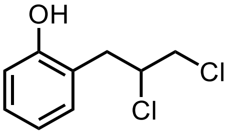

According to GP A, 2-allylphenol (67 mg, 0.50 mmol, 1.0 equiv.), phenylselenyl chloride (48 mg, 0.25 mmol, 0.5 equiv.) and tetrabutylammonium chloride (834 mg, 3.00 mmol, 6.0 equiv.) were converted to furnish product **2m** (84 mg, 0.41 mmol, 82%) as colourless oil after column chromatography (SiO_2, *n*-pentane:Et_2O = 20:1, R_f = 0.18).
^{1}**H NMR** (500 MHz, $CDCl_3$): *δ* = 7.20 (dd, *J* = 7.5, 1.2 Hz, 1H), 7.17 (dt, *J* = 7.7, 1.6 Hz, 1H), 6.92 (dt, *J* = 7.5, 1.0 Hz, 1H), 6.78 (dd, *J* = 8.0, 0.8 Hz, 1H), 4.96 (brs, 1H), 4.50-4.42 (m, 1H), 3.76 (dd, *J* = 5.5, 0.9 Hz, 2H), 3.35 (dd, *J* = 14.2, 6.1 Hz, 1H), 3.07 (dd, *J* = 14.2, 7.6 Hz, 1H) ppm. ^{13}**C NMR** (125 MHz, $CDCl_3$): *δ* = 153.8, 132.0, 128.8, 123.3, 121.2, 115.8, 60.4, 48.5, 36.7 ppm. The analytical data are in accordance with the literature.[5]

***cis*-2,3-Dichloro-1,3-diphenylpropan-1-one (2n):**

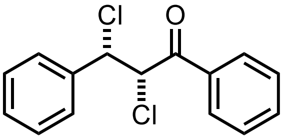

According to GP A, *trans*-chalcone (104 mg, 0.500 mmol, 1.0 equiv.), phenylselenyl chloride (48 mg, 0.25 mmol, 0.5 equiv.) and tetrabutylammonium chloride (834 mg, 3.00 mmol, 6.0 equiv.) were converted to furnish product **2n** (53 mg, 0.19 mmol, 38%, *dr* >95:5) as colourless oil after column chromatography (SiO_2, *n*-pentane:Et_2O = 10:1, R_f = 0.22).
^{1}**H NMR** (500 MHz, $CDCl_3$): *δ* = 8.08-8.04 (m, 2H), 7.66 (dt, *J* = 7.4, 1.2 Hz, 1H), 7.57-7.48 (m, 4H), 7.45-7.38 (m, 3H), 6.43 (d, *J* = 9.5 Hz, 1H), 5.42 (d, *J* = 9.5 Hz, 1H) ppm. ^{13}**C NMR** (125 MHz, $CDCl_3$): *δ* = 191.3, 135.7, 134.7, 134.4, 129.5, 129.1, 128.7, 128.2, 74.7, 56.2 ppm. The spectroscopic values are in accordance with literature values except the signal of one chlorinated CH-group at 74.7 ppm (vs. 60.3 ppm in the literature), which is rooted in the fact, that compound **4n** is the *cis*-isomer, whereas the literature describes the *trans*-isomer.[6]
IR (ATR): λ^{-1} = 3059 (w), 3037 (w), 3004 (w), 2940 (w), 1720 (s), 1682 (s), 1597 (s), 1580 (m), 1496 (w), 1457 (w), 1449 (w), 1381 (m), 1338 (w), 1310 (w), 1297 (w), 1277 (w), 1224 (m),

1180 (m), 1162 (m), 1146 (s), 1000 (w), 984 (m), 934 (w), 913 (w), 886 (w), 827 (m), 803 (w), 770 (m), 753 (m), 697 (s), 679 (s), 669 (s), 653 (s), 620 (w), 593 (s), 557 (s) cm^{-1}.

The analytical data are in accordance with the literature.[7,8]

3. Mechanistic investigations

3.1. Synthesis of 2-Chlorocyclohexylphenylselenide (6):

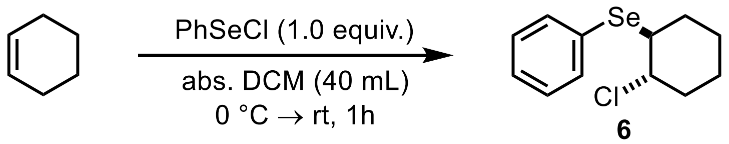

6

According to a literature procedure reported by Denmark,[9] cyclohexene (394 mg, 4.80 mmol, 1.2 equiv.) was dissolved under an inert atmosphere in abs. DCM (20 mL) and cooled down to 0 °C. At this temperature, a solution of phenylselenyl chloride (766 mg, 4.0 mmol, 1.0 equiv.) in abs. DCM (20 mL) was added over a period of 3 minutes. After the reaction mixture has been stirred for 10 minutes at 0 °C, it was allowed to warm to room temperature and stirred for further 50 minutes. Afterwards, all volatile compounds were removed in vacuum, yielding the crude product **6**.

^{1}H NMR (500 MHz, $CDCl_3$): δ = 7.65-7.57 (m, 2H), 7.36-7.28 (m, 3H), 4.26-4.15 (m, 1H), 3.57-3.46 (m, 1H), 2.43-2.35 (m, 1H), 2.33-2.26 (m, 1H), 1.87-1.77 (m, 2H), 1.77-1.69 (m, 1H), 1.69-1.61 (m, 1H), 1.53-1.41 (m, 2H) ppm. **^{13}C NMR** (125 MHz, $CDCl_3$): δ = 135.2, 129.2, 128.7, 128.0, 63.2, 49.3, 33.5, 30.5, 24.2, 22.8 ppm. The analytical data are in accordance with the literature.[9]

3.2. Synthesis of Phenylselenyl trichloride

According to a literature procedure of PRITCHARD,[10] Diphenyl diselenide (1.00 g, 3.20 mmol) was dissolved in abs. Et_2O (40 mL) under inert atmosphere. After addition of sulfuryl chloride (0.77 mL, 9.60 mmol), the solution was stirred for one hour at room temperature. The precipitated solid was filtered and dried in vacuo. The product (1.20 g, 4.61 mmol, 69%) was isolated as yellowish solid.

3.3. Control Experiments

To gain information about the reaction mechanism, some control experiments were performed. First, cyclohexene should be converted to 1,2-dichlorohexane (**2b**).

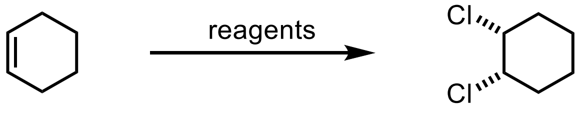

2b

Experiment 1: First, tetrabutylammonium chloride (TBACl) (834 mg, 3.00 mmol, 6.0 equiv.) was weighed into a round bottom flask. Additionally, phenylselenyl chloride (48 mg, 0.25 mmol, 50 mol%) was weighed into the anodic chamber and dimethylformamide (10 mL) was added. After the addition of cyclohexene (41 mg, 0.50 mmol, 1.0 equiv.), the reaction mixture was stirred at room temperature for 24 hours, but no product formation was observed.

Experiment 2: First, cyclohexene (21 mg, 0.25 mmol, 1.0 equiv.) was weighed into a round bottom flask and dissolved in abs. MeCN (10 mL). Afterwards, phenylselenyl chloride (96 mg, 0.5 mmol, 2 equiv.) and boron trichloride (1 M in *n*-hexane, 0.05 mmol, 20 mol%) were added. After the reaction mixture has been stirred at room temperature for 24 hours, no product formation was observed.

Experiment 3: First, cyclohexene (21 mg, 0.25 mmol, 1.0 equiv.) was weighed into a round bottom flask and dissolved in abs. MeCN (10 mL). Afterwards, phenylselenyl chloride (96 mg, 0.5 mmol, 2 equiv.) and silver perchlorate (10 mg, 0.05 mmol, 20 mol%) were added. After the reaction mixture has been stirred at room temperature for 24 hours, no product formation was observed.

Secondly, some control experiments to convert the postulated intermediate species **6** to **2b** were performed as well.

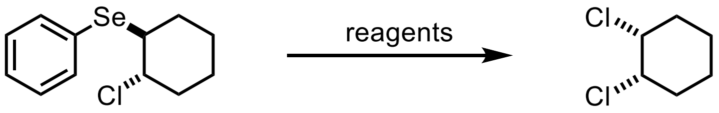

6 **2b**

Experiment 4: First, compound **6** was dissolved in abs. CH_2Cl_2 (5 mL) under inert atmosphere. Afterwards, phenylselenyl chloride (109 mg, 0.569 mmol, 1.0 equiv.) was added and the reaction mixture was stirred for 1 h at room temperature, but no product formation was observed via GCMS analysis. Then, silver tetrafluoroborate (111 mg, 0.570 mmol, 1.0 equiv.) was added and the mixture was stirred for another hour at room temperature. However, no

product **2b** could be observed. Again, TBACl (158 mg, 0.568 mmol, 1.0 equiv.) was added and after stirring at room temperature for 1 hour no product formation took place.

Experiment 5: First, tetrabutylammonium chloride (TBACl) (834 mg, 3.00 mmol, 6.0 equiv.) was weighed into each chamber of a divided cell. Additionally, phenylselenyl chloride (48 mg, 0.25 mmol, 50 mol%) was weighed into the anodic chamber and dimethylformamide (10 mL/chamber) was added. After the addition of the adduct **6** (137 mg, 0.50 mmol, 1.0 equiv.) and *n*-dodecane as internal standard (0.25 mmol, 1 M in $CHCl_3$) to the anodic chamber, the reaction mixture was electrolysed under constant current (5 mA, 2.0 *F*, Pt electrodes) at room temperature and atmospheric moisture. The yield of the product **2b** was analysed by GC-FID analysis of the crude reaction using mesitylene as internal standard mixture and amounted to 52%.

Experiment 6: First, adduct **6** (27 mg, 0.10 mmol, 1.0 equiv.) and TBACl (167 mg, 0.600 mmol, 6.0 equiv.) were dissolved in DMF (2 mL). Then, an aqueous hydrogen peroxide solution (0.20 mmol, 2 equiv.) was added and the reaction mixture was stirred at room temperature overnight. But no product formation of **2b** was observed.

Experiment 7: First, adduct **6** (27 mg, 0.10 mmol, 1.0 equiv.), PhSeCl (10 mg, 0.05 mmol, 0.5 equiv.) and TBACl (167 mg, 0.600 mmol, 6.0 equiv.) were dissolved in dimethylformamide (2 mL). Then, an aqueous hydrogen peroxide solution (0.20 mmol, 2 equiv.) was added and the reaction mixture was stirred at room temperature overnight. The yield of the product **2b** was analysed by GC-FID analysis of the crude reaction mixture using mesitylene as internal standard and amounted to 27%.

Experiment 8: First, adduct **6** (27 mg, 0.10 mmol, 1.0 equiv.) and TBACl (167 mg, 0.600 mmol, 6.0 equiv.) were dissolved in dimethylformamide (2 mL). Then $PhSeCl_3$ (32 mg, 0.12 mmol, 1.2 equiv.) was added and the solution was stirred overnight. The yield of product **2b** was analysed by GC-FID analysis of the crude reaction mixture using mesitylene as internal standard and amounted to 69%.

Experiment 9: First, adduct **6** (27 mg, 0.10 mmol, 1.0 equiv.) was dissolved in dimethylformamide (2 mL). Then $PhSeCl_3$ (32 mg, 0.12 mmol, 1.2 equiv.) was added and the solution was stirred overnight. The yield of product **2b** was analysed by GC-FID analysis of the crude reaction mixture using mesitylene as internal standard and amounted to 5%.

3.4. Cyclic Voltammograms

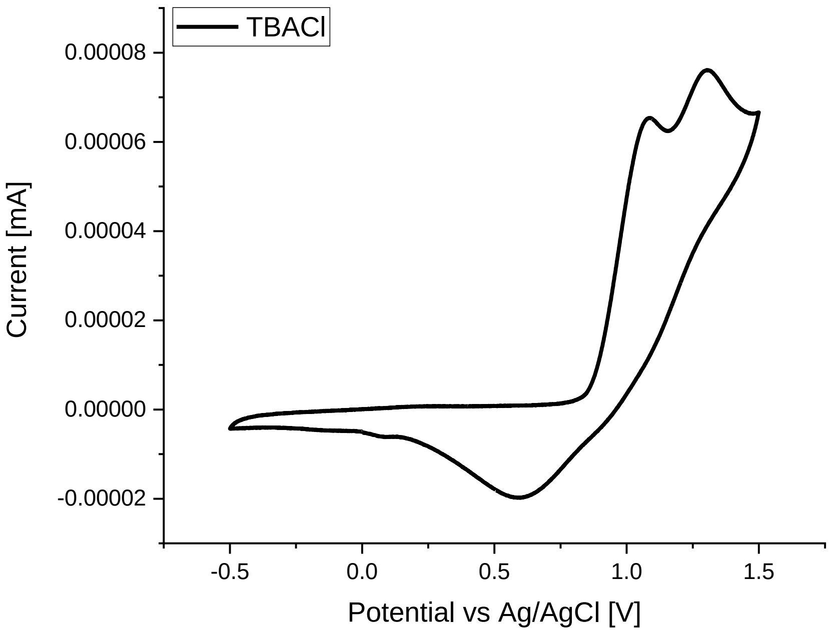

Figure 2: Cyclic voltammogram of TBACl (30 mM) in DMF (10 mL) with $LiClO_4$ (c = 0.1 M). Pt (2 mm diameter), Pt wire, Ag/AgCl (3 M KCl), scan rate = 50 $mV \cdot s^{-1}$.

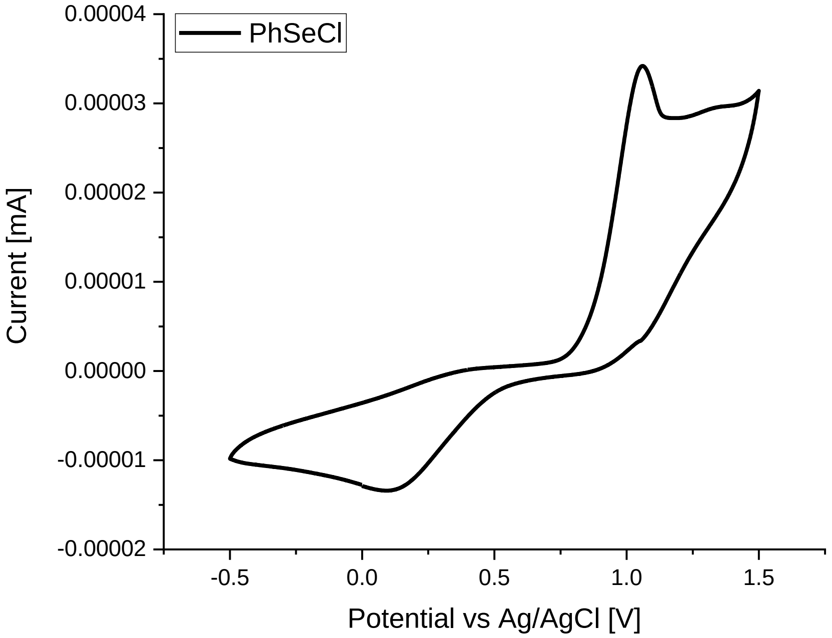

Figure 3: Cyclic voltammogram of PhSeCl (2.5 mM) in DMF (10 mL) with $LiClO_4$ (c = 0.1 M). Pt (2 mm diameter), Pt wire, Ag/AgCl (3 M KCl), scan rate = 50 $mV \cdot s^{-1}$.

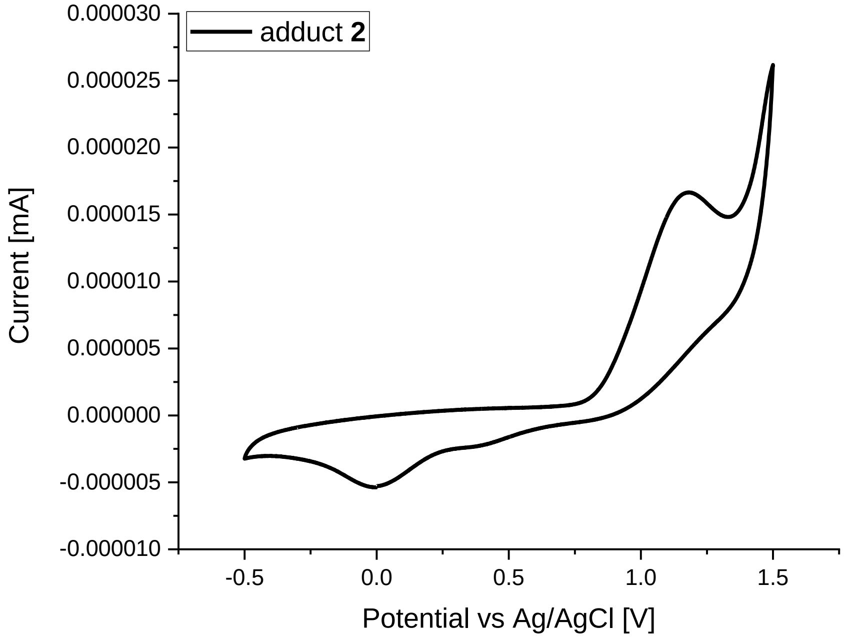

Figure 4: Cyclic voltammogram of adduct **6** (5 mM) in DMF (10 mL) with $LiClO_4$ (c = 0.1 M). Pt (2 mm diameter), Pt wire, Ag/AgCl (3 M KCl), scan rate = 50 mV·s^{-1}.

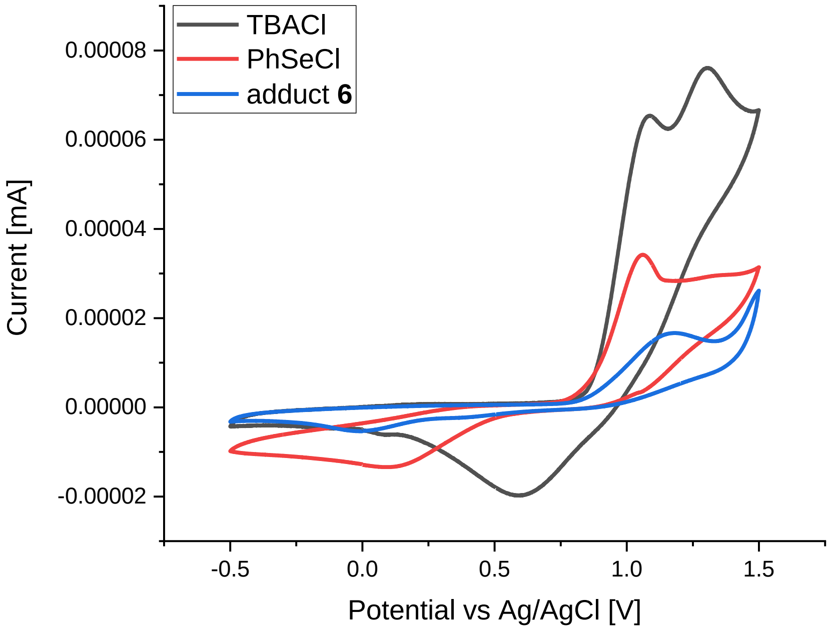

Figure 5: Cyclic voltammogram of a) black curve: TBACl (30 mM), b) red curve: PhSeCl (2.5 mM) and c) blue curve: adduct **6** (5 mM) in DMF (10 mL) with $LiClO_4$ (c = 0.1 M).
Pt (2 mm diameter), Pt wire, Ag/AgCl (3 M KCl), scan rate = 50 mV·s^{-1}.

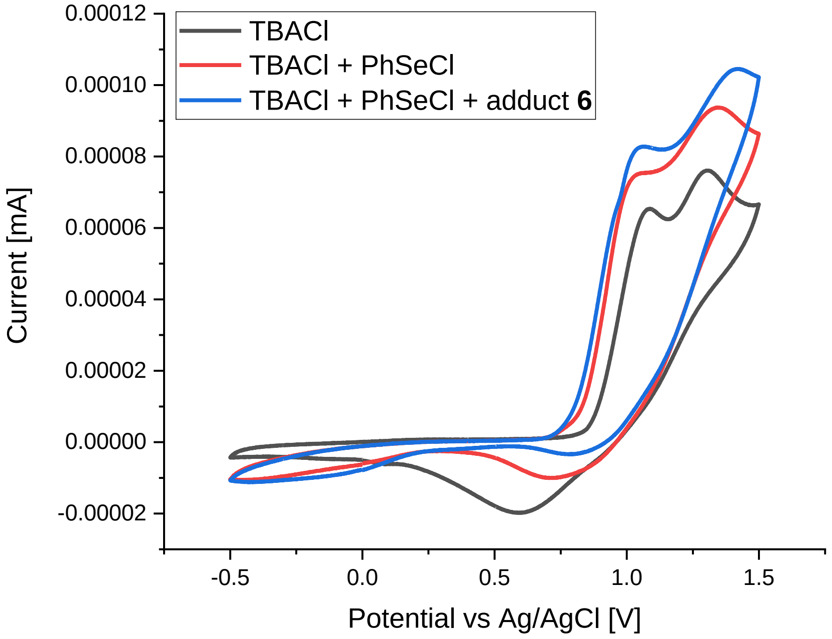

Figure 6: Cyclic voltammogram of a) black curve: TBACl (30 mM), b) red curve: TBACl (30 mM) and PhSeCl (2.5 mM) and c) blue curve: TBACl (30 mM), PhSeCl (2.5 mM) and adduct **6** (5 mM) in DMF (10 mL) with $LiClO_4$ (c = 0.1 M). Pt (2 mm diameter), Pt wire, Ag/AgCl (3 M KCl), scan rate = 50 mV·s^{-1}.

4. Determination of the Relative Configurations of the Dichlorides

The relative configurations of the dichlorides prepared via the presented electrochemical *cis*-dichlorination were determined by comparison of the GCMS spectra to those from the synthesised diastereomeric *trans*-dichlorides.

4.1. Synthesis of the *trans*-Dichlorides

General Procedure (GP B) for the *trans*-dichlorination of alkenes with oxone:

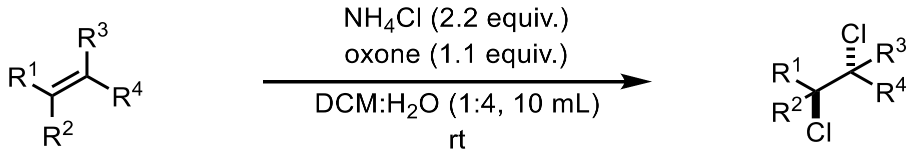

First, oxone (2.2 mmol, 1.1 equiv.) was slowly added to a solution of ammonium chloride (2.2 mmol, 2.2 equiv.) and the corresponding alkene (2 mmol, 1.0 equiv.) in a mixture of CH_2Cl_2:H_2O (1:4, 10 mL). After complete addition of the oxidant, the reaction mixture was stirred until total consumption of the olefin was detected. Afterwards, the organic layer was separated. The aqueous layer was extracted with CH_2Cl_2 (2 × 15 mL). The combined organic layers were dried ($MgSO_4$), filtered and concentrated. GCMS analysis was performed afterwards of the crude reaction mixture.

***trans*-1,2-Dichlorocyclohexane (*trans*-2b):**

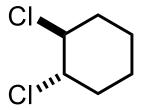

According to GP B, cyclohexene (164 mg, 2.00 mmol, 1.0 equiv.), ammonium chloride (235 mg, 4.40 mmol, 2.2 equiv.) and oxone (1.35 g, 2.20 mmol, 1.1 equiv.) were converted to furnish product ***trans*-2b**.

***trans*-1,2-Dichlorocycloheptane (*trans*-2a):**

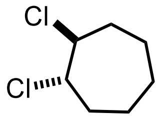

According to GP B, cycloheptene (96 mg, 2.0 mmol, 1.0 equiv.), ammonium chloride (235 mg, 4.40 mmol, 2.2 equiv.) and oxone (1.35 g, 2.20 mmol, 1.1 equiv.) were converted to furnish product ***trans*-2a**.

***trans*-1,2-Dichlorocyclooctane (*trans*-2c):**

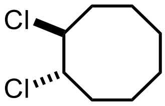

According to GP B, cyclooctene (220 mg, 2.00 mmol, 1.0 equiv.), ammonium chloride (235 mg, 4.40 mmol, 2.2 equiv.) and oxone (1.35 g, 2.20 mmol, 1.1 equiv.) were converted to furnish product ***trans*-2c**.

***trans*-5,6-Dichlorodecane (*trans*-2h):**

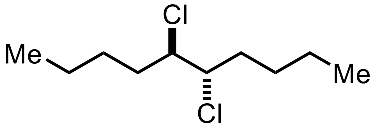

According to GP B, *E*-5-decene (140 mg, 2.00 mmol, 1.0 equiv.), ammonium chloride (235 mg, 4.40 mmol, 2.2 equiv.) and oxone (1.35 g, 2.20 mmol, 1.1 equiv.) were converted to furnish product ***trans*-2h**.

***trans*-2,3-Dichlorohexan-1-ol (*trans*-2k):**

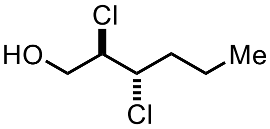

According to GP B, cyclooctene (100 mg, 1.00 mmol, 1.0 equiv.), ammonium chloride (118 mg, 2.20 mmol, 2.2 equiv.) and oxone (676 g, 1.10 mmol, 1.1 equiv.) were converted to furnish product ***trans*-2k**.

***trans*-2,3-Dichloro-3-phenylpropan-1-ol (*trans*-2l):**

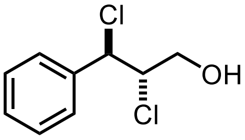

According to GP B, cyclooctene (134 mg, 1.00 mmol, 1.0 equiv.), ammonium chloride (118 mg, 2.20 mmol, 2.2 equiv.) and oxone (676 g, 1.10 mmol, 1.1 equiv.) were converted to furnish product ***trans*-2l**.

***trans*-2,3-Dichloro-1,3-diphenylpropan-1-one (*trans*-2m):**

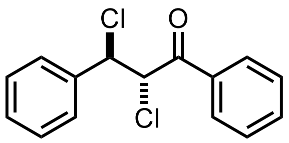

According to a literature procedure,[11] *trans*-chalcon (208 mg, 1.00 mmol, 1.0 equiv.) was placed into a round bottom flask under inert atmosphere. After the addition of sulfuryl chloride (2 mL) and stirring for 30 min at room temperature, all volatiles were removed in vacuo to furnish the crude product ***trans*-2m**.

4.2. Comparison of the GCMS analysis of the dichlorinated products

1,2-Dichlorocyclohexane:

***trans*-1,2-Dichlorocyclohexane (*trans*-2b):**

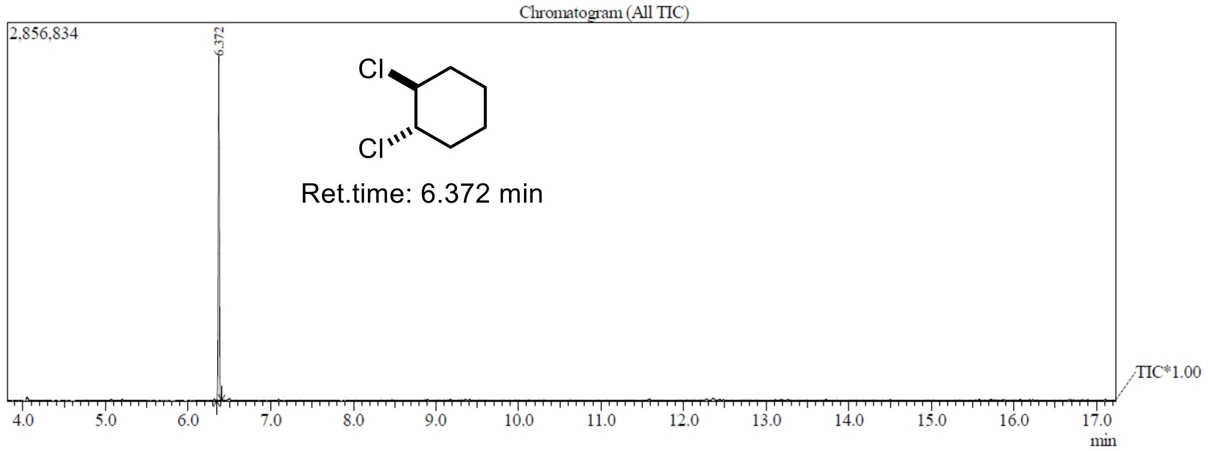

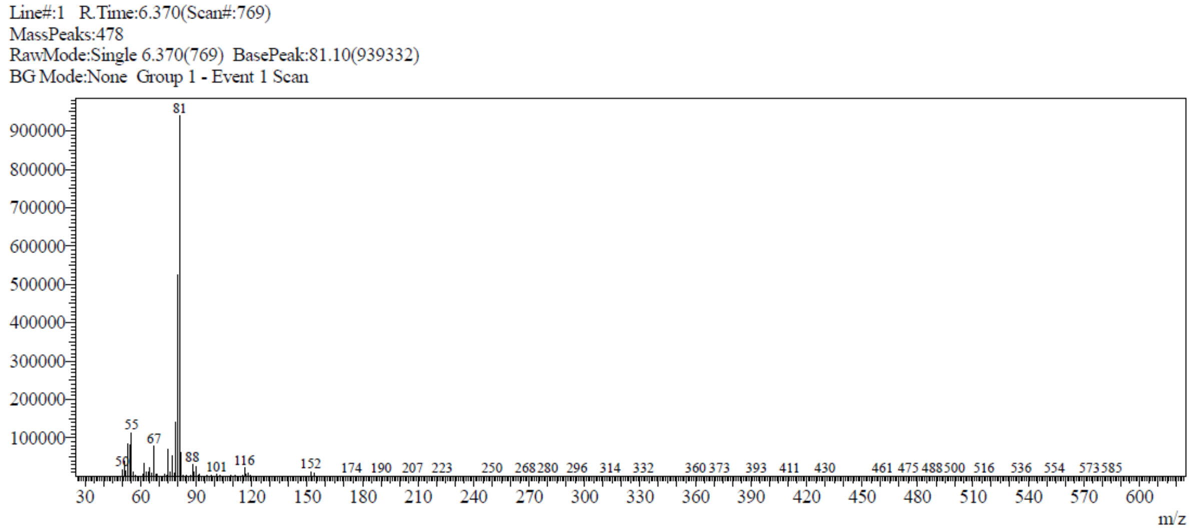

cis-1,2-Dichlorocyclohexane (2b):

C:\GCMSsolution\Data\Project1\ATCF-FP-060-t3_2.qgd

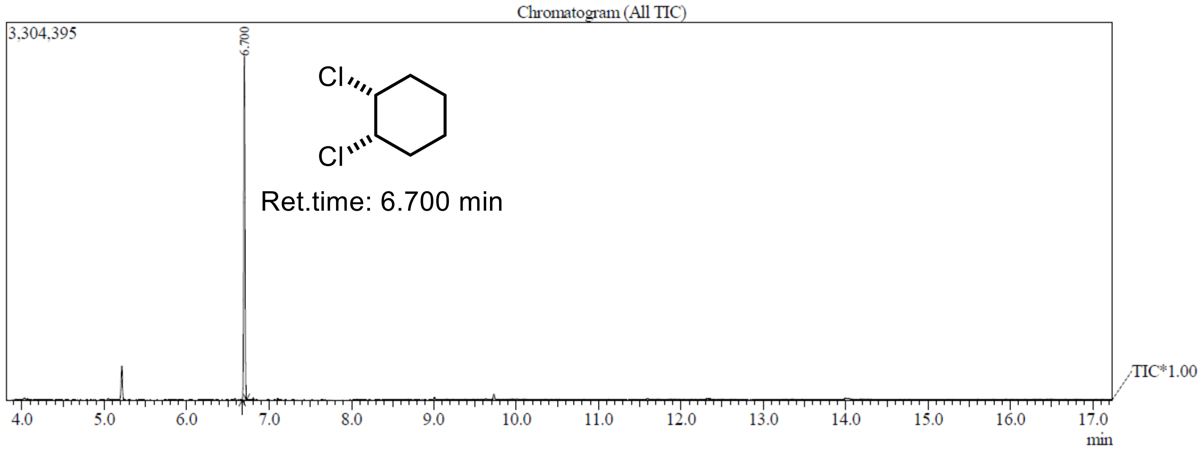

Line#:1 R.Time:6.700(Scan#:868)
MassPeaks:517
RawMode:Single 6.700(868) BasePeak:81.10(1069623)
BG Mode:None Group 1 - Event 1 Scan

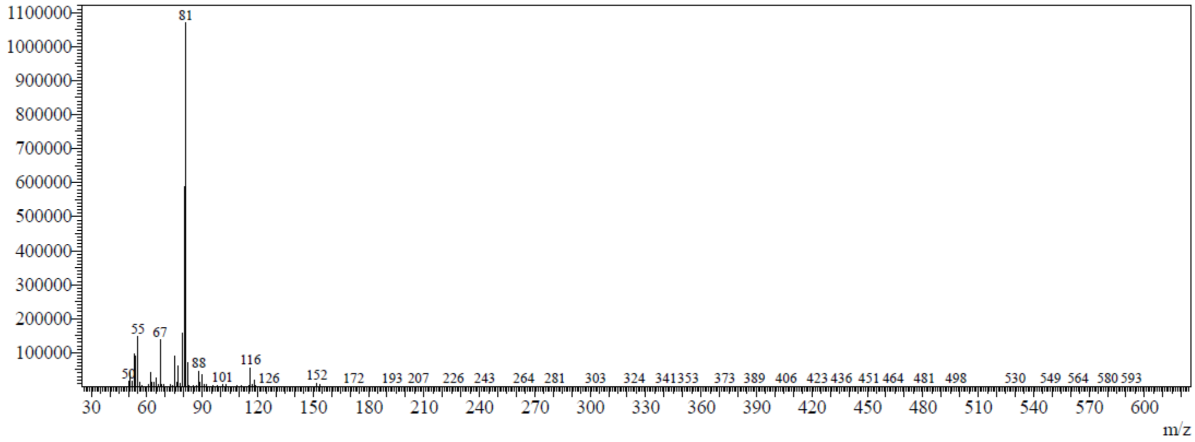

1,2-Dichlorocycloheptene:

trans-1,2-Dichlorocycloheptene (*trans*-2a):

C:\GCMSsolution\Data\Project1\JUST-1375_2.qgd

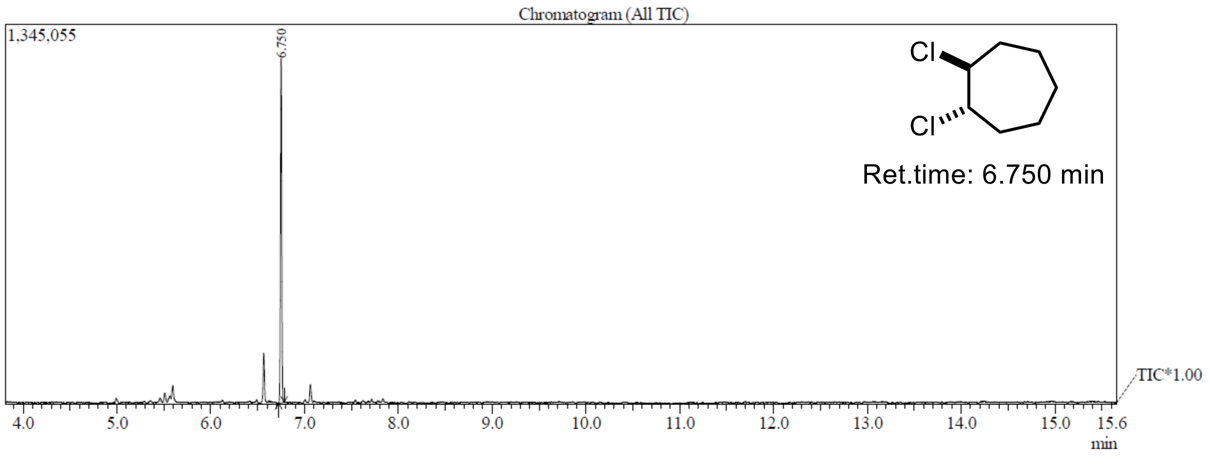

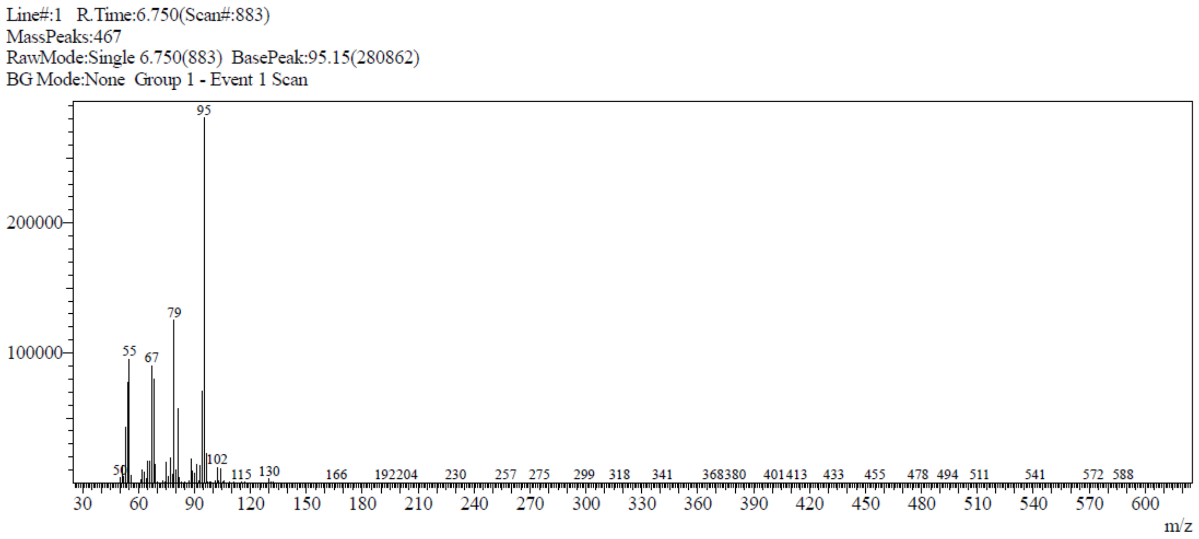

cis-1,2-Dichlorocycloheptene (2a):

C:\GCMSsolution\Data\Project1\JUST-1270-1315_2.qgd

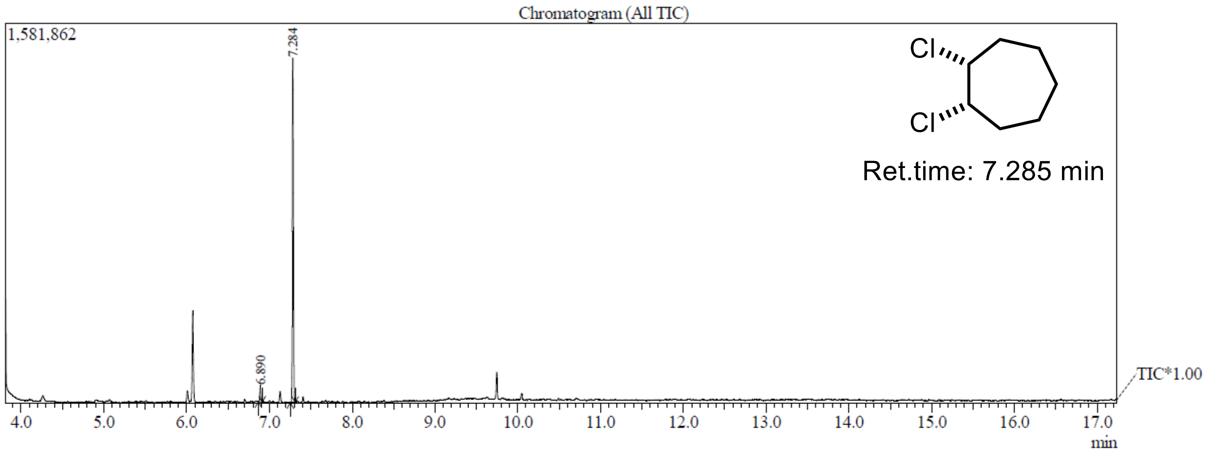

Line#:1 R.Time:7.283(Scan#:1043)
MassPeaks:496
RawMode:Single 7.283(1043) BasePeak:95.10(280498)
BG Mode:None Group 1 - Event 1 Scan

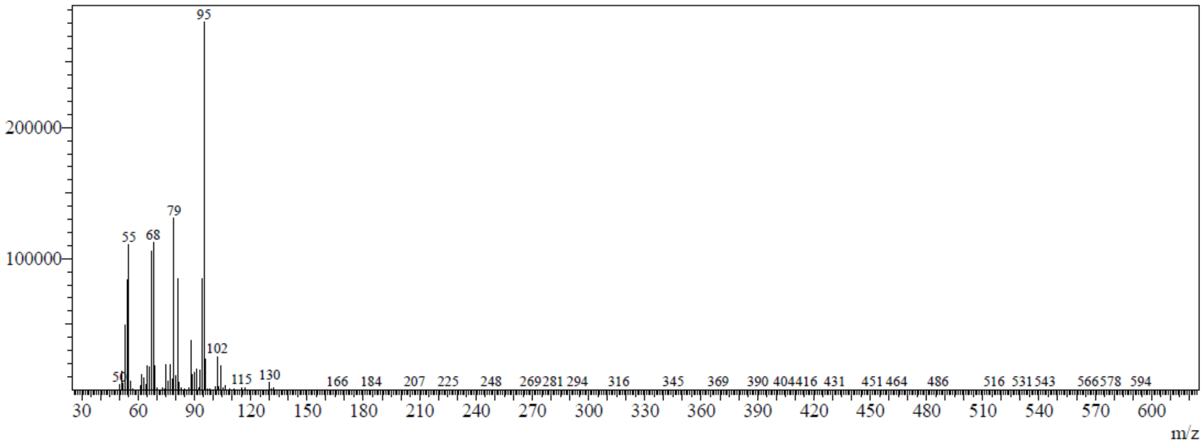

1,2-Dichlorocyclooctene:

***trans*-1,2-Dichlorooctene (*trans*-2c):**

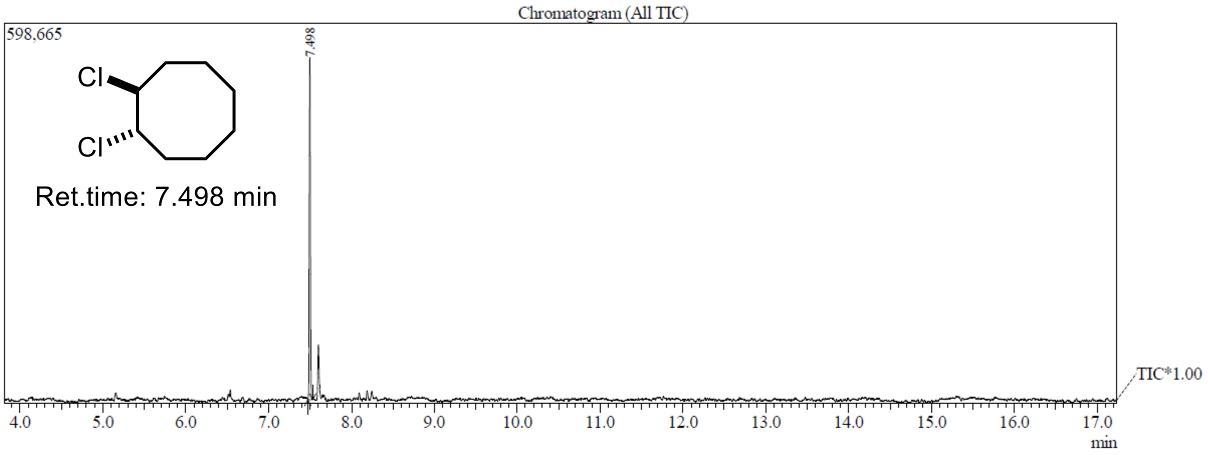

Line#:1 R.Time:7.500(Scan#:1108)
MassPeaks:430
RawMode:Single 7.500(1108) BasePeak:67.05(82708)
BG Mode:None Group 1 - Event 1 Scan

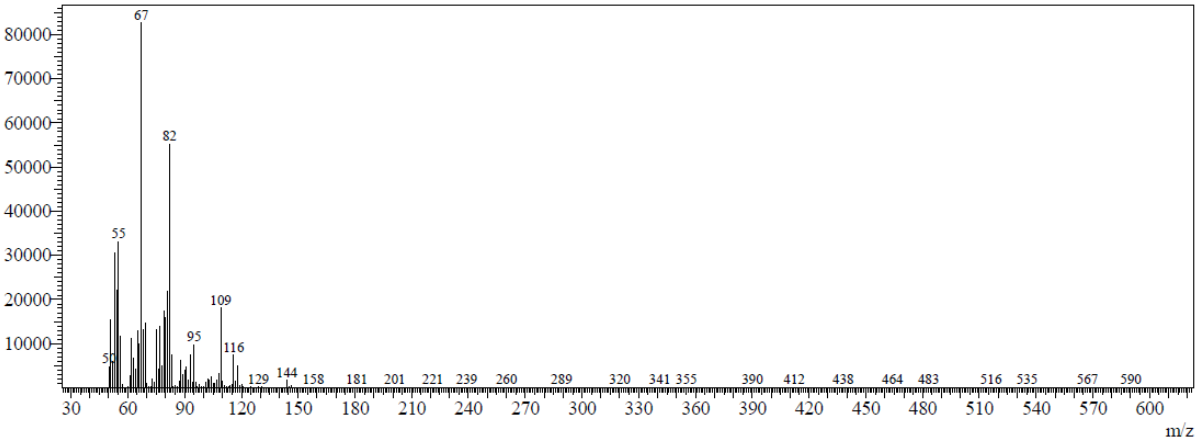

cis-1,2-Dichlorooctene (2c):

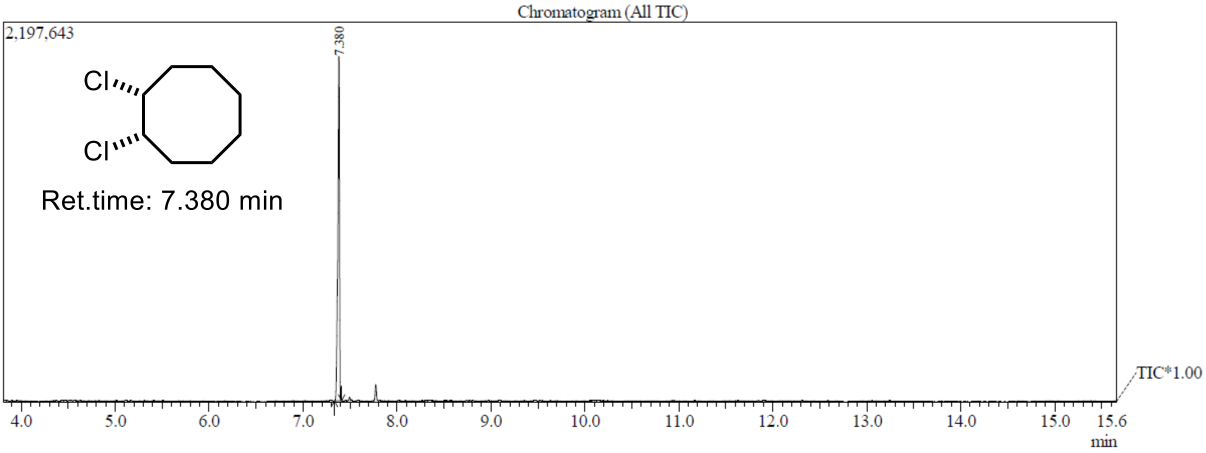

Line#:1 R.Time:7.380(Scan#:1072)
MassPeaks:511
RawMode:Single 7.380(1072) BasePeak:67.05(286354)
BG Mode:None Group 1 - Event 1 Scan

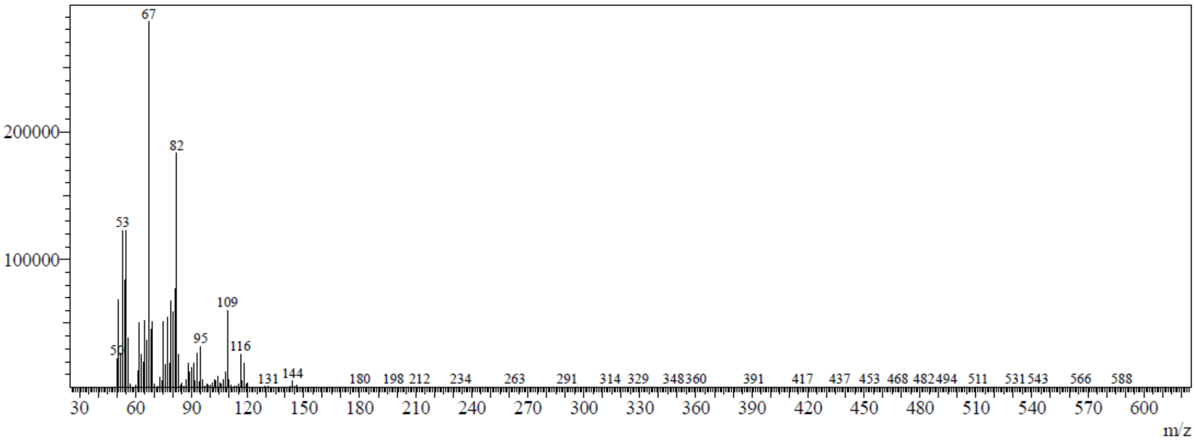

5,6-Dichlorodecane:

***trans*-5,6-Dichlorodecane (*trans*-2h):**

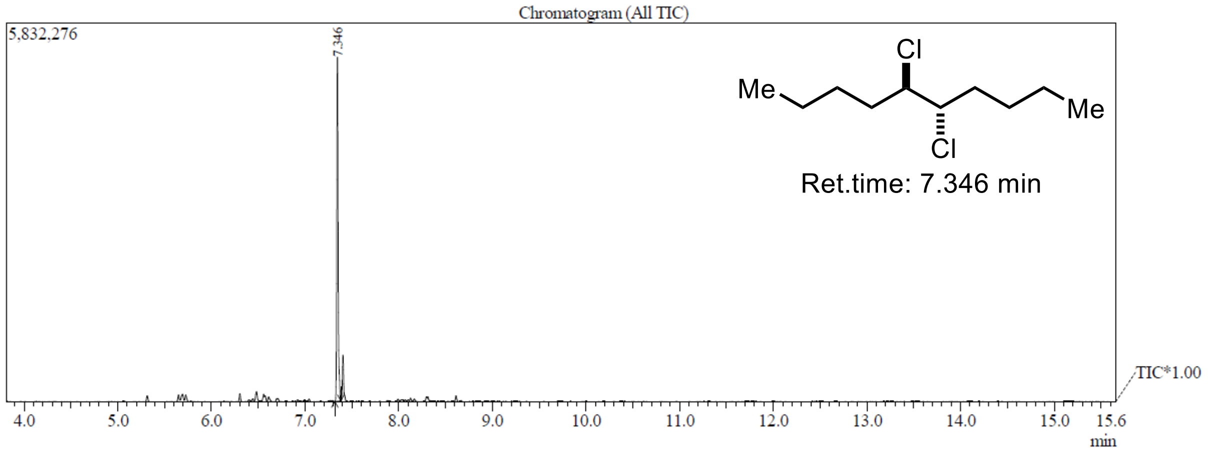

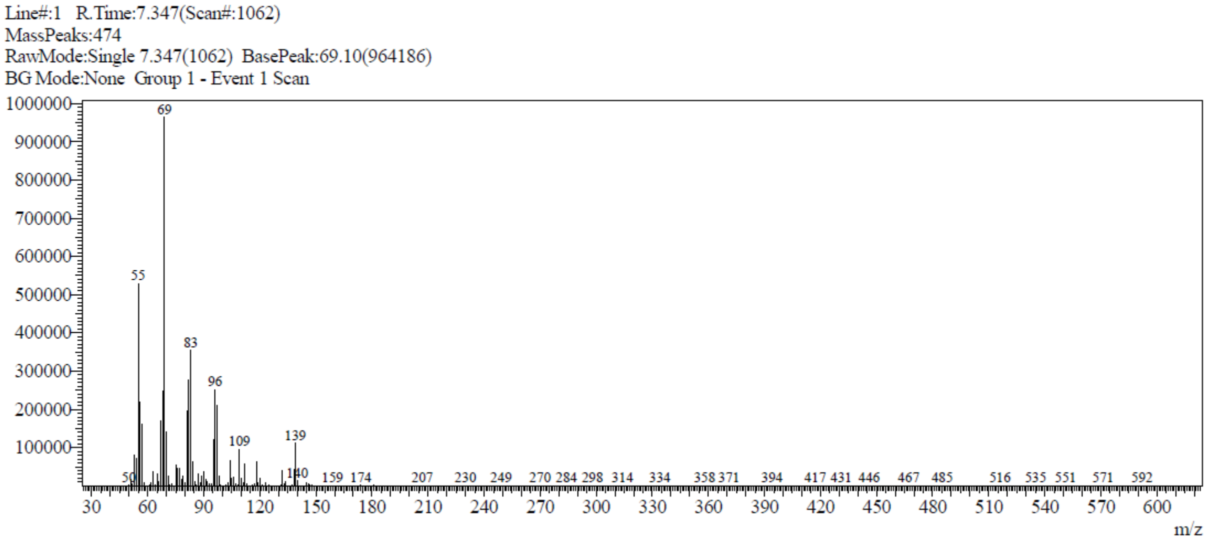

cis-5,6-Dichlorodecane (2h):

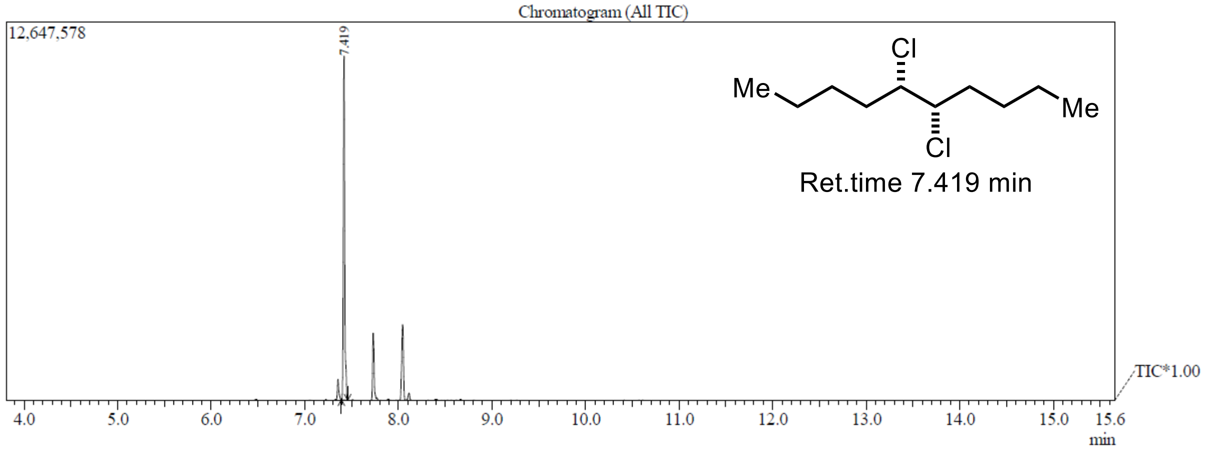

Line#:1 R.Time:7.417(Scan#:1083)
MassPeaks:530
RawMode:Single 7.417(1083) BasePeak:69.10(2120036)
BG Mode:None Group 1 - Event 1 Scan

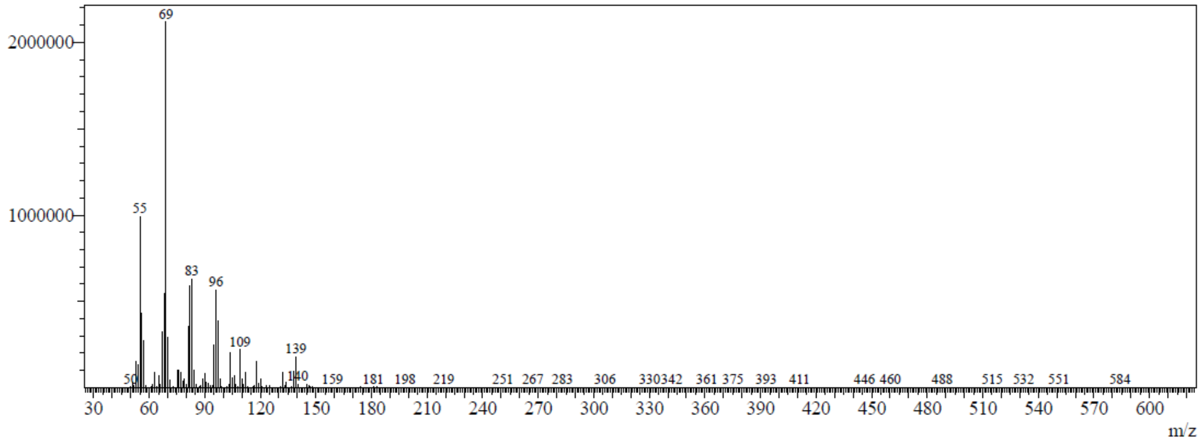

2,3-Dichlorohexan-1-ol:

***trans*-2,3-Dichlorohexan-1-ol (*trans*-2k):**

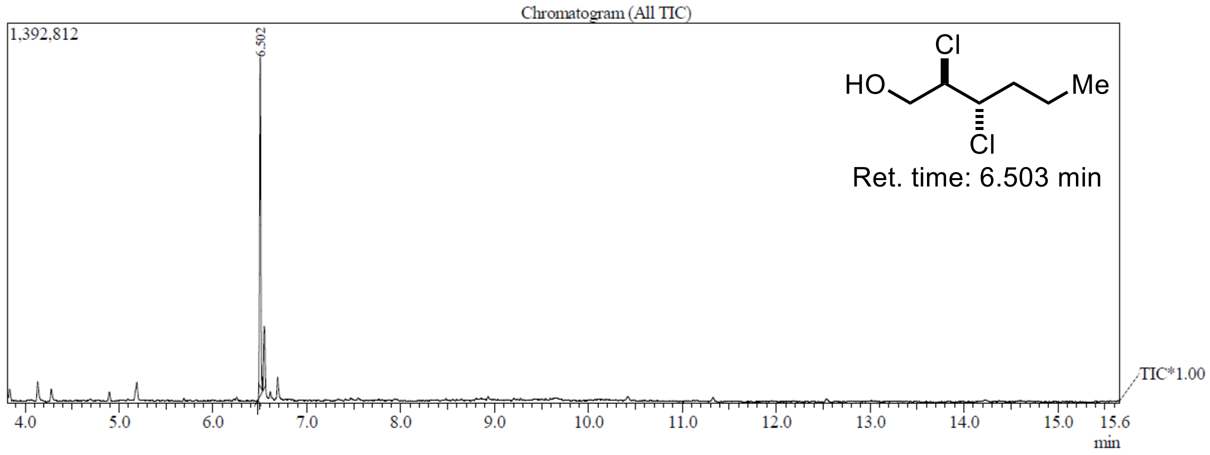

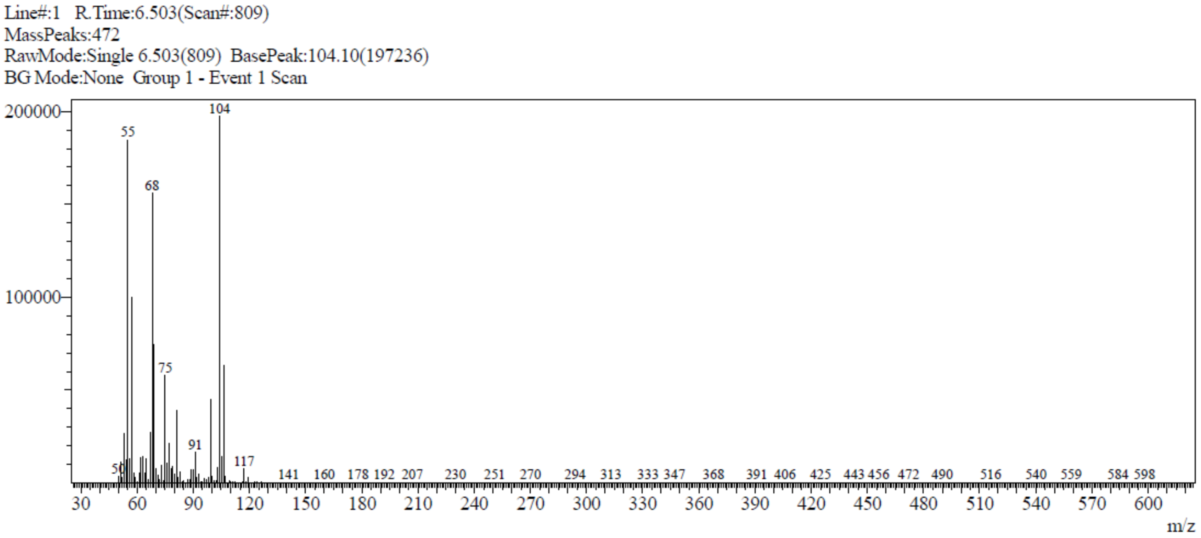

cis-2,3-Dichlorohexan-1-ol (2k):

C:\GCMSsolution\Data\Project1\JUST-1353-F2-7-12_6.qgd

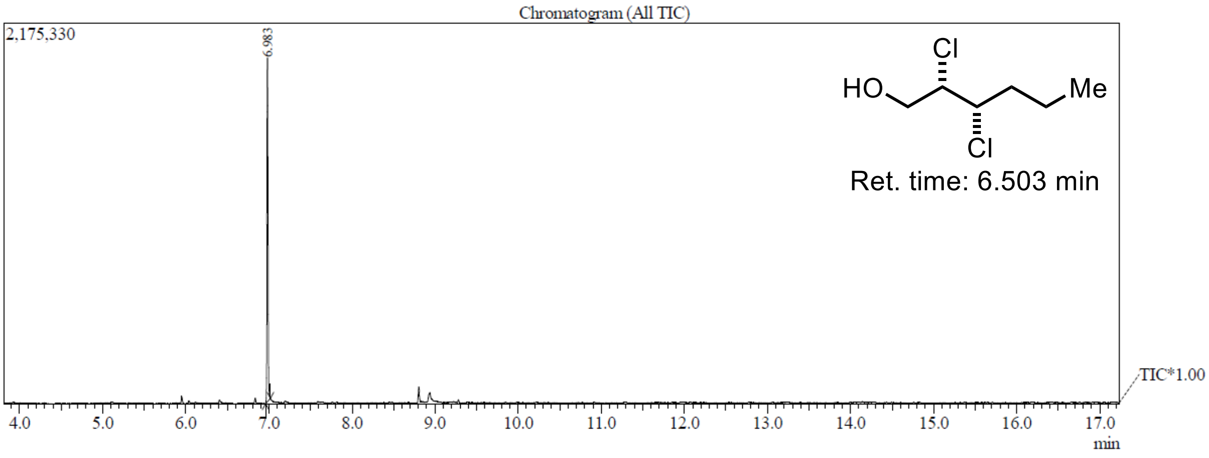

Line#:1 R.Time:6.983(Scan#:953)
MassPeaks:514
RawMode:Single 6.983(953) BasePeak:104.10(346623)
BG Mode:None Group 1 - Event 1 Scan

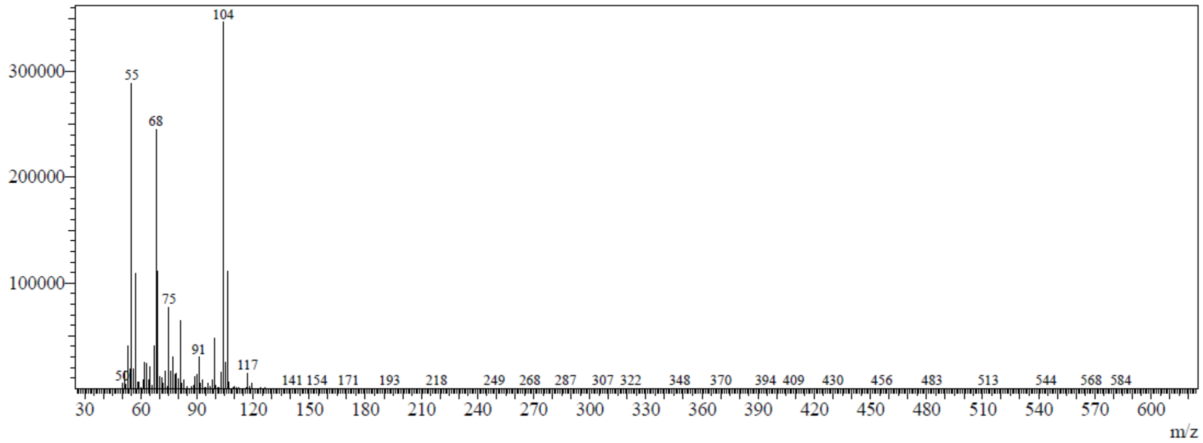

***trans*-2,3-Dichloro-3-phenylpropan-1-ol (*trans*-2l):**

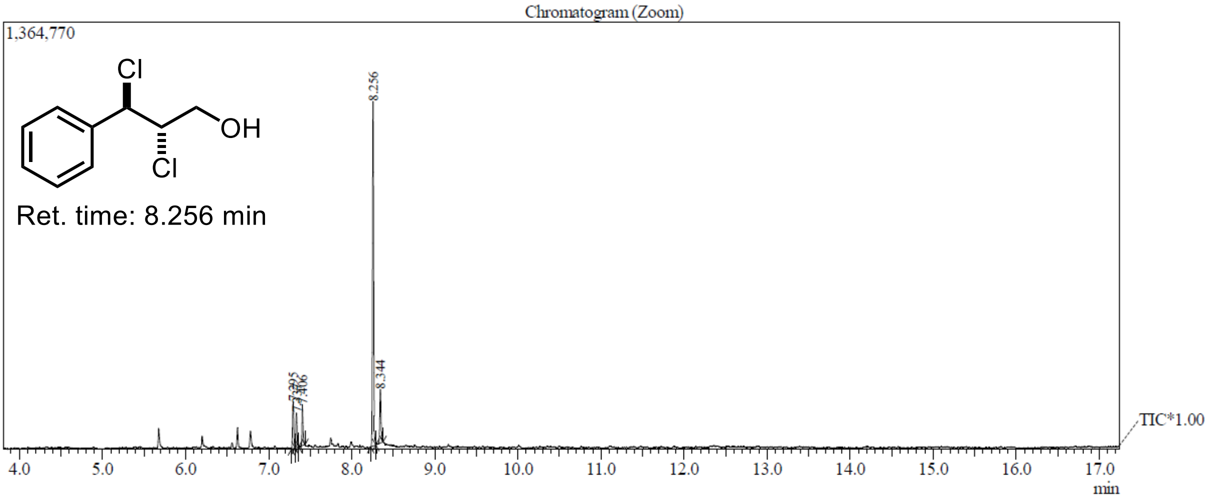

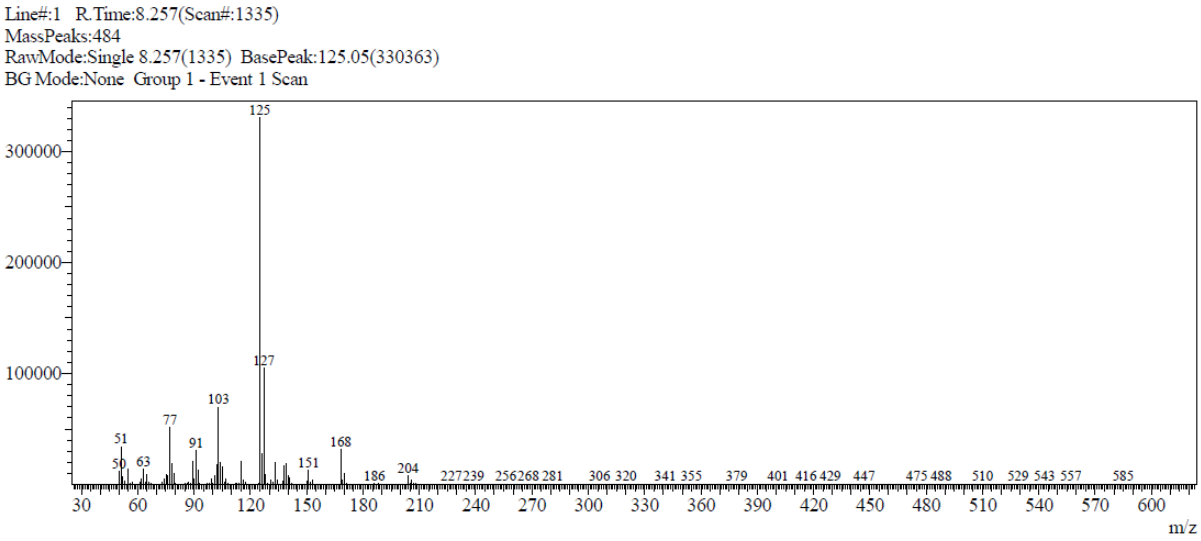

cis-2,3-Dichloro-3-phenylpropan-1-ol (2l):

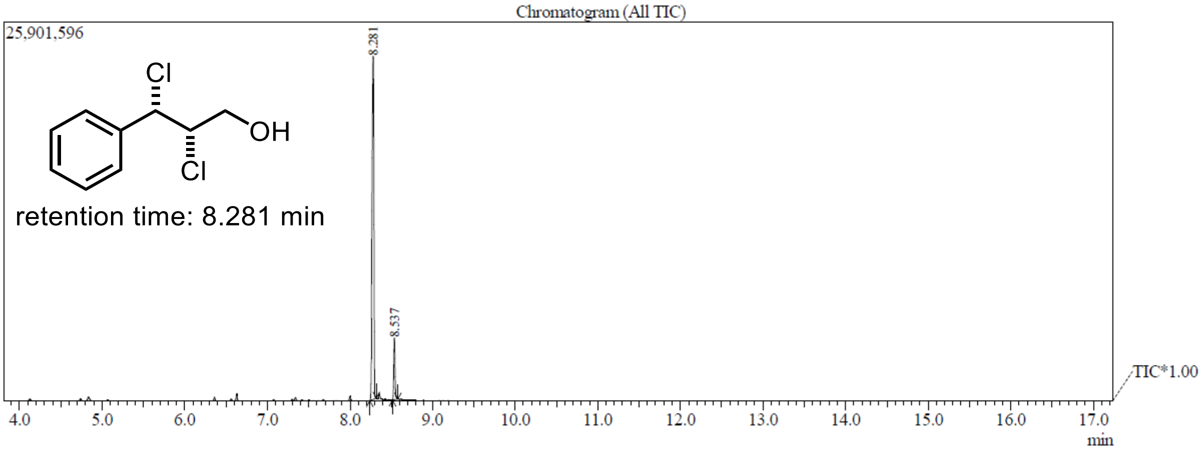

Line#:1 R.Time:8.290(Scan#:1345)
MassPeaks:511
RawMode:Single 8.290(1345) BasePeak:125.10(3708150)
BG Mode:None Group 1 - Event 1 Scan

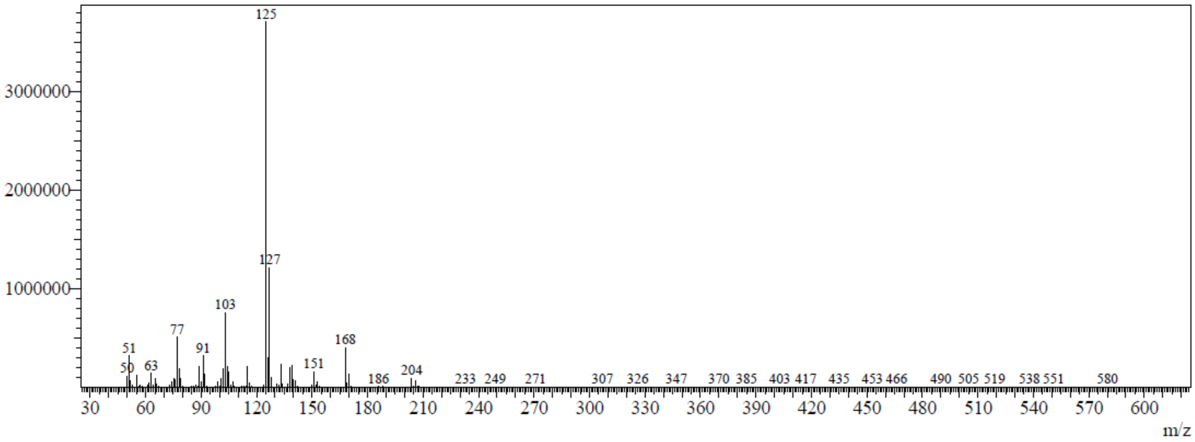

trans-2,3-Dichloro-1,3-diphenylpropan-1-one (*trans*-2n):

C:\GCMSsolution\Data\Project1\JUST-1358_1.qgd

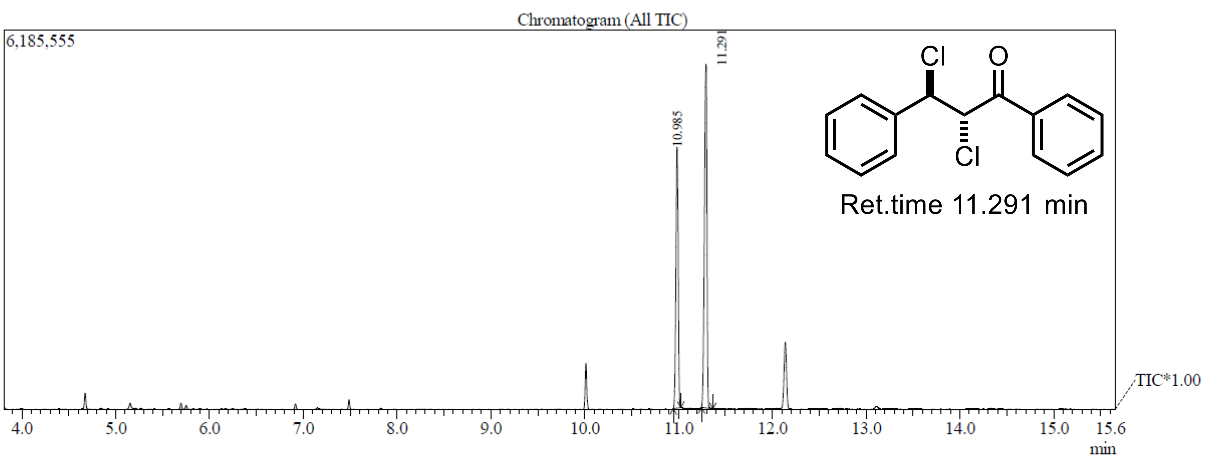

Line#:1 R.Time:11.307(Scan#:2250)
MassPeaks:531
RawMode:Single 11.307(2250) BasePeak:105.10(378790)
BG Mode:None Group 1 - Event 1 Scan

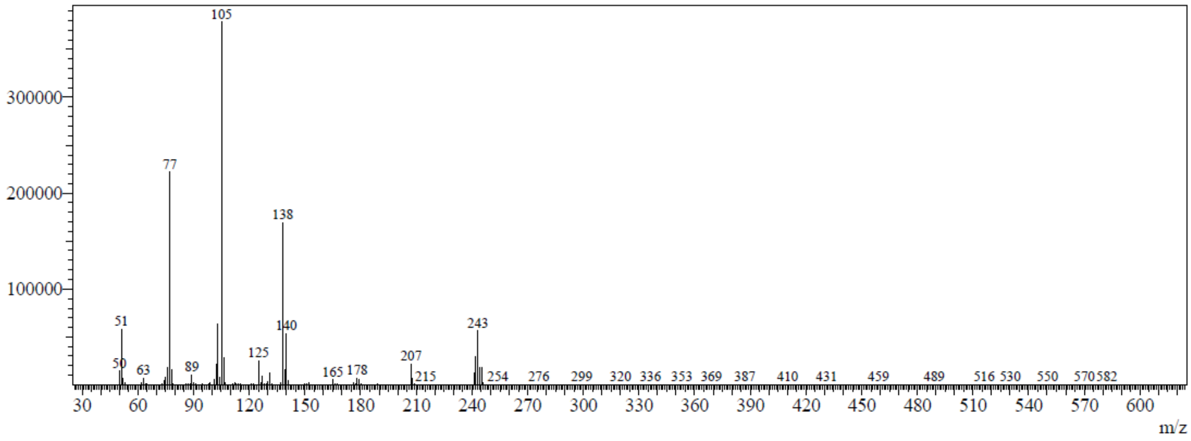

cis-2,3-Dichloro-1,3-diphenylpropan-1-one (2n):

C:\GCMSsolution\Data\Project1\JUST-1327-F2b_7.qgd

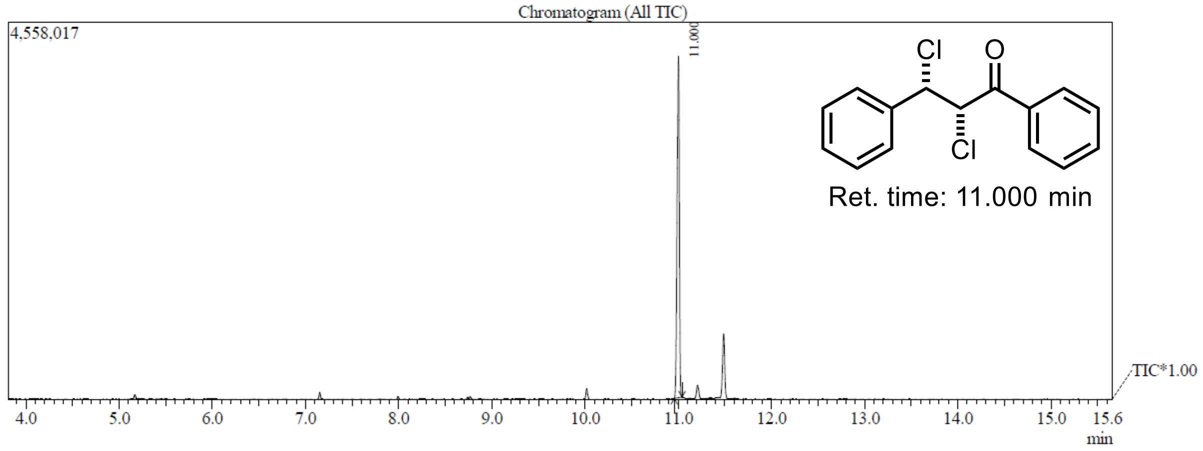

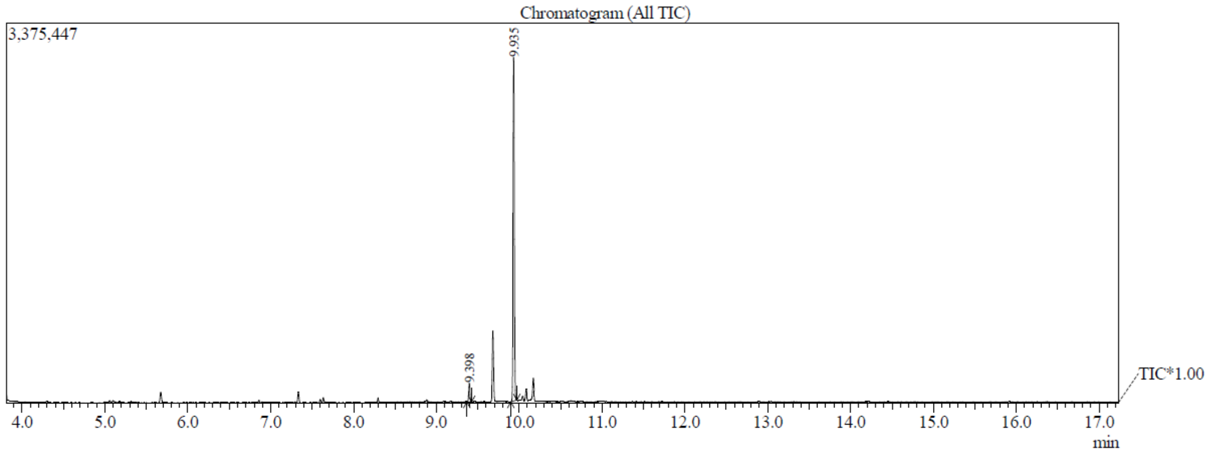

Line#:1 R.Time:9.933(Scan#:1838)
MassPeaks:535
RawMode:Single 9.933(1838) BasePeak:105.10(785545)
BG Mode:None Group 1 - Event 1 Scan

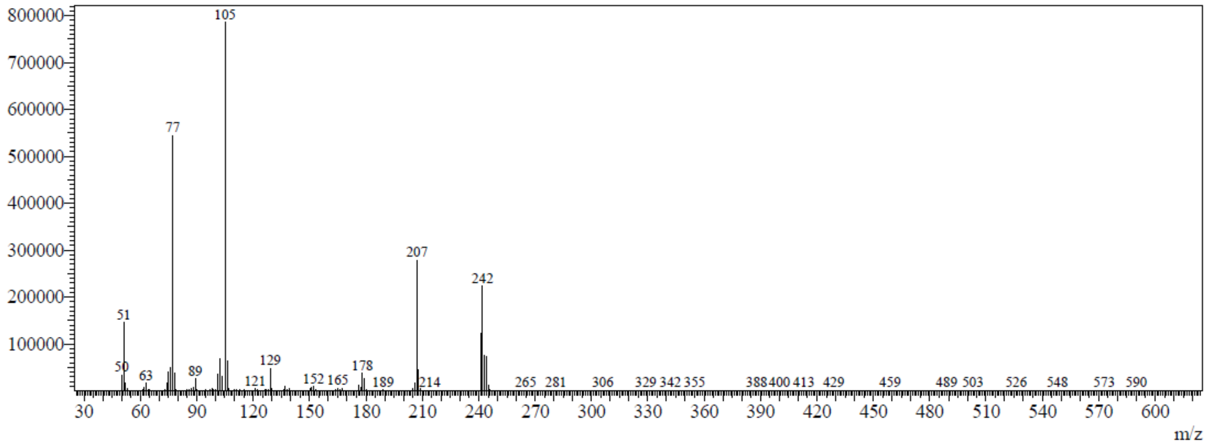

5. NMR spectra of the synthesised products

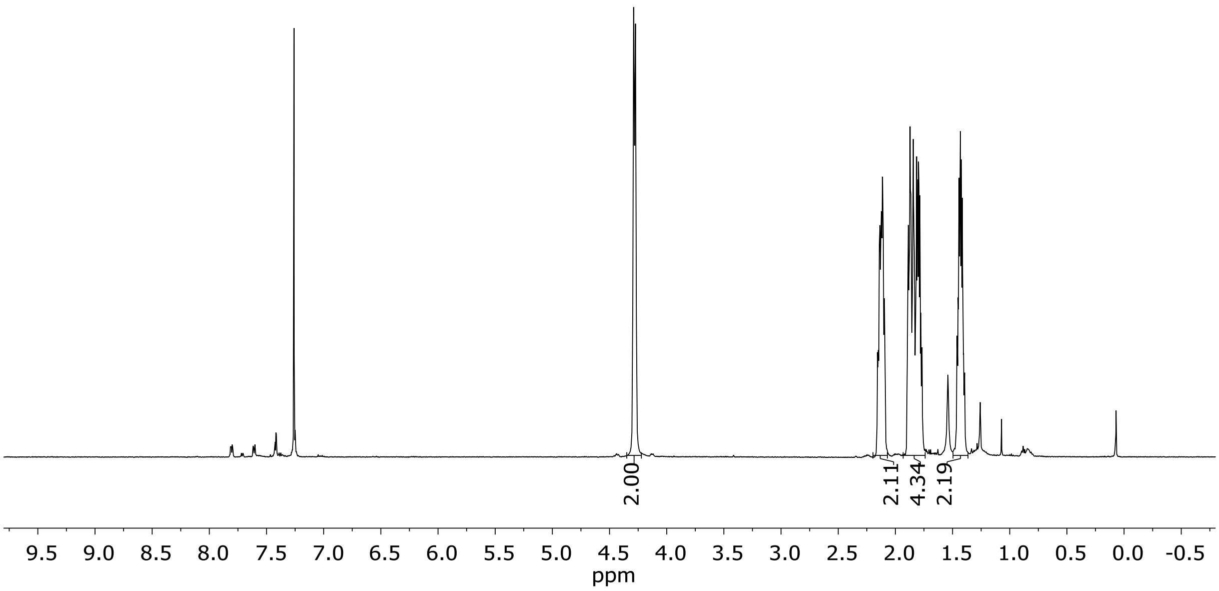

^{1}H NMR (500 MHz, $CDCl_3$) of compound **2b**.

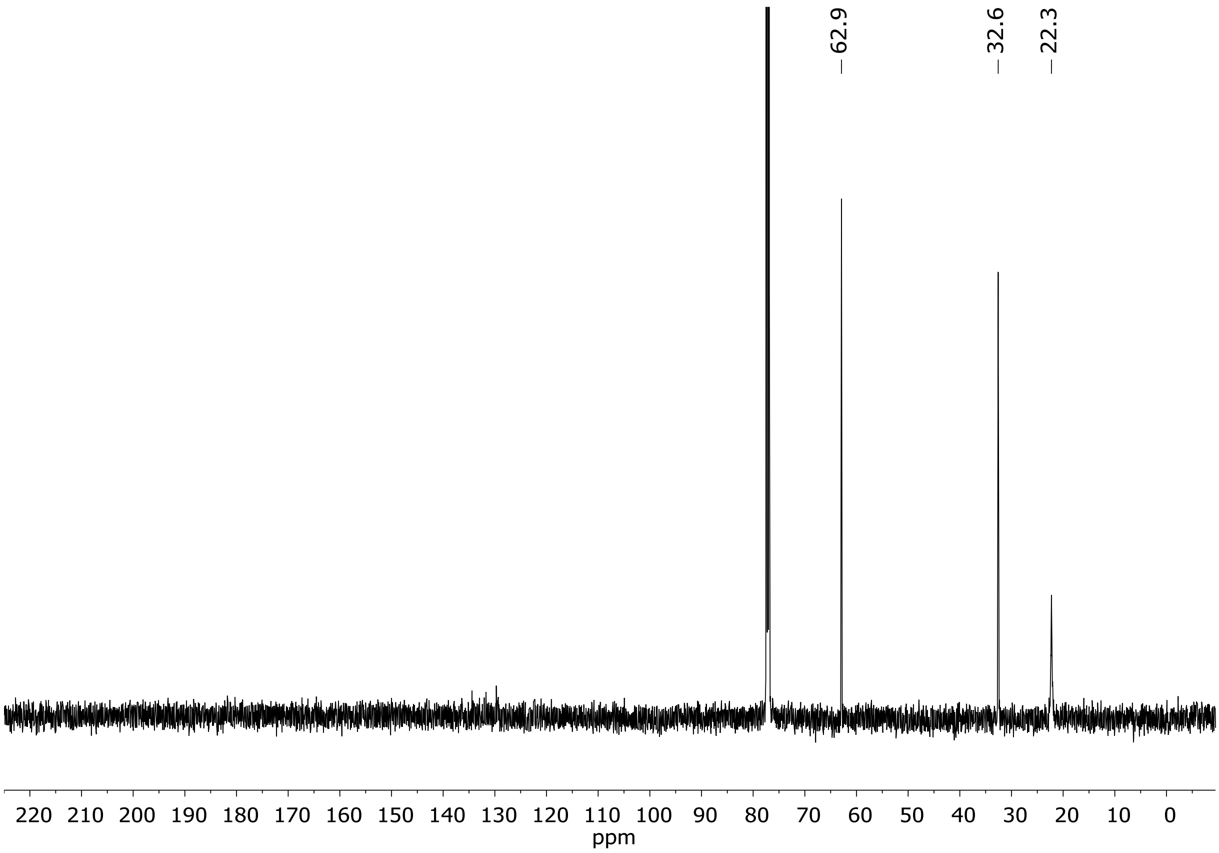

^{13}C NMR (125 MHz, $CDCl_3$) of compound **2b**.

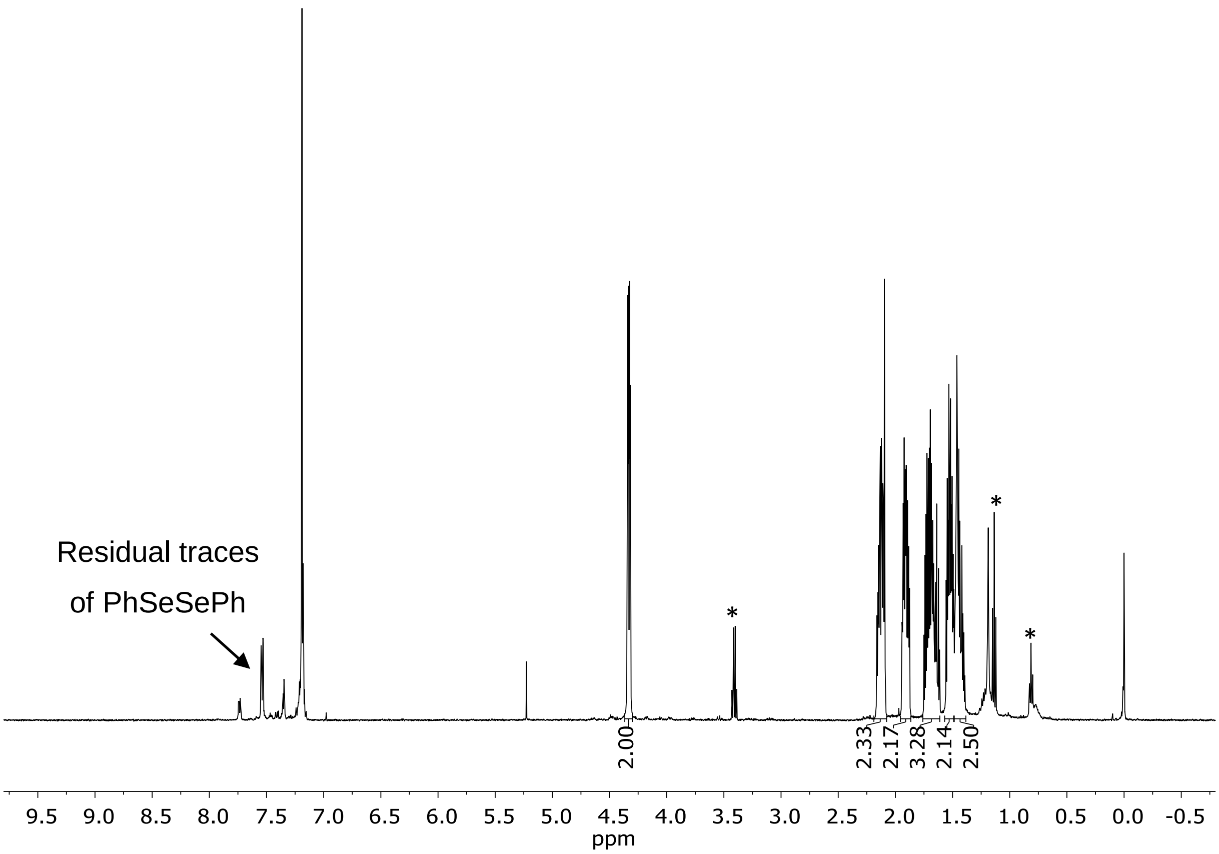

^1H NMR (500 MHz, $CDCl_3$) of compound **2a**. Residual solvent signals are marked.

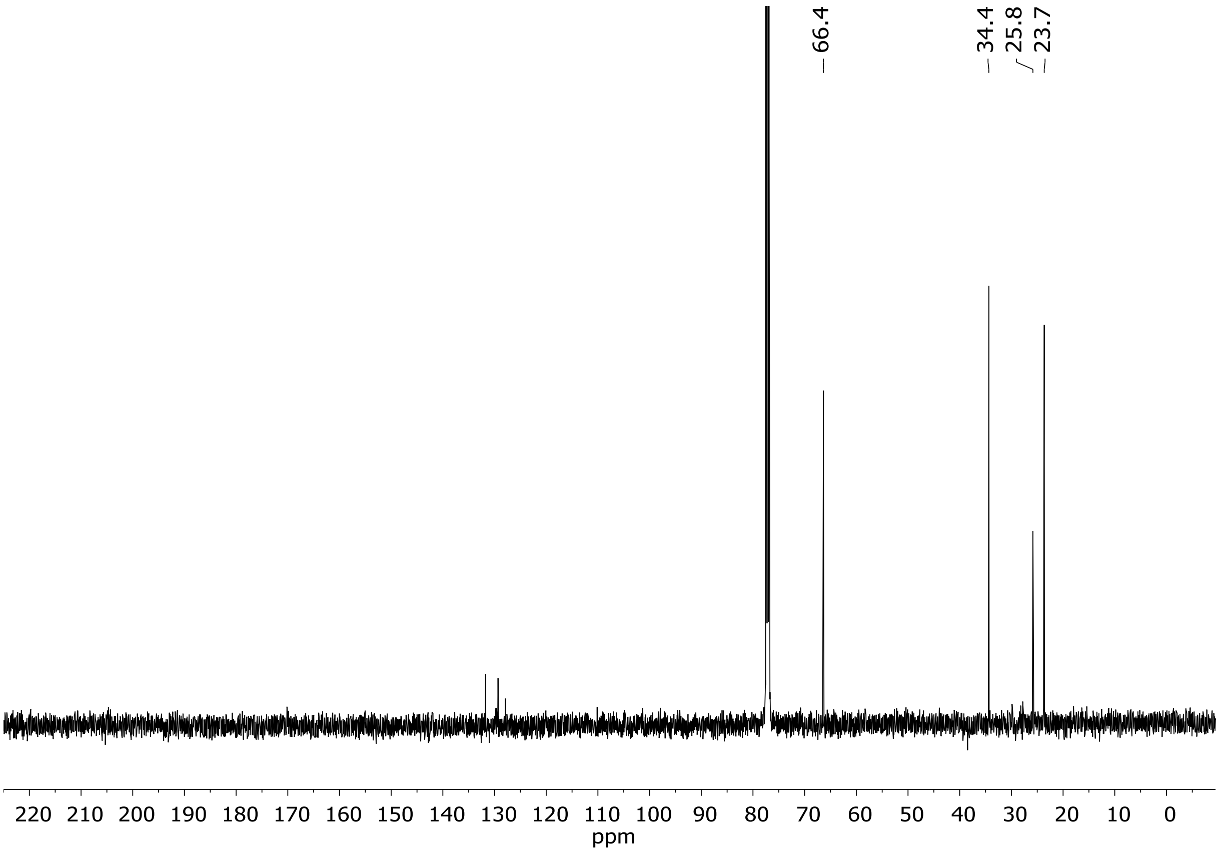

^{13}C NMR (125 MHz, $CDCl_3$) of compound **2a**.

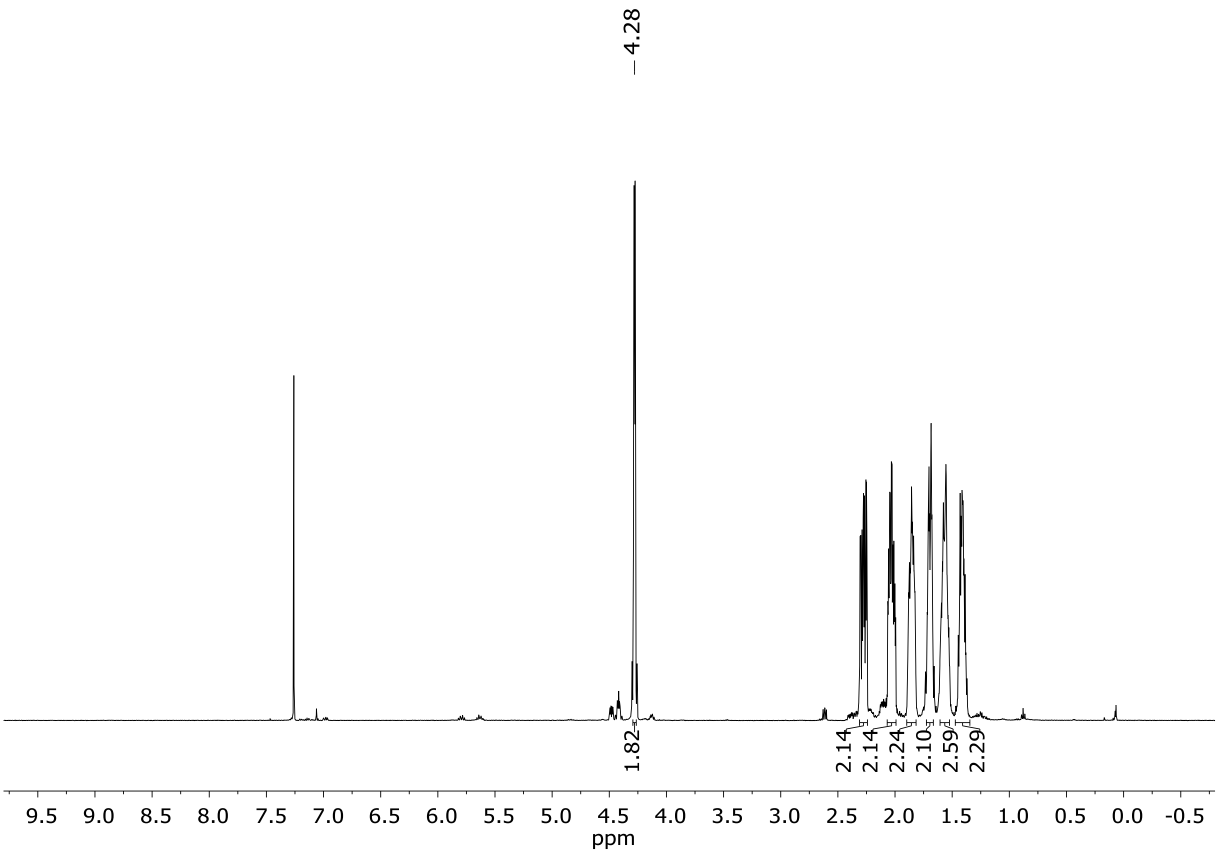

^{1}H NMR (500 MHz, $CDCl_3$) of compound **2c**.

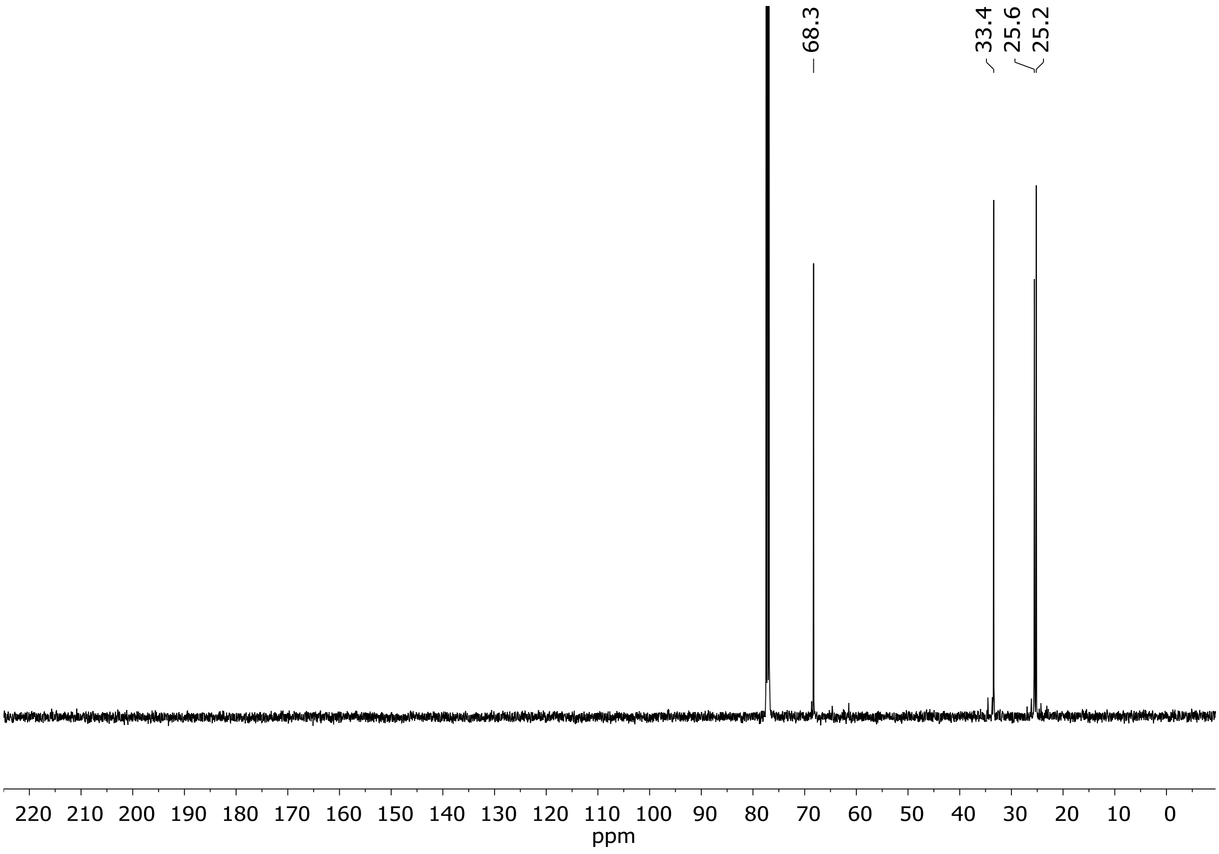

^{13}C NMR (125 MHz, $CDCl_3$) of compound **2c**.

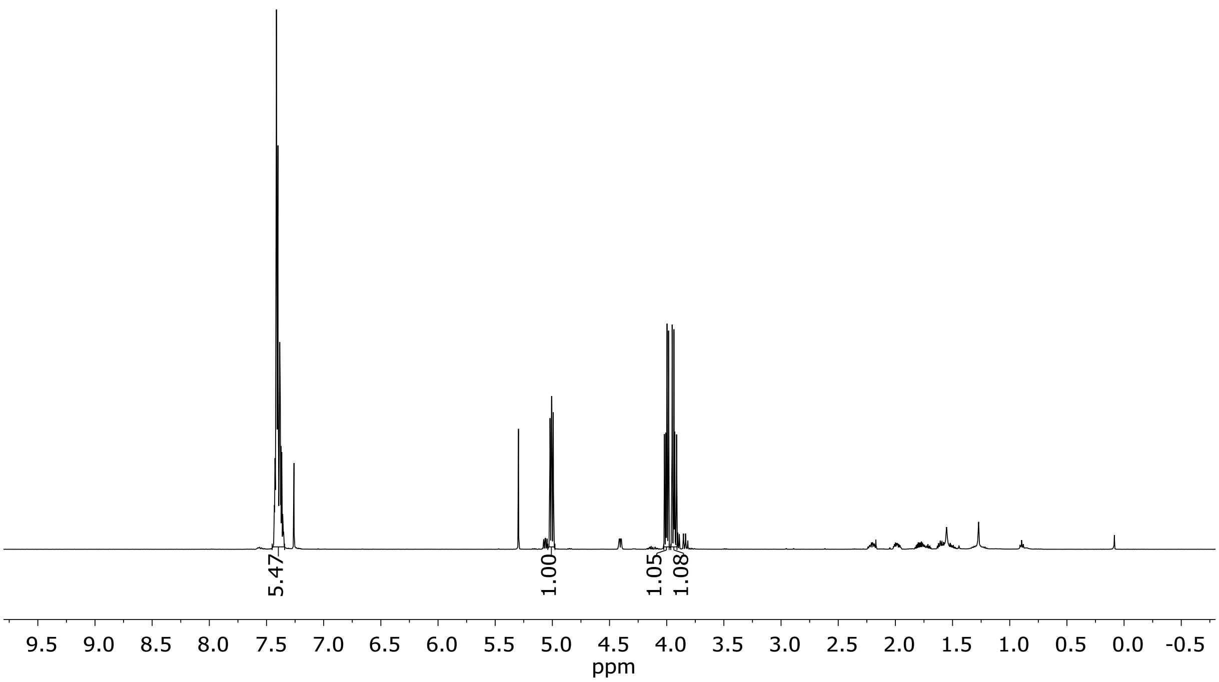

^{1}H NMR (500 MHz, $CDCl_3$) of compound **2d**.

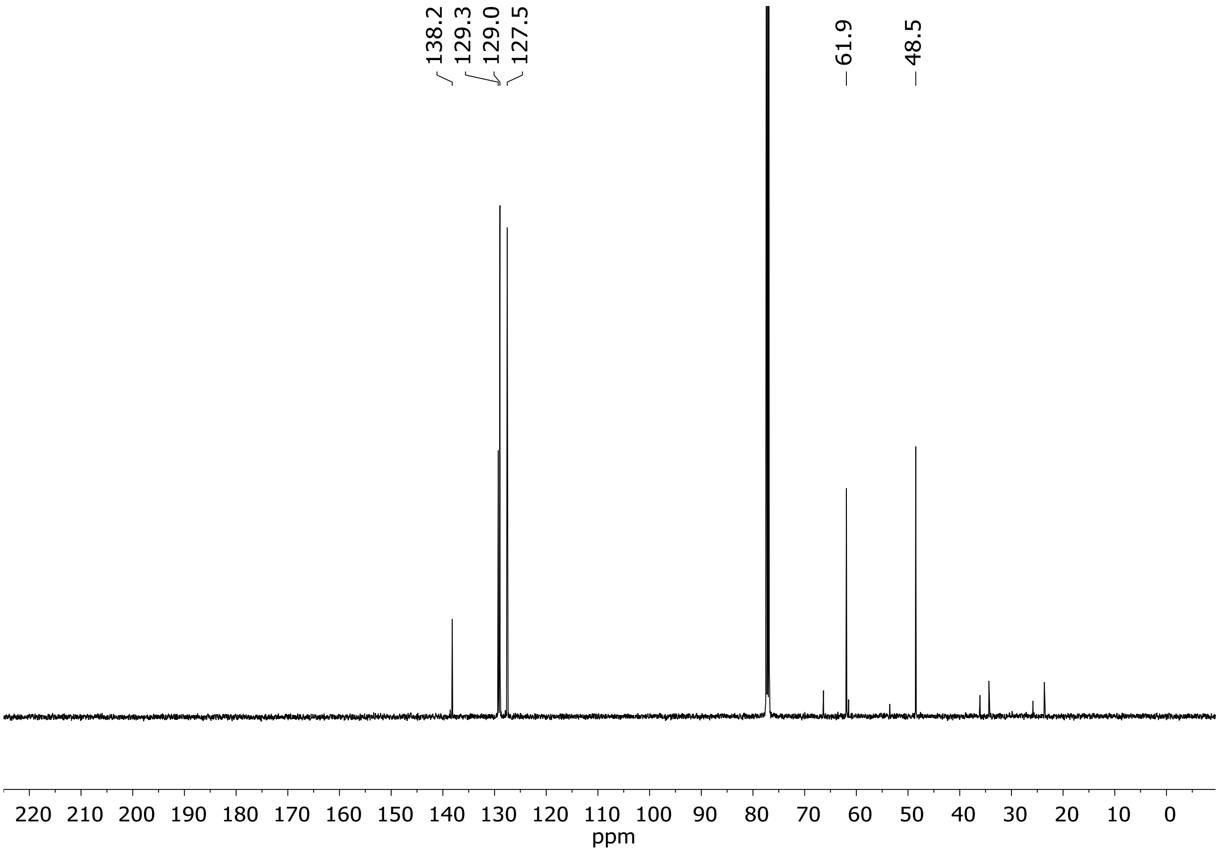

^{13}C NMR (125 MHz, $CDCl_3$) of compound **2d**.

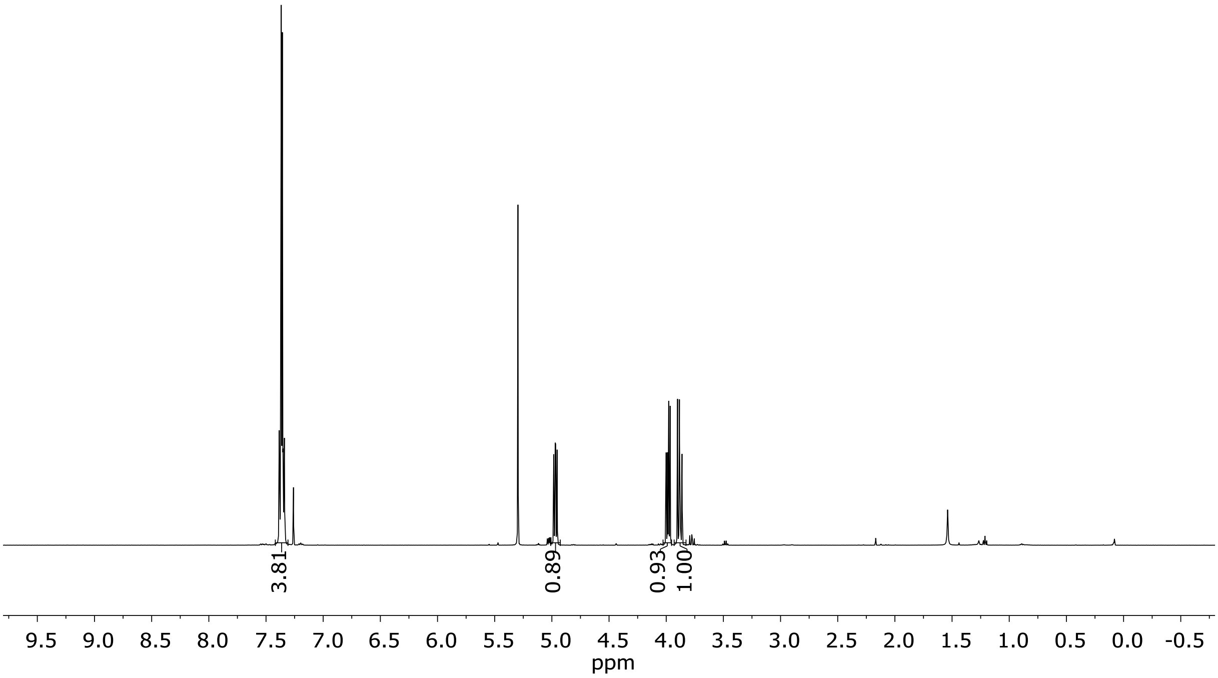

^{1}H NMR (500 MHz, $CDCl_3$) of compound **2e**.

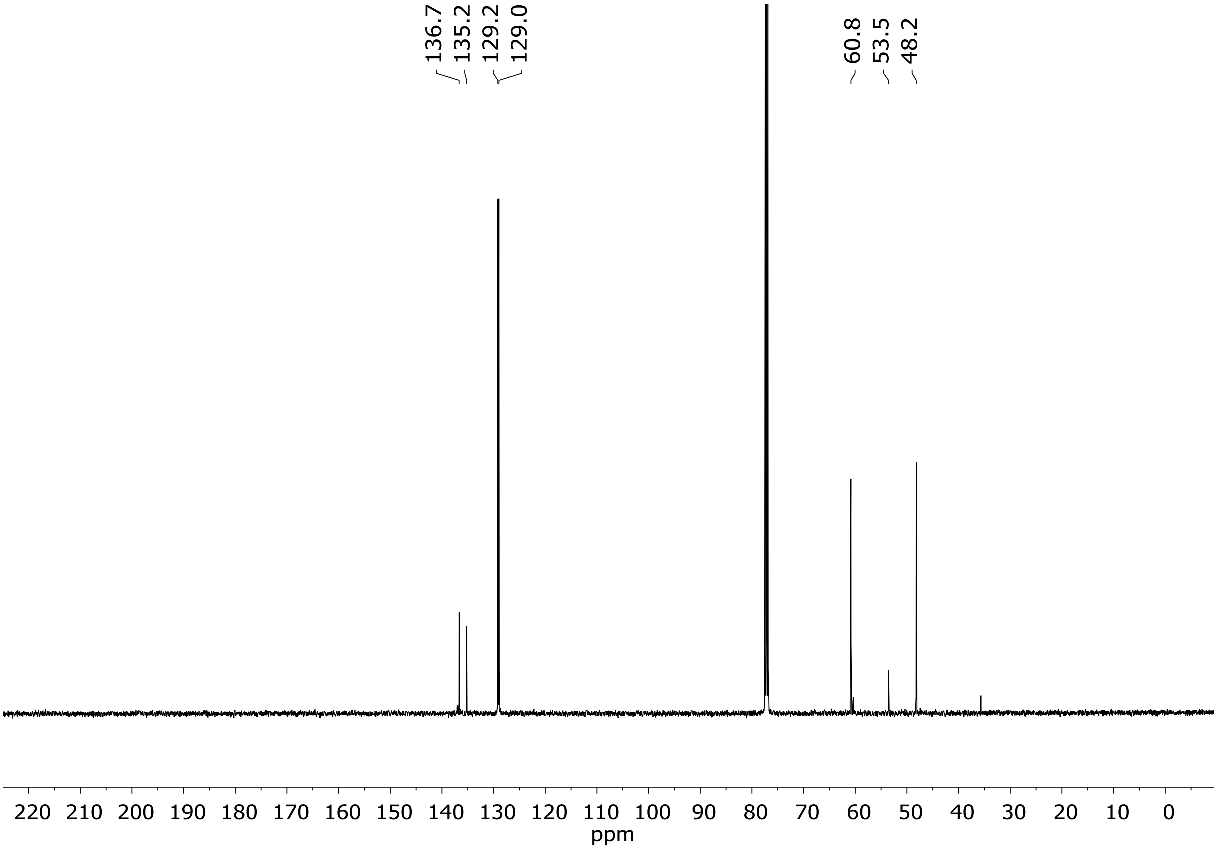

^{13}C NMR (125 MHz, $CDCl_3$) of compound **2e**.

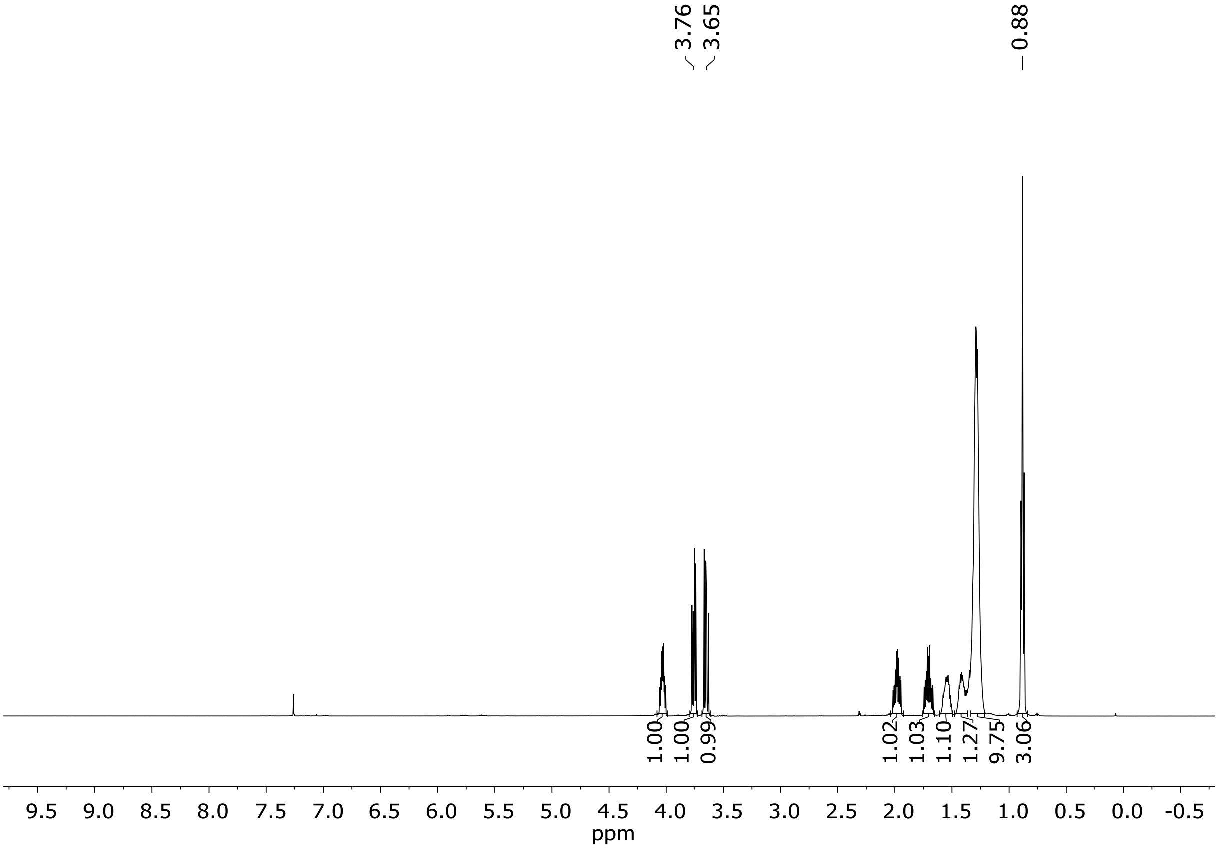

^{1}H NMR (500 MHz, $CDCl_3$) of compound **2f**.

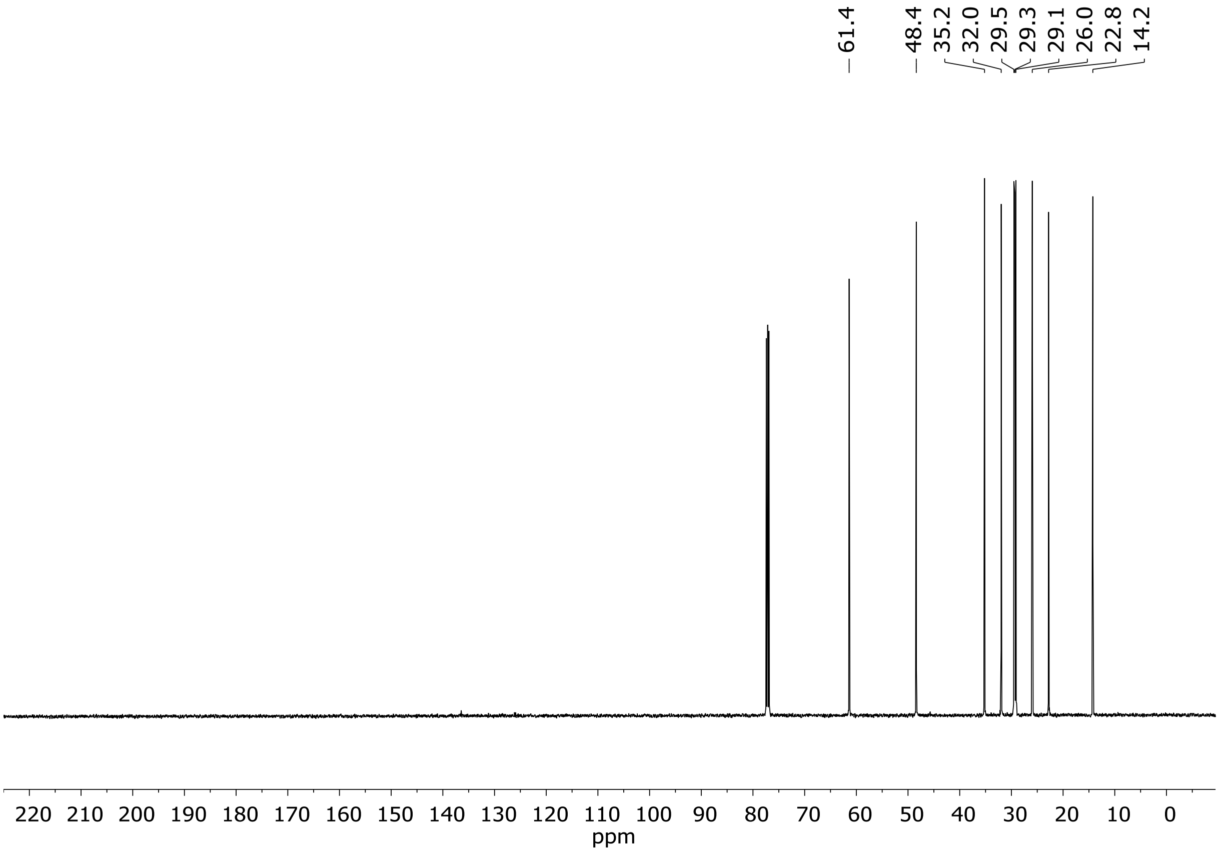

^{13}C NMR (125 MHz, $CDCl_3$) of compound **2f**.

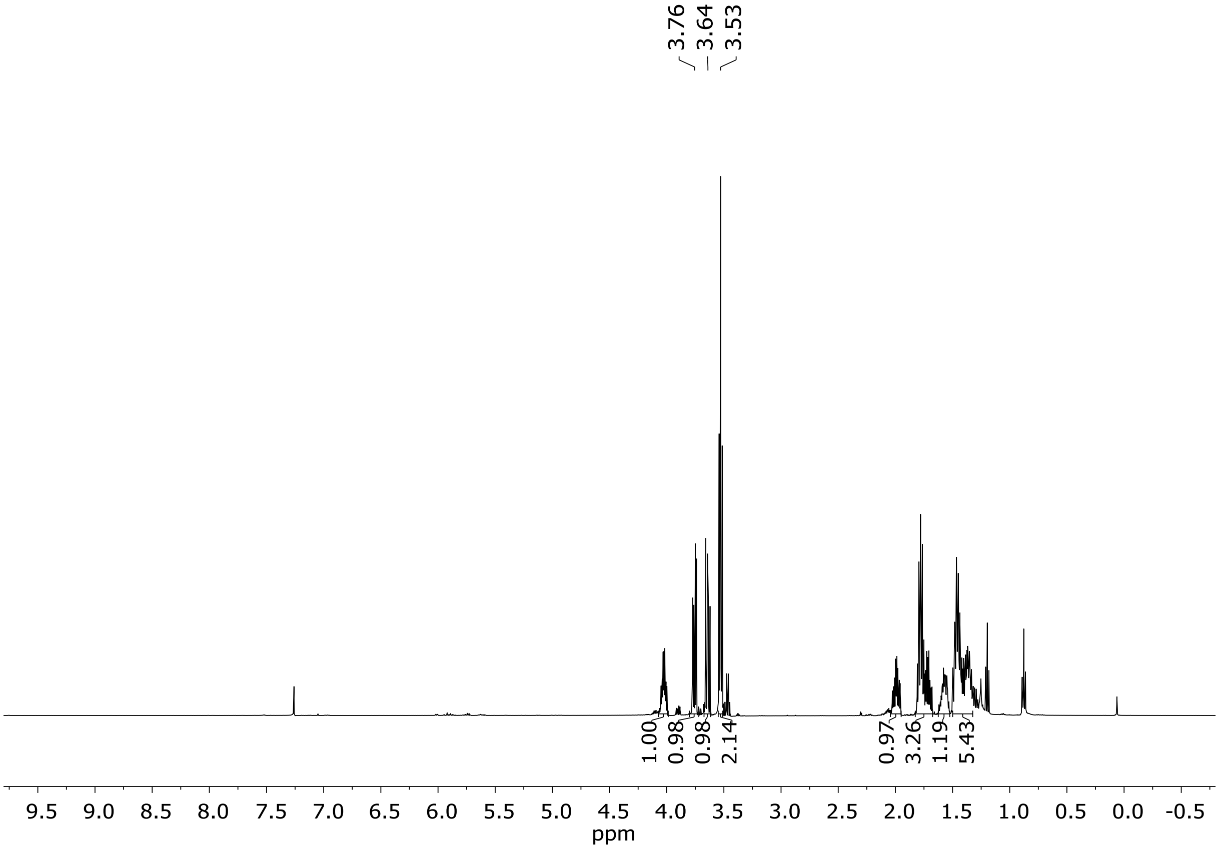

^{1}H NMR (500 MHz, $CDCl_3$) of compound **2g**.

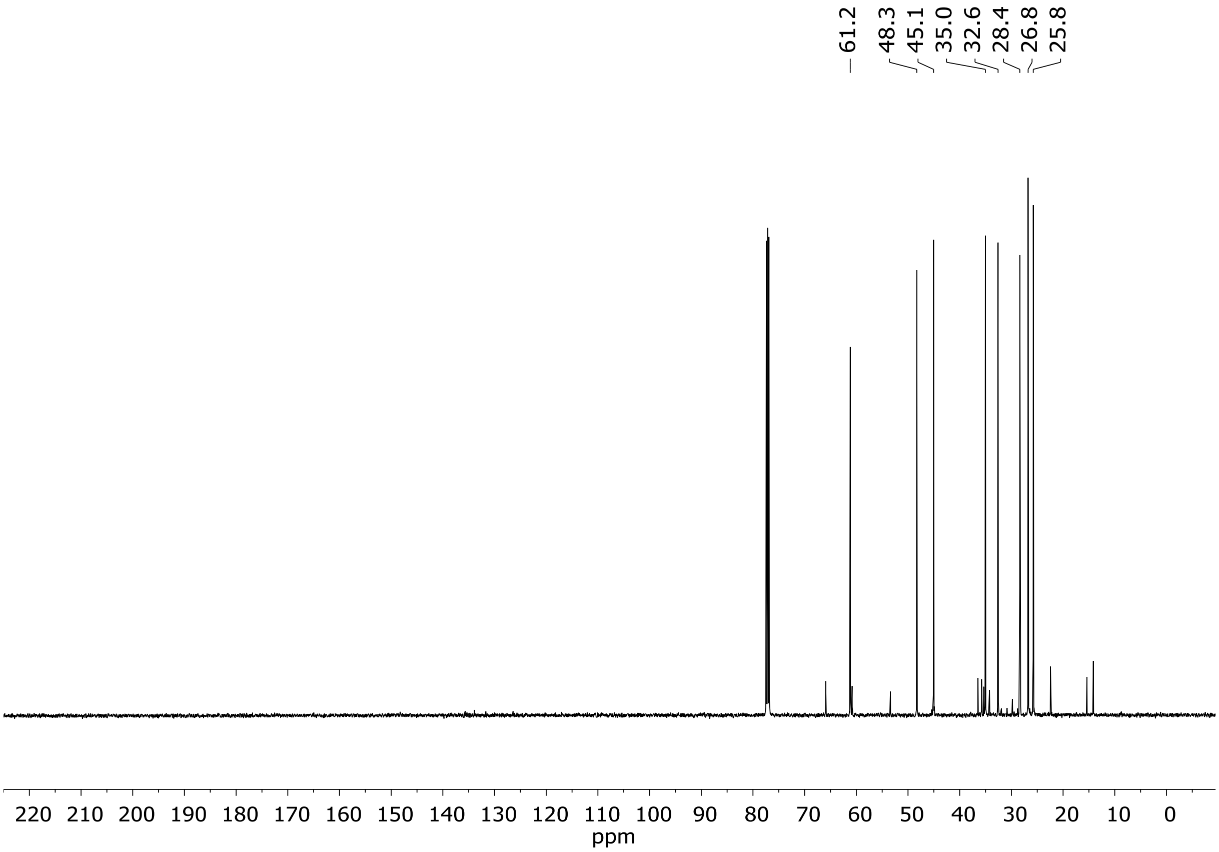

^{13}C NMR (125 MHz, $CDCl_3$) of compound **2g**.

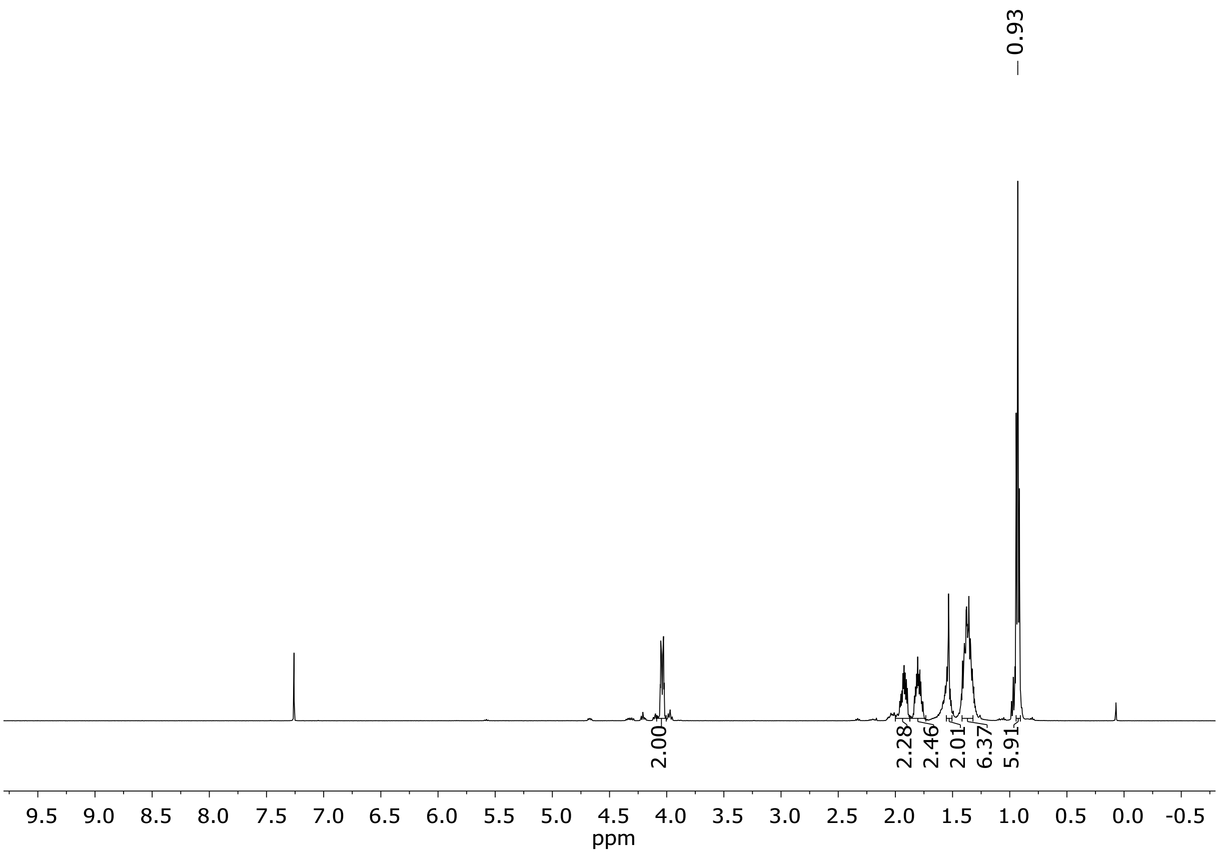

^{1}H NMR (500 MHz, $CDCl_3$) of compound **2h**.

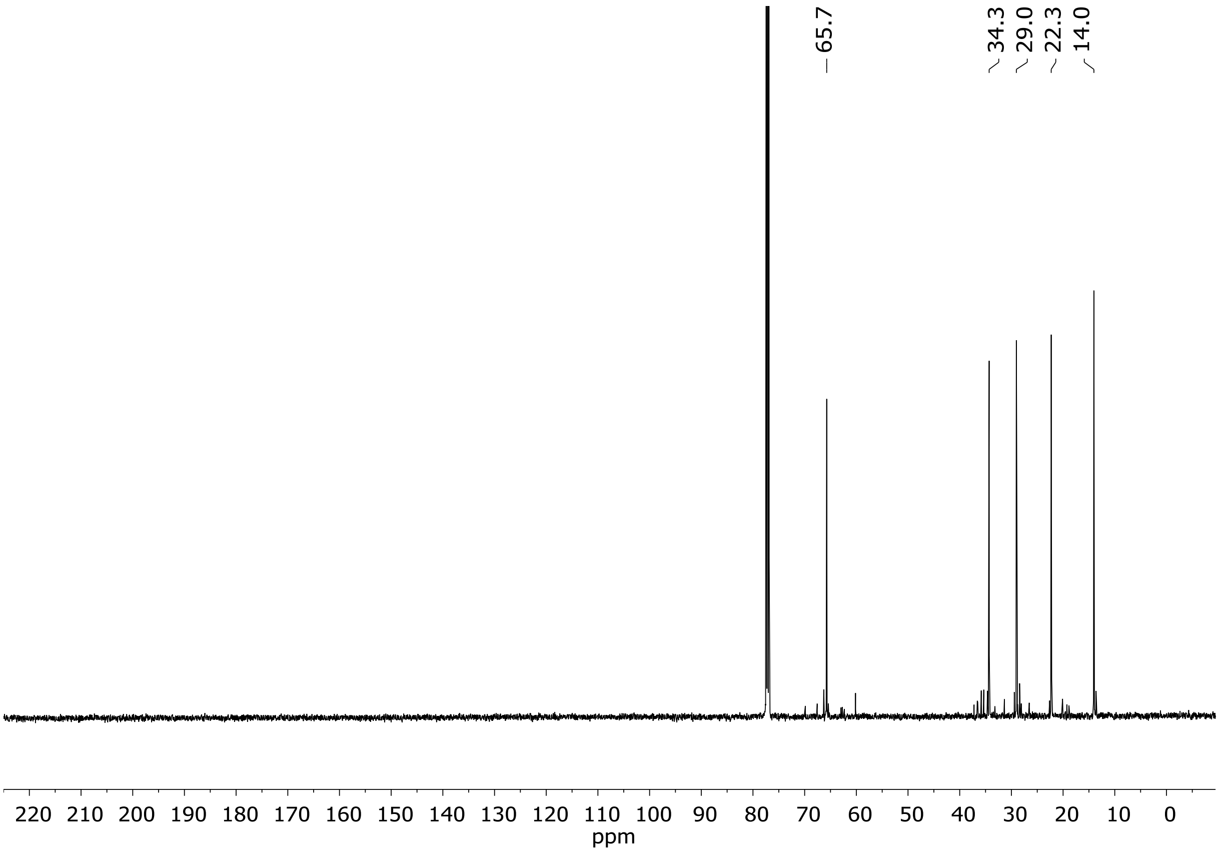

^{13}C NMR (125 MHz, $CDCl_3$) of compound **2h**.

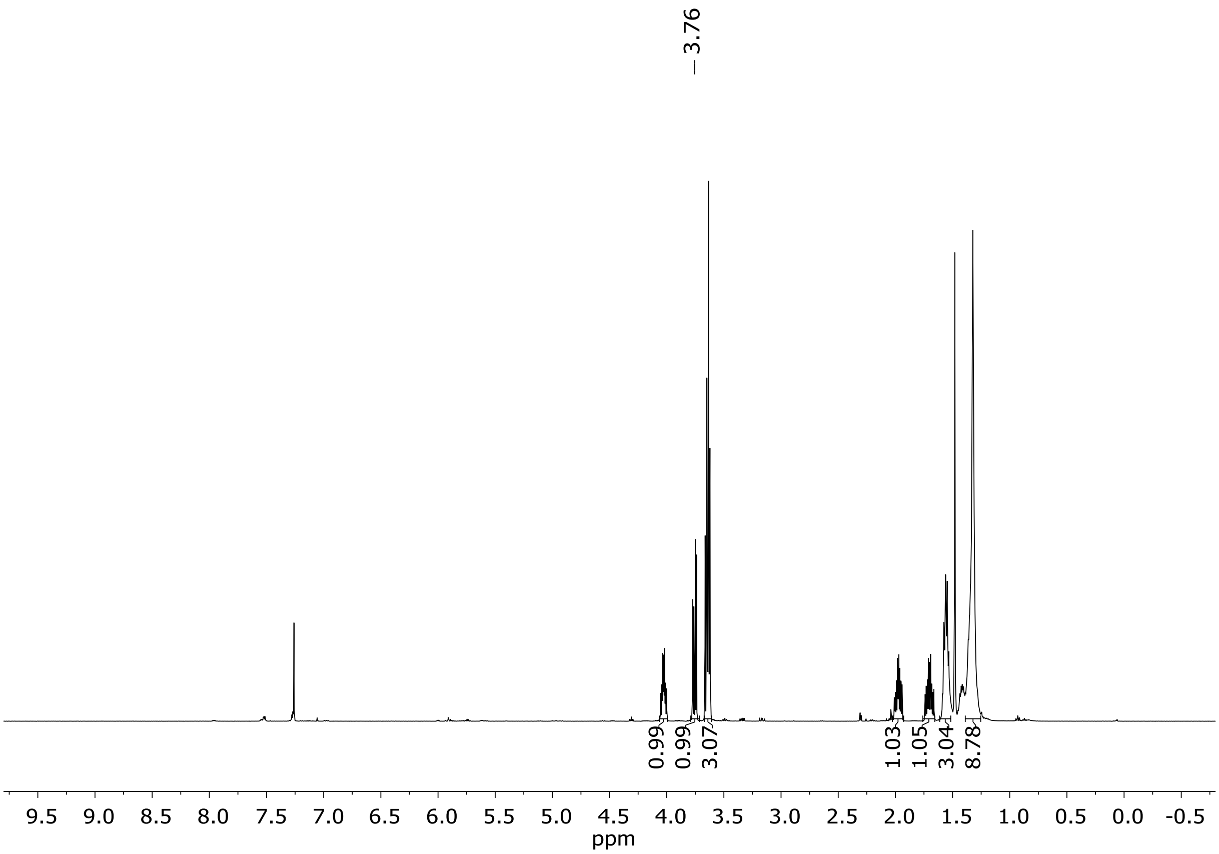

^{1}H NMR (500 MHz, $CDCl_3$) of compound **2i**.

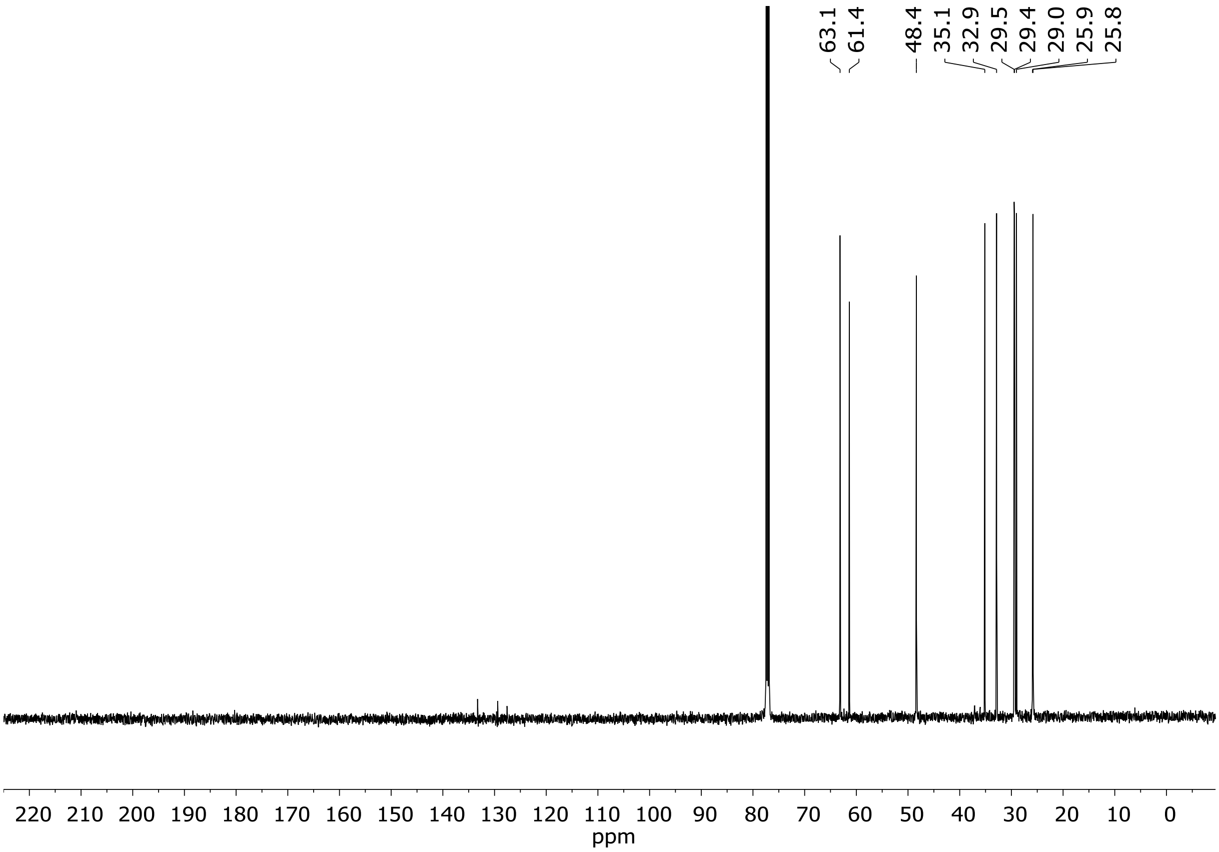

^{13}C NMR (125 MHz, $CDCl_3$) of compound **2i**.

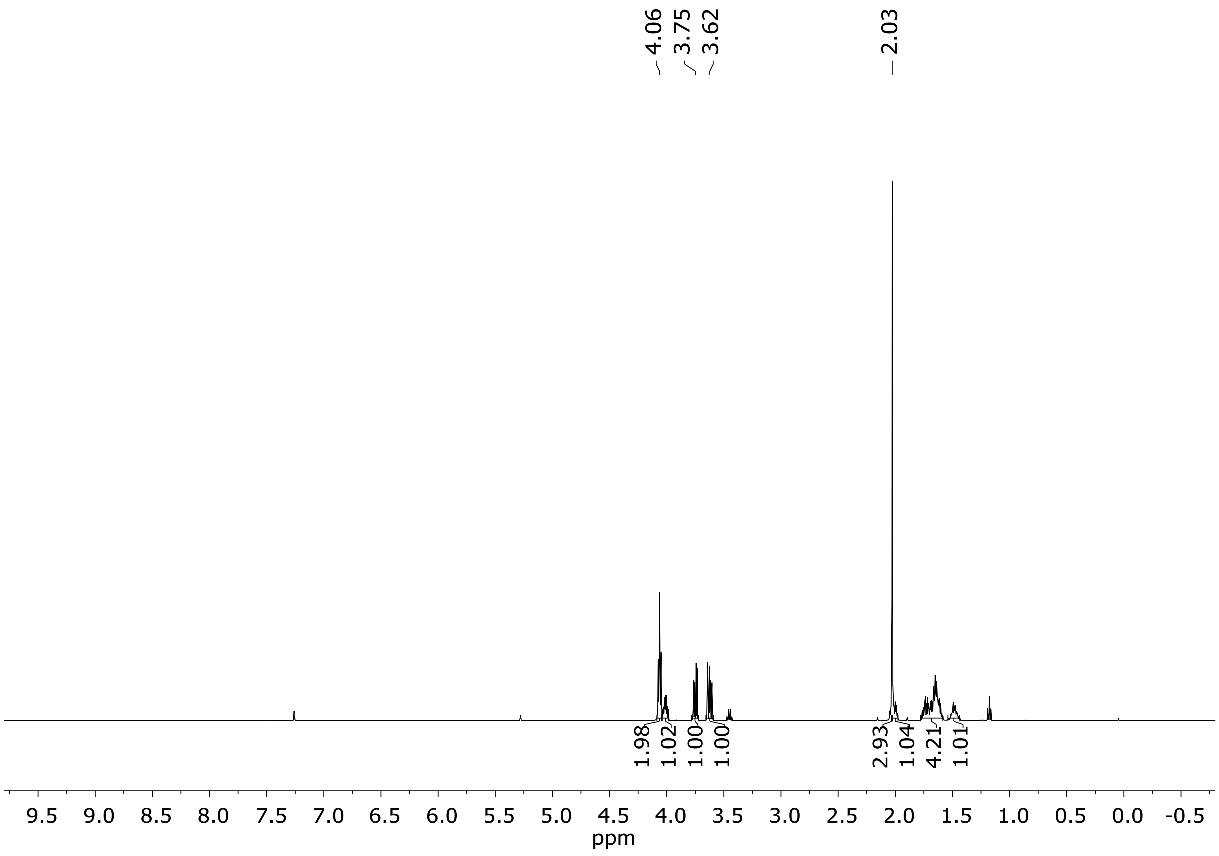

^{1}H NMR (500 MHz, $CDCl_3$) of compound **2j**.

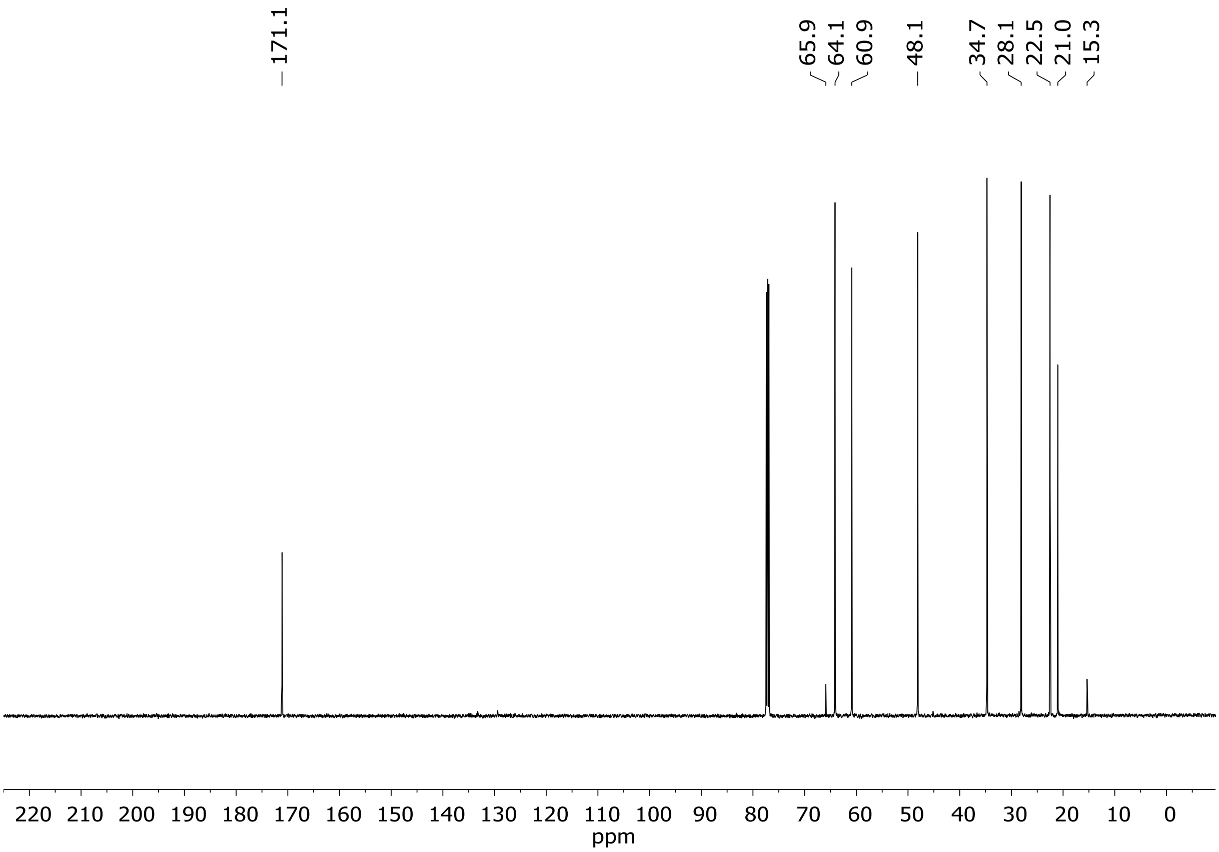

^{13}C NMR (125 MHz, $CDCl_3$) of compound **2j**.

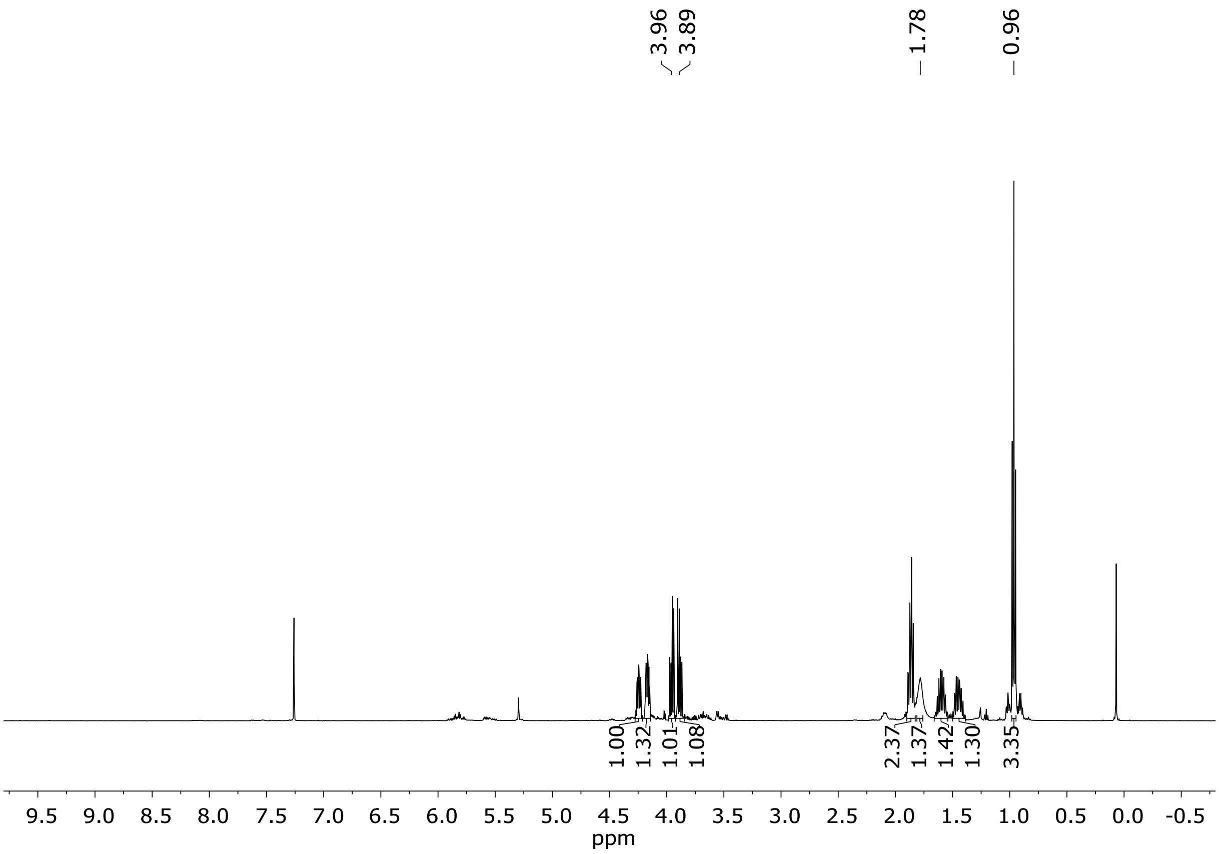

^{1}H NMR (500 MHz, $CDCl_3$) of compound **2k**.

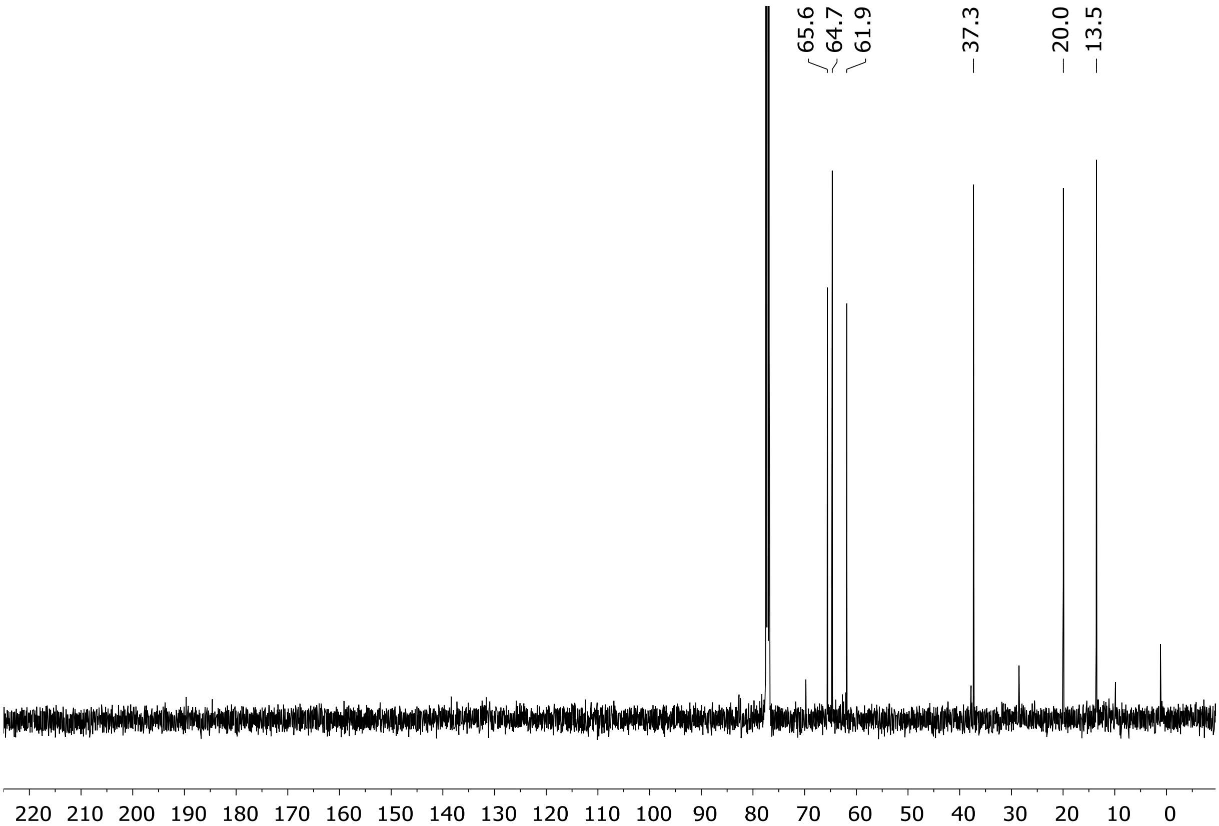

^{13}C NMR (125 MHz, $CDCl_3$) of compound **2k**.

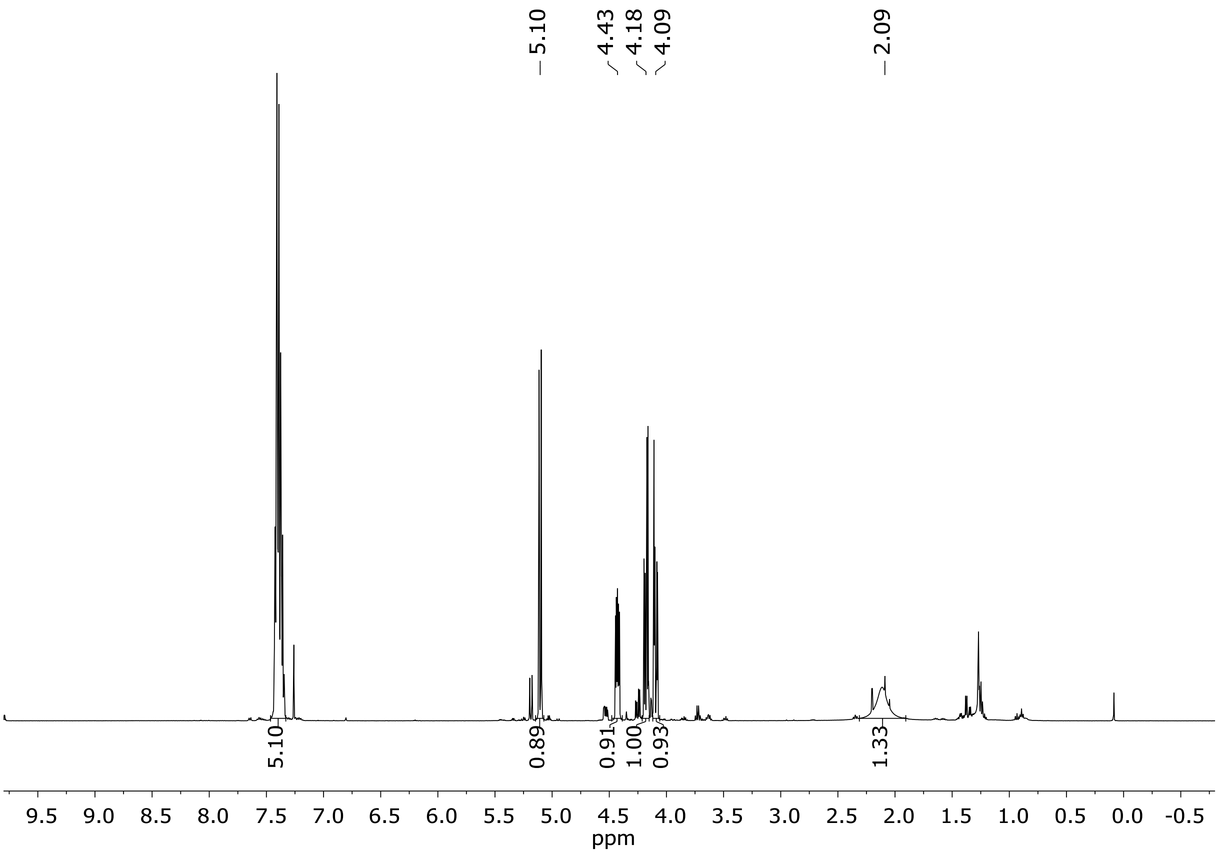

^{1}H NMR (500 MHz, $CDCl_3$) of compound **2l**.

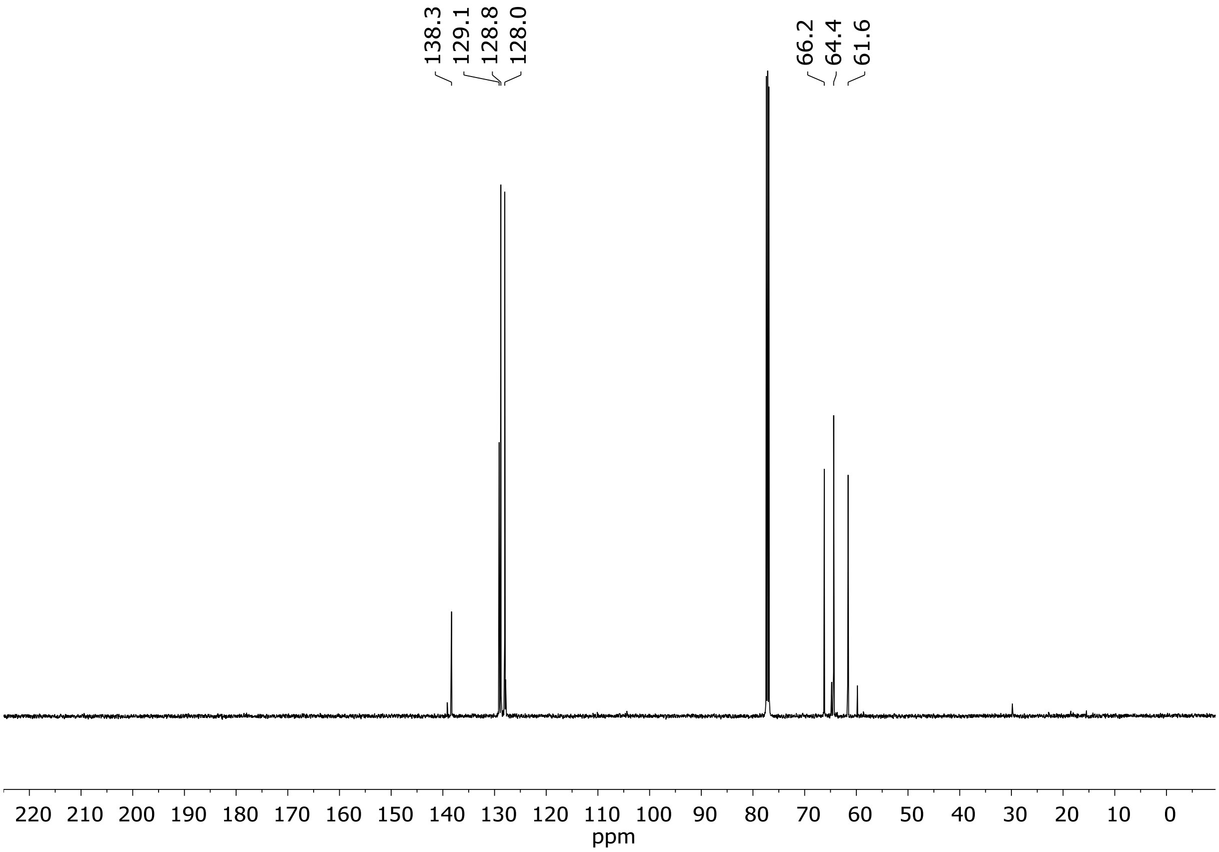

^{13}C NMR (125 MHz, $CDCl_3$) of compound **2l**.

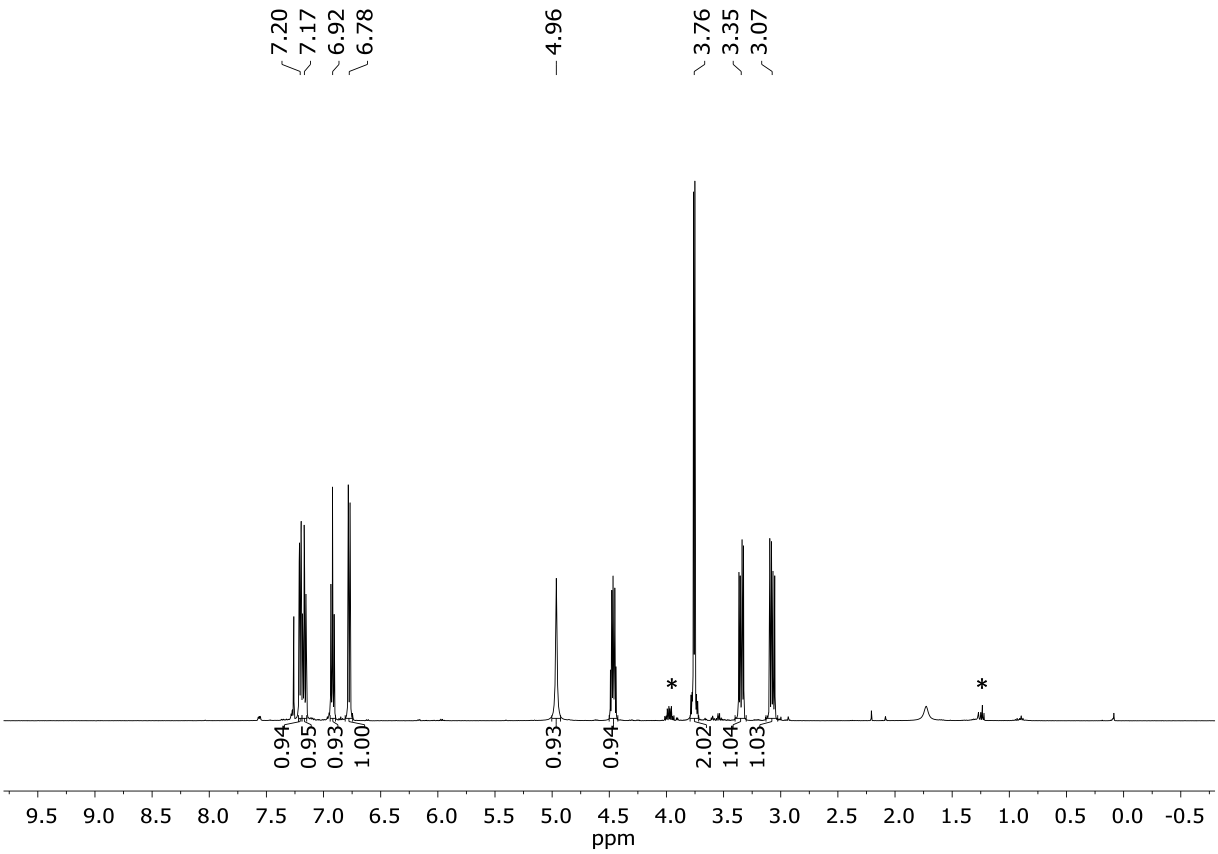

^{1}H NMR (500 MHz, $CDCl_3$) of compound **2m**. Residual solvent signals are marked.

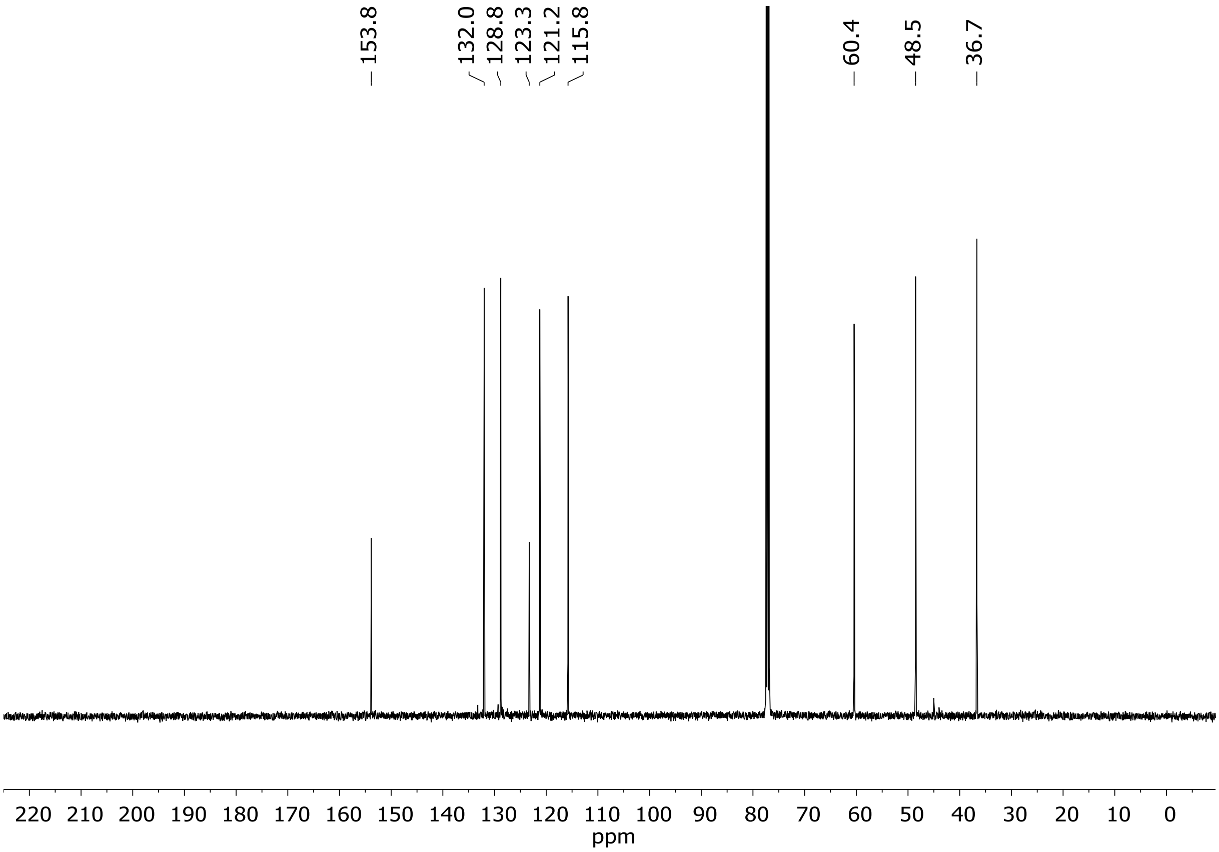

^{13}C NMR (125 MHz, $CDCl_3$) of compound **2m**.

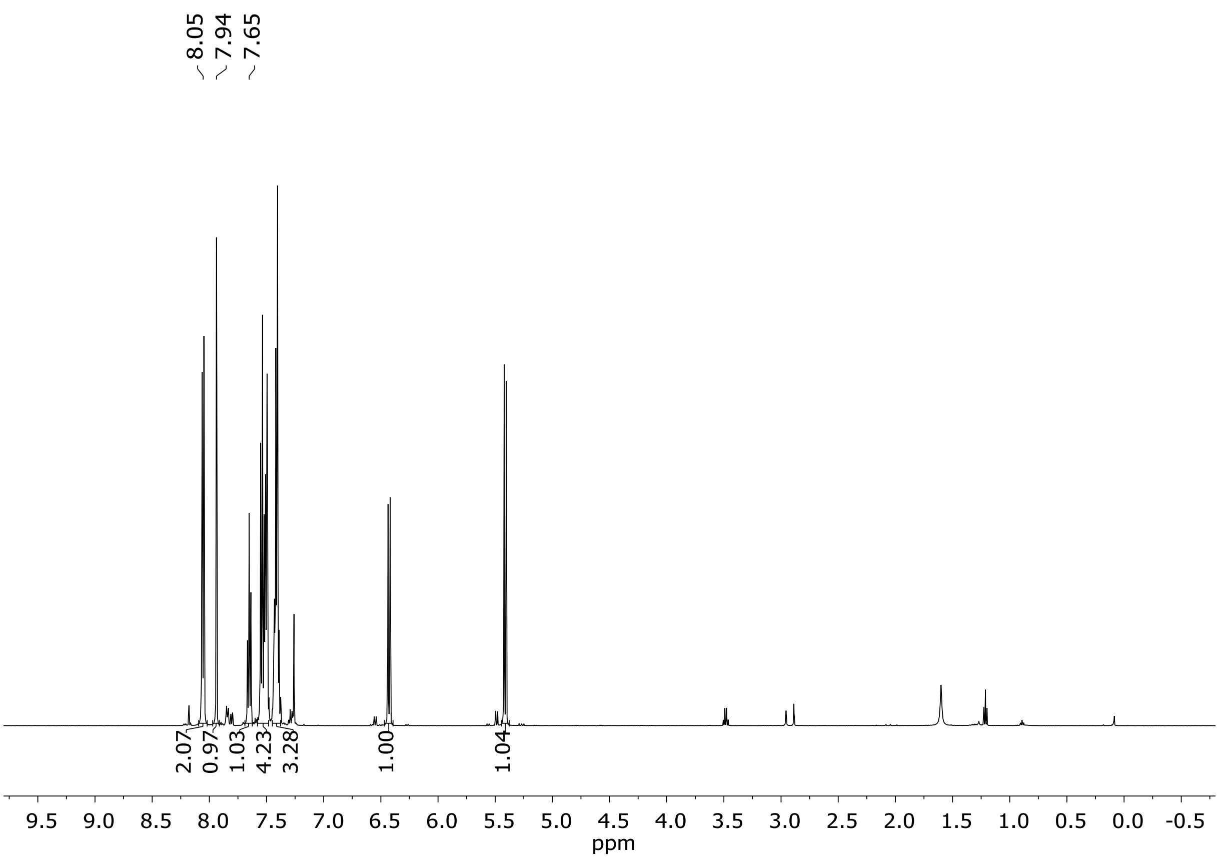

^{1}H NMR (500 MHz, $CDCl_3$) of compound **2n**.

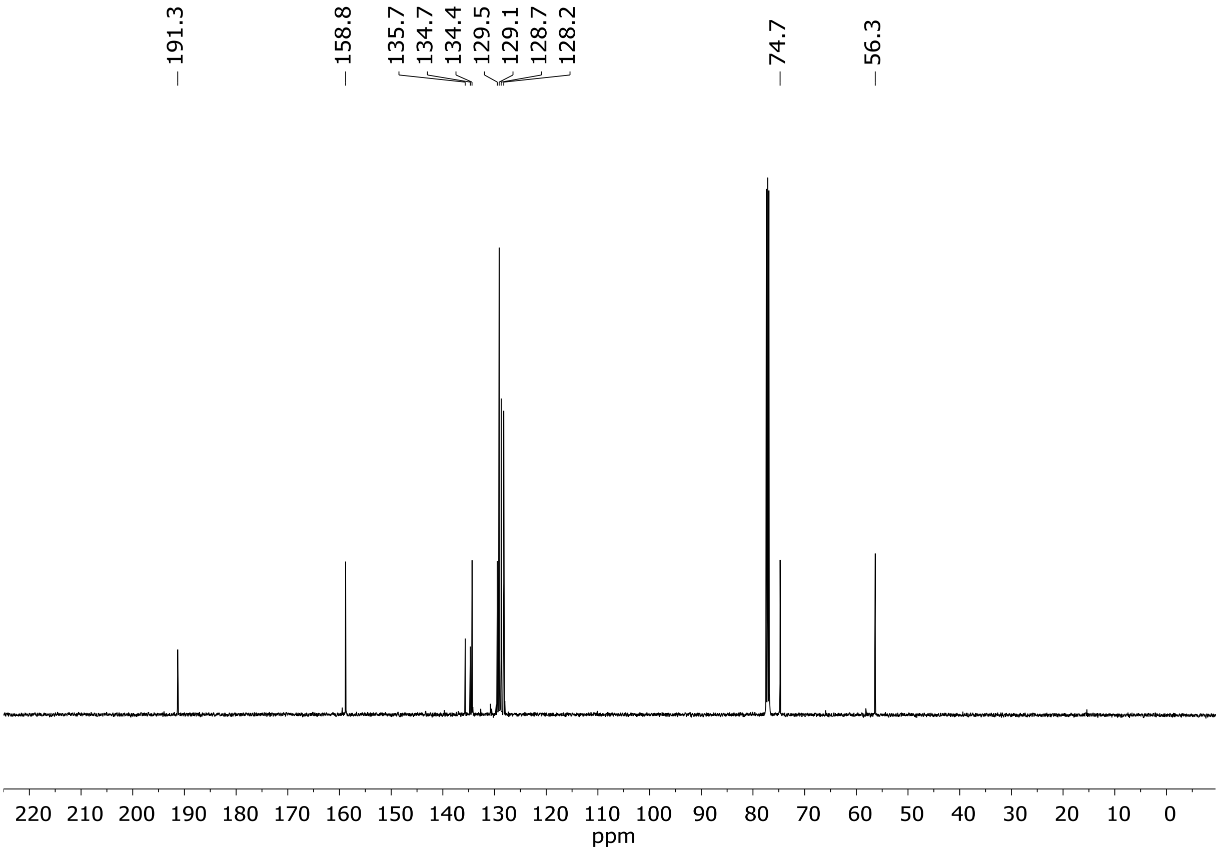

^{13}C NMR (125 MHz, $CDCl_3$) of compound **2n**.

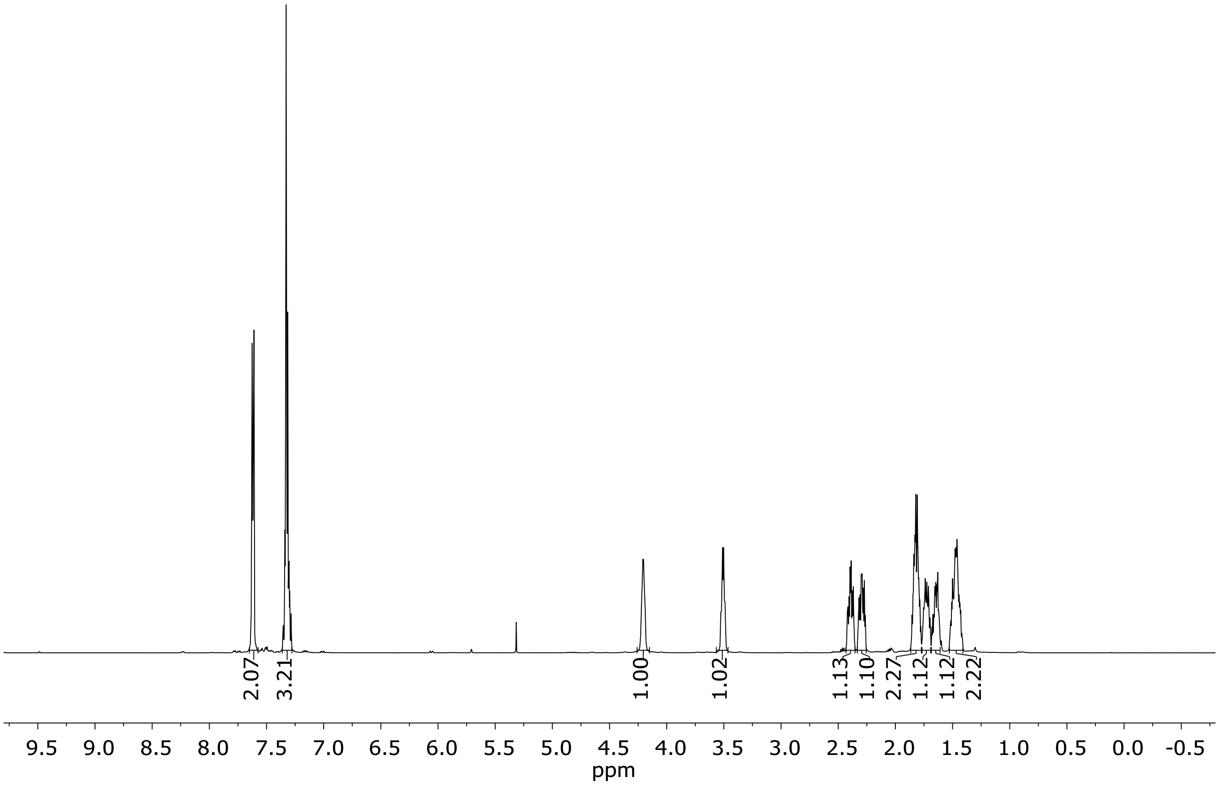

^{1}H NMR (500 MHz, $CDCl_3$) of compound **6**.

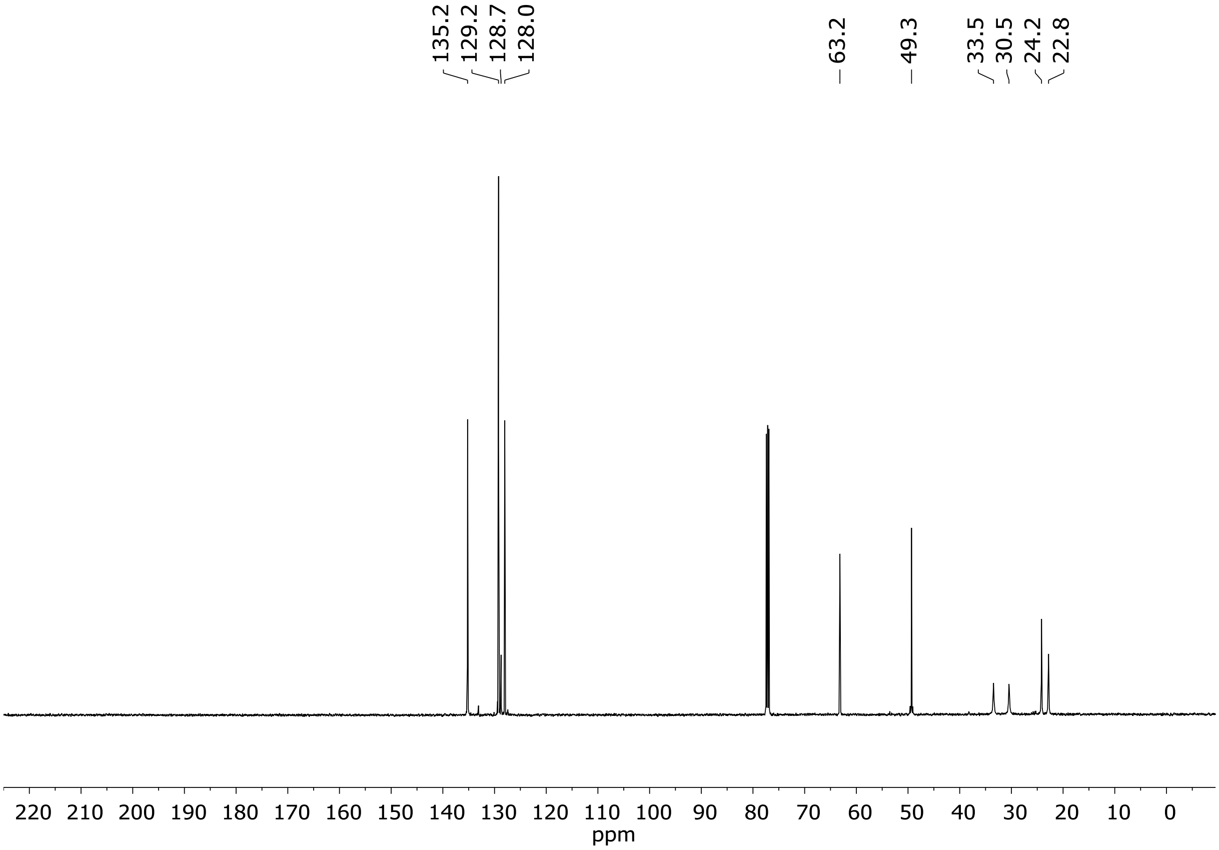

^{13}C NMR (125 MHz, $CDCl_3$) of compound **6**.

6. References

[1] G. R. Fulmer, A. J. M. Miller, N. H. Sherden, H. E. Gottlieb, A. Nudelman, B. M. Stoltz, J. E. Bercaw, K. I. Goldberg, *Organometallics* **2010**, *29*, 2176-2179.

[2] A. J. Cresswell, S. T. C. Eey, S. E. Denmark, *Nat. Chem.* **2015**, *7*, 146-152.

[3] N. Fu, G. S. Sauer, S. Lin, *J. Am. Chem. Soc.* **2017**, *139*, 15548-15553.

[4] A. H. Cleveland, F. R. Fronczek, R. Kartika, *J. Org. Chem.* **2018**, *83*, 3367-3377.

[5] M. L. Ho, A. B. Flynn, W. W. Ogilvie, *J. Org. Chem.* **2007**, *72*, 977-983.

[6] R. Ding, S. Huang, Q. Wang, Y. Liu, B. Sun, H. Tian, *Synth. Commun.* **2020**, *50*, 2319-2330.

[7] E. Langer, H. Lehner, K. Schlögl, *Tetrahedron* **1973**, *29*, 2473-2478.

[8] M. C. Cabaleiro, A. B. Chopa, *J. Chem. Soc., Perkin Trans. 2* **1974**, 452-457.

[9] S. E. Denmark, W. R. Collins, M. D. Cullen, *J. Am. Chem. Soc.* **2009**, *131*, 3490-3492.

[10] N. A. Barnes, S. M. Godfrey, R. T. Halton, R. G. Pritchard, *Dalton Trans.* **2005**, 1759-1761.

[11] D. Canestrari, S. Lancianesi, E. Badiola, C. Strinna, H. Ibrahim, M. F. Adamo, *Org. Lett.* **2017**, *19*, 918-921.
